# Supplementary material for: Nup107 is a crucial regulator of torso-mediated metamorphic transition in Drosophila melanogaster
Source: eLife. 2026 Mar 10;14:RP105165. doi: 10.7554/eLife.105165 (PMC12975125; doi:10.7554/eLife.105165)
Supplement: Figure 1—source data 1. [file elife-105165-fig1-data1.zip › Figure 1 source data 1/Figure 1 with 2 supplement.pdf]

1

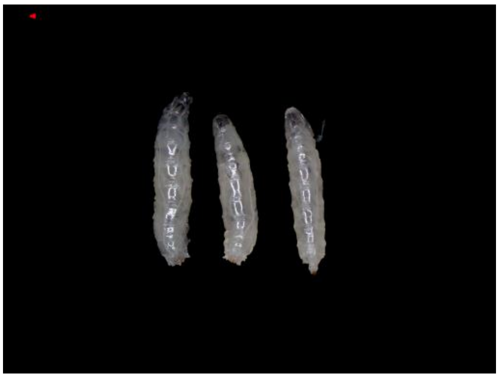

2

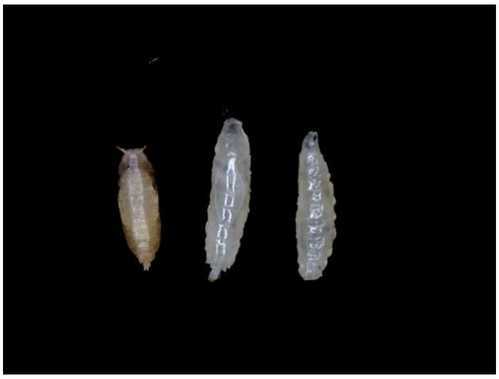

3

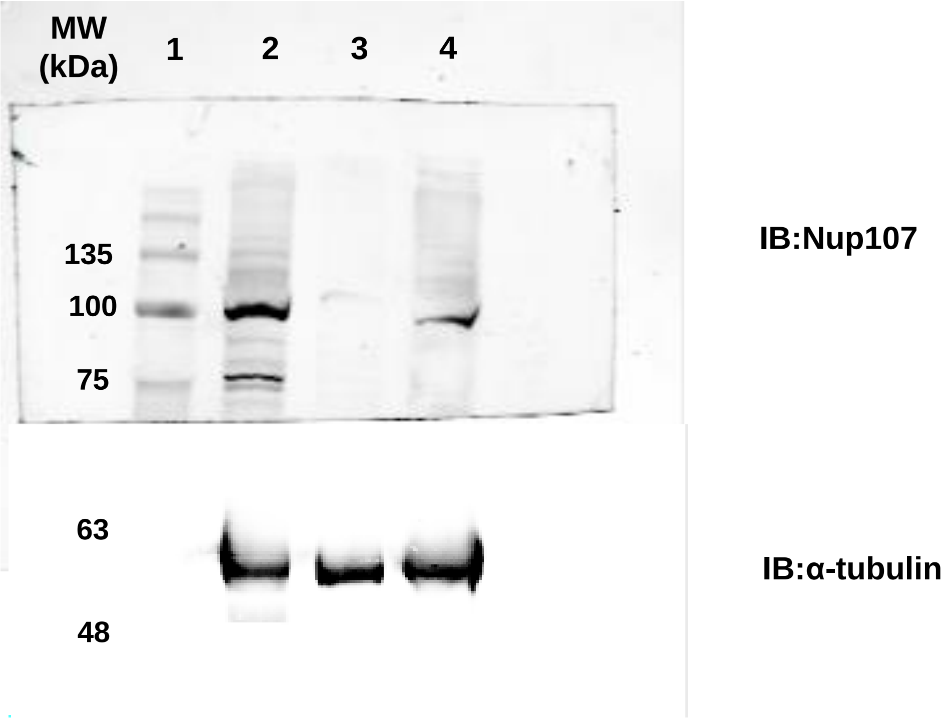


1. **Ladder**
2. **Control**
3. ***$Nup107^{GD}$***
4. ***$Nup107^{KK}$***

**Figure 1, Source Data 1.** Original images (1 & 2) corresponding to Figure 1A. Original, uncropped membranes corresponding to Figure 1C. Pre-stained protein ladder used as molecular weight markers. The upper membrane was probed with an anti-Nup107 antibody, while the lower membrane represents the loading control (α-tubulin).
