# Supplementary material for: Nup107 is a crucial regulator of torso-mediated metamorphic transition in Drosophila melanogaster
Source: eLife. 2026 Mar 10;14:RP105165. doi: 10.7554/eLife.105165 (PMC12975125; doi:10.7554/eLife.105165)
Supplement: Figure 1—source data 2. [file elife-105165-fig1-data2.zip › Figure 1 Source data 2/Figure 1 source data 1.pdf]

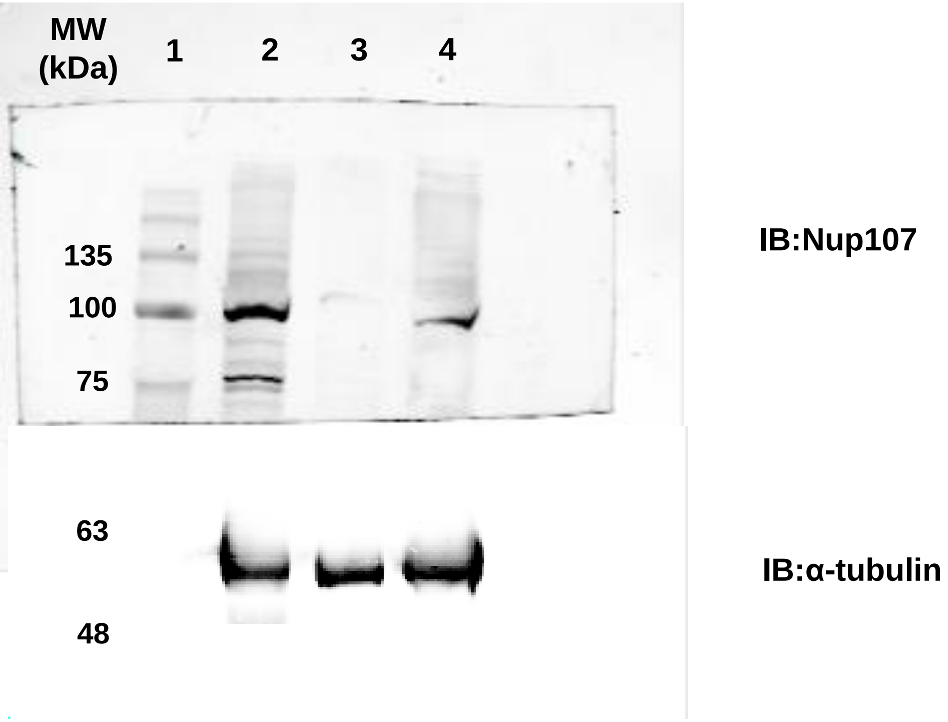

1. Ladder
2. Control
3. *Nup107^{GD}*
4. *Nup107^{KK}*

Figure 1- Source Data 1. Original, uncropped membranes corresponding to Figure 1C. Pre-stained protein ladder used as molecular weight markers. The upper membrane was probed with an anti-Nup107 antibody, while the lower membrane represents the loading control (α-tubulin).
