# Supplementary figures and images for: Nup107 is a crucial regulator of torso-mediated metamorphic transition in Drosophila melanogaster
Source: eLife. 2026 Mar 10;14:RP105165. doi: 10.7554/eLife.105165 (PMC12975125; doi:10.7554/eLife.105165)

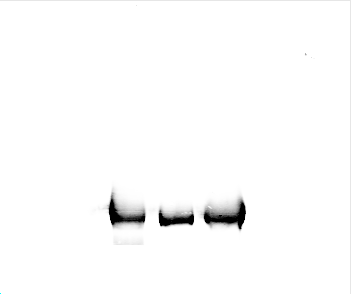

Supplement: Figure 1—source data 2. [file elife-105165-fig1-data2.zip › Figure 1 Source data 2/tub.tif]

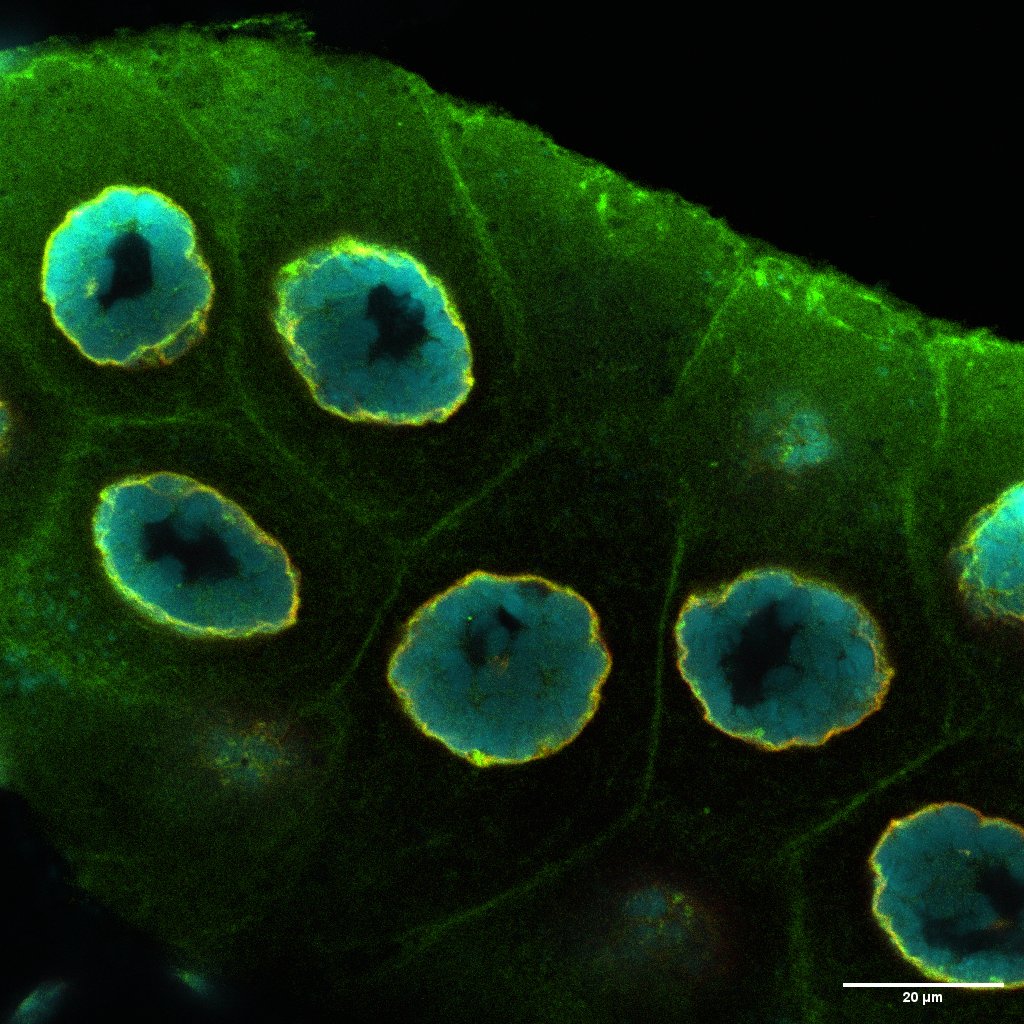

Supplement: Figure 1—figure supplement 1—source data 2. [file elife-105165-fig1-figsupp1-data2.zip › Figure 1-figure supplement 1_Source data 2/S1B_.jpg]

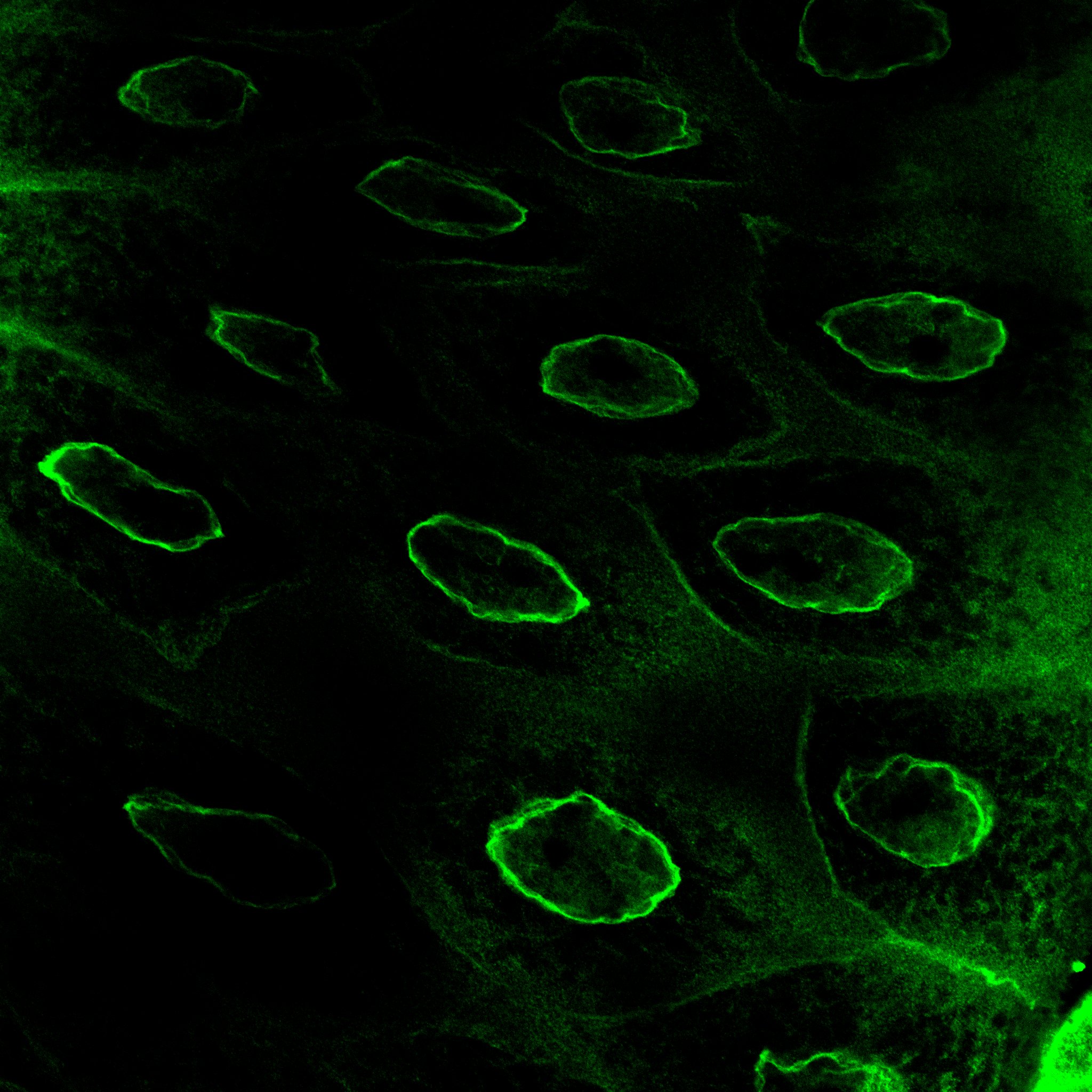

Supplement: Figure 1—figure supplement 1—source data 2. [file elife-105165-fig1-figsupp1-data2.zip › Figure 1-figure supplement 1_Source data 2/S1A_C2.jpg]

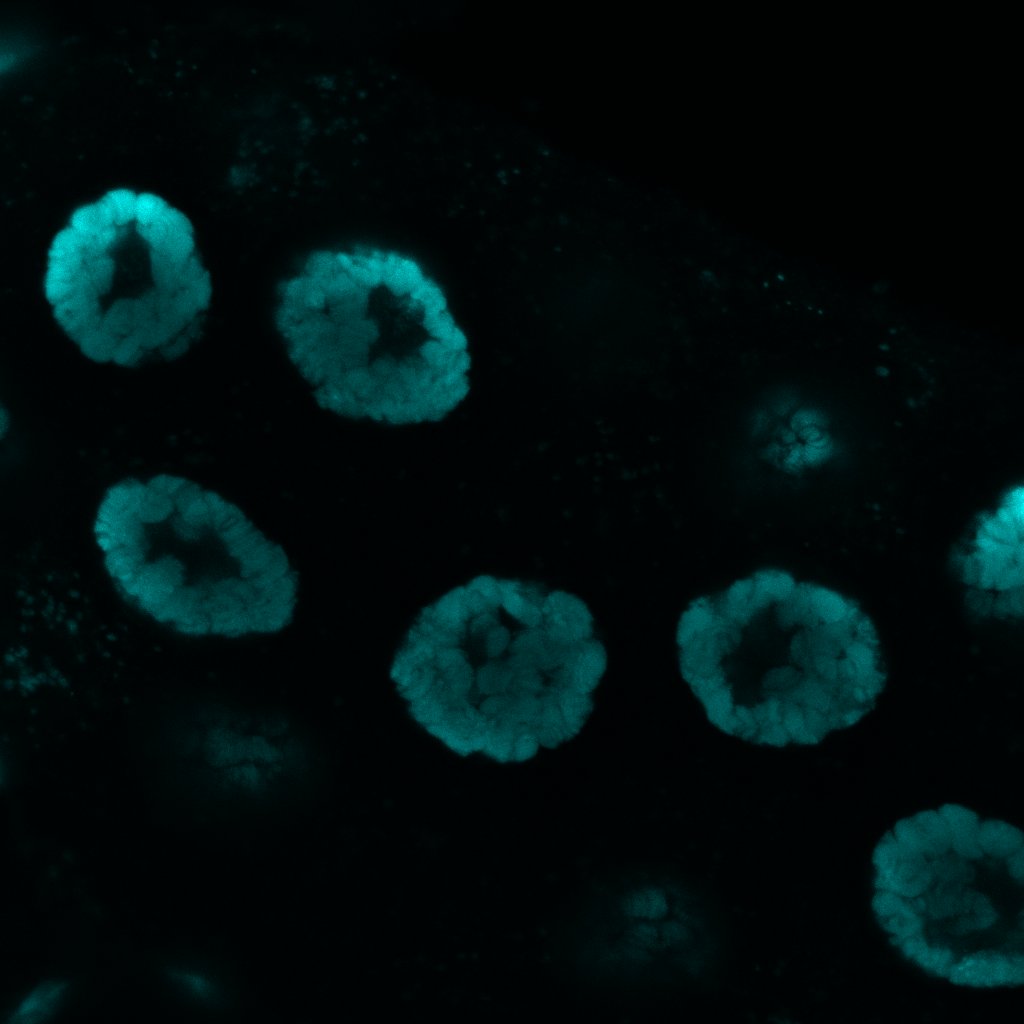

Supplement: Figure 1—figure supplement 1—source data 2. [file elife-105165-fig1-figsupp1-data2.zip › Figure 1-figure supplement 1_Source data 2/S1B_C1.jpg]

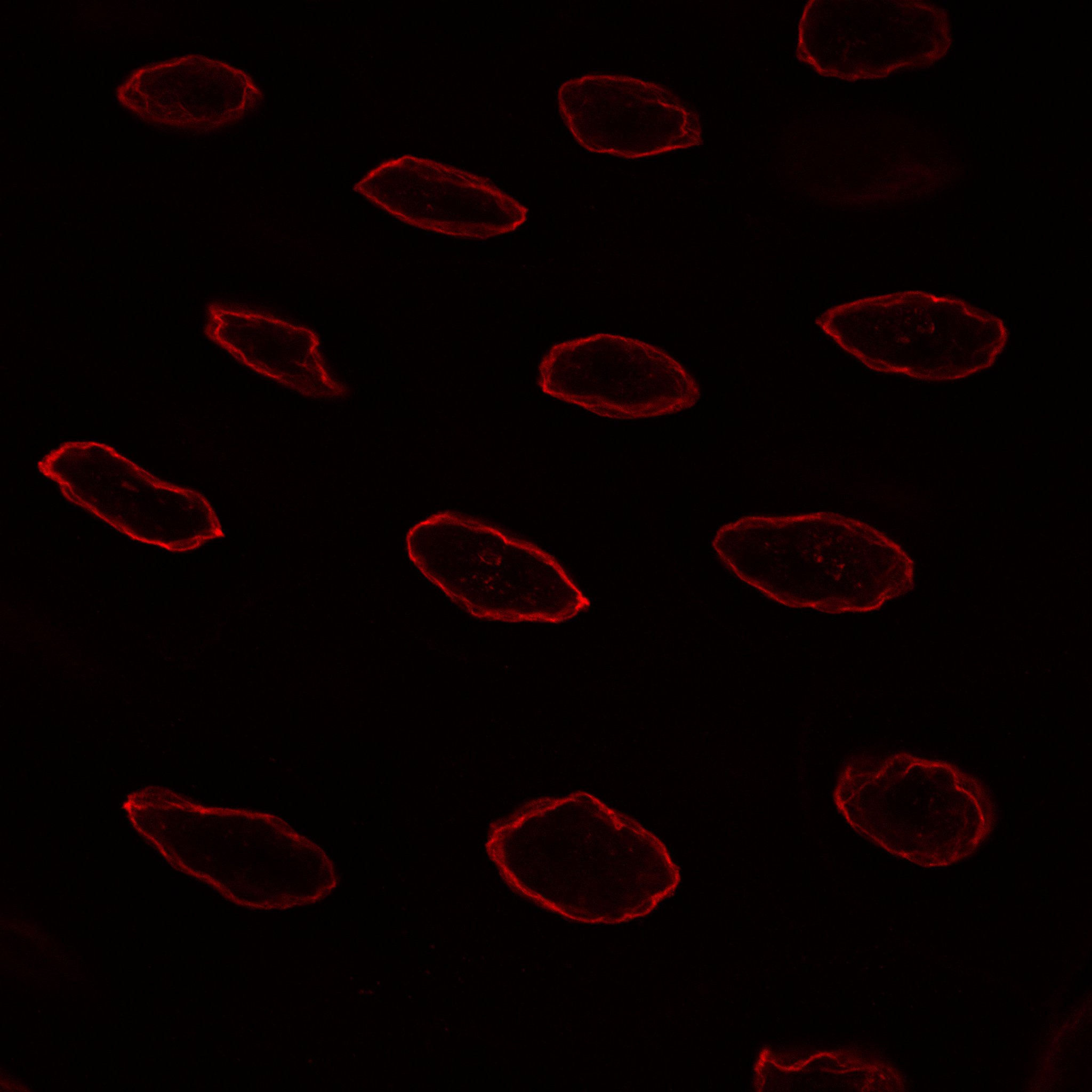

Supplement: Figure 1—figure supplement 1—source data 2. [file elife-105165-fig1-figsupp1-data2.zip › Figure 1-figure supplement 1_Source data 2/S1A_C3.jpg]

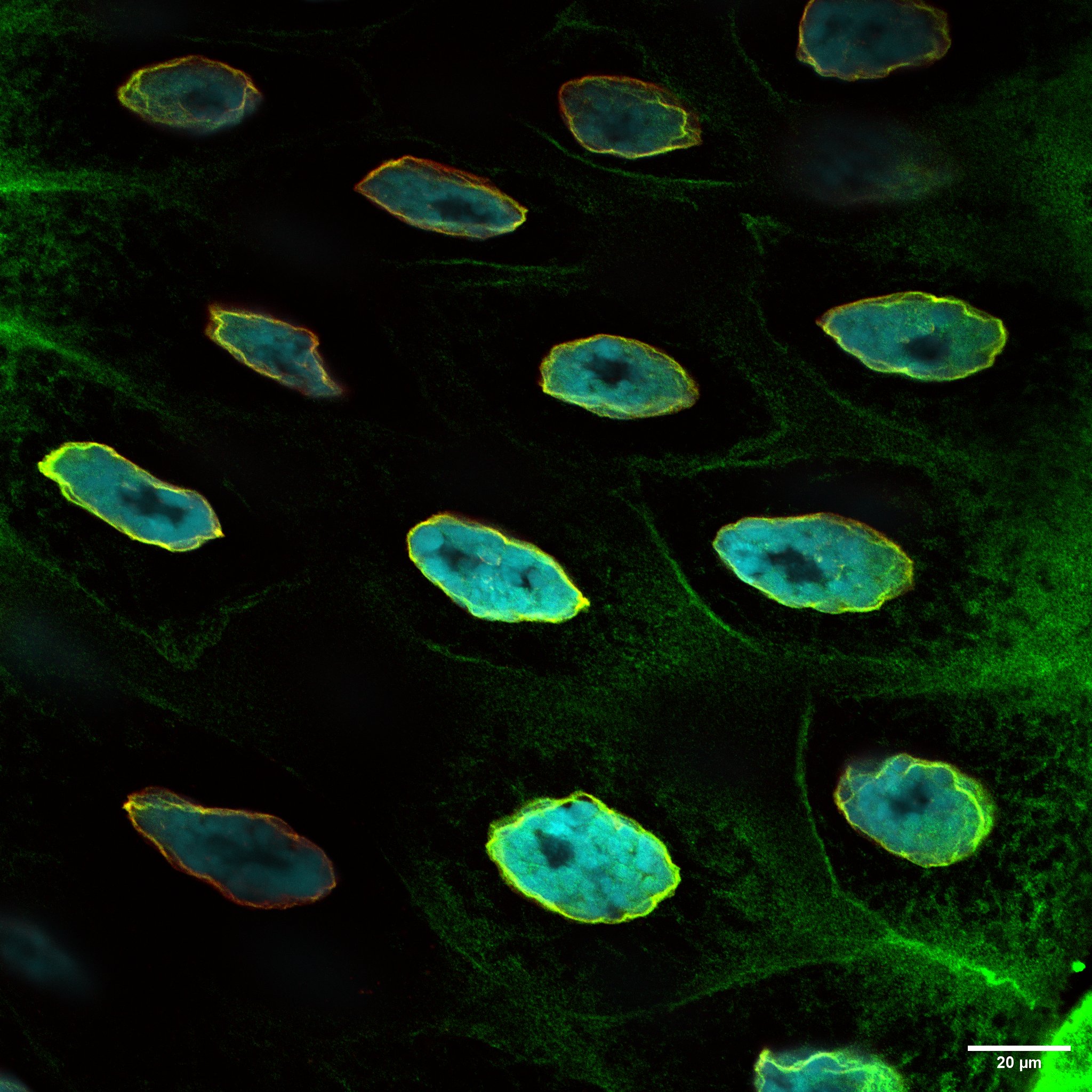

Supplement: Figure 1—figure supplement 1—source data 2. [file elife-105165-fig1-figsupp1-data2.zip › Figure 1-figure supplement 1_Source data 2/S1A_.jpg]

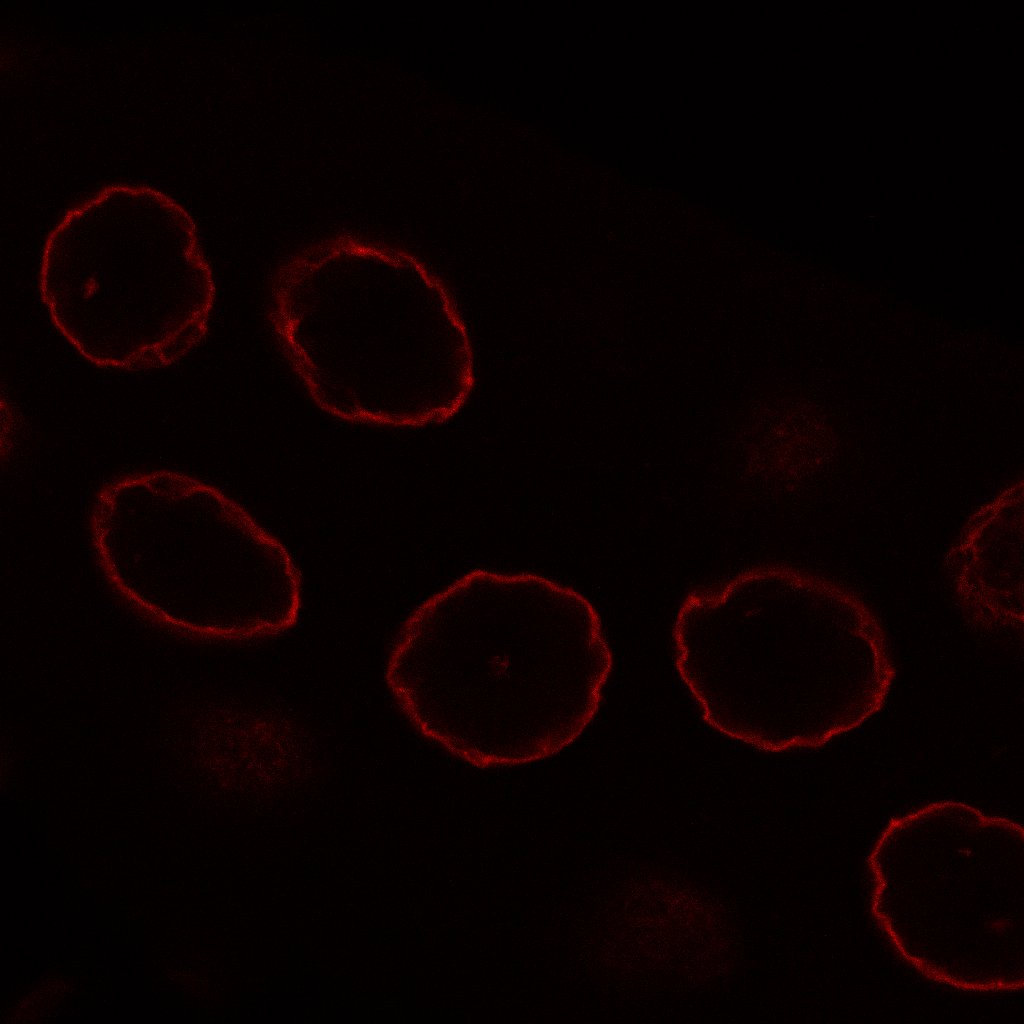

Supplement: Figure 1—figure supplement 1—source data 2. [file elife-105165-fig1-figsupp1-data2.zip › Figure 1-figure supplement 1_Source data 2/S1B_C2.jpg]

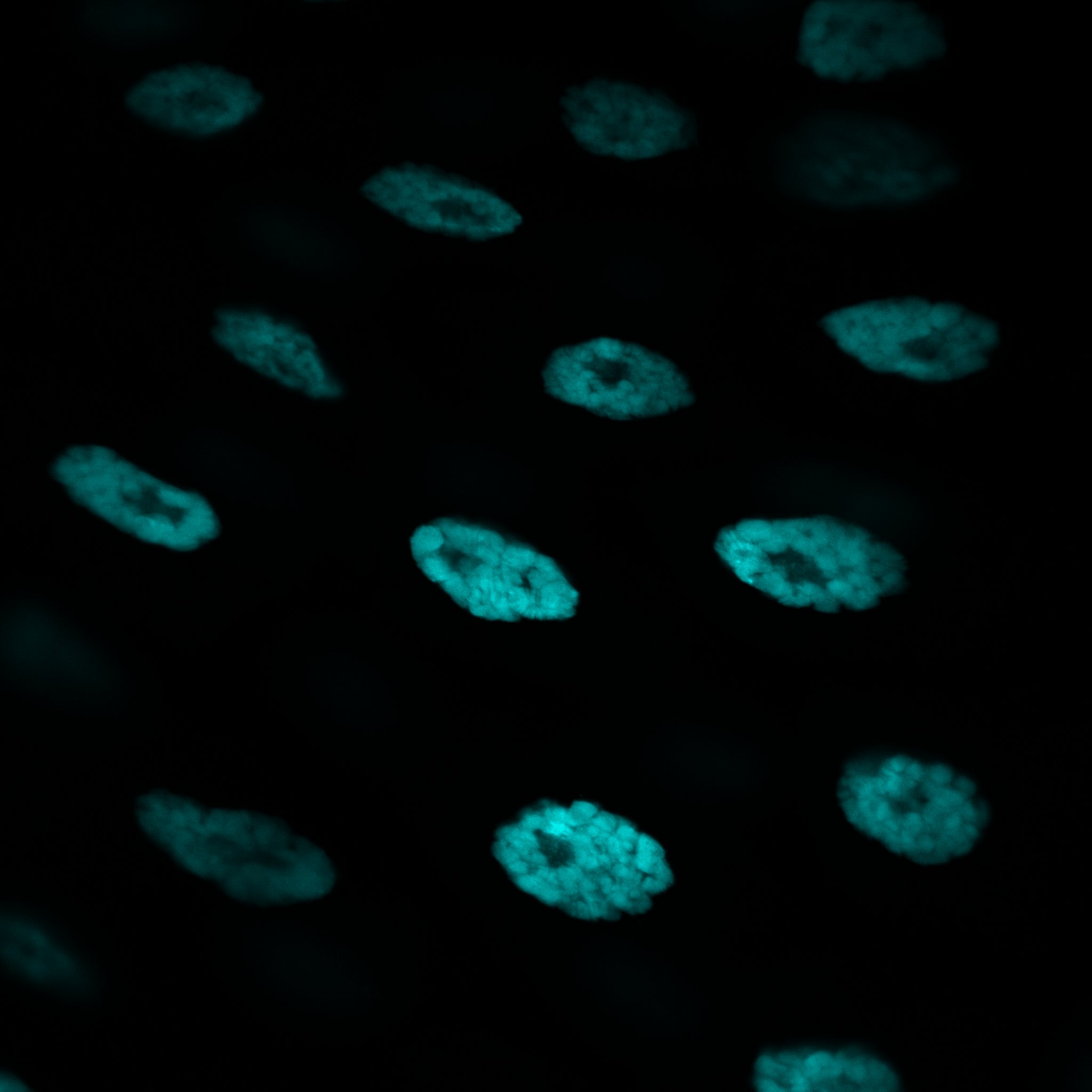

Supplement: Figure 1—figure supplement 1—source data 2. [file elife-105165-fig1-figsupp1-data2.zip › Figure 1-figure supplement 1_Source data 2/S1A_C1.jpg]

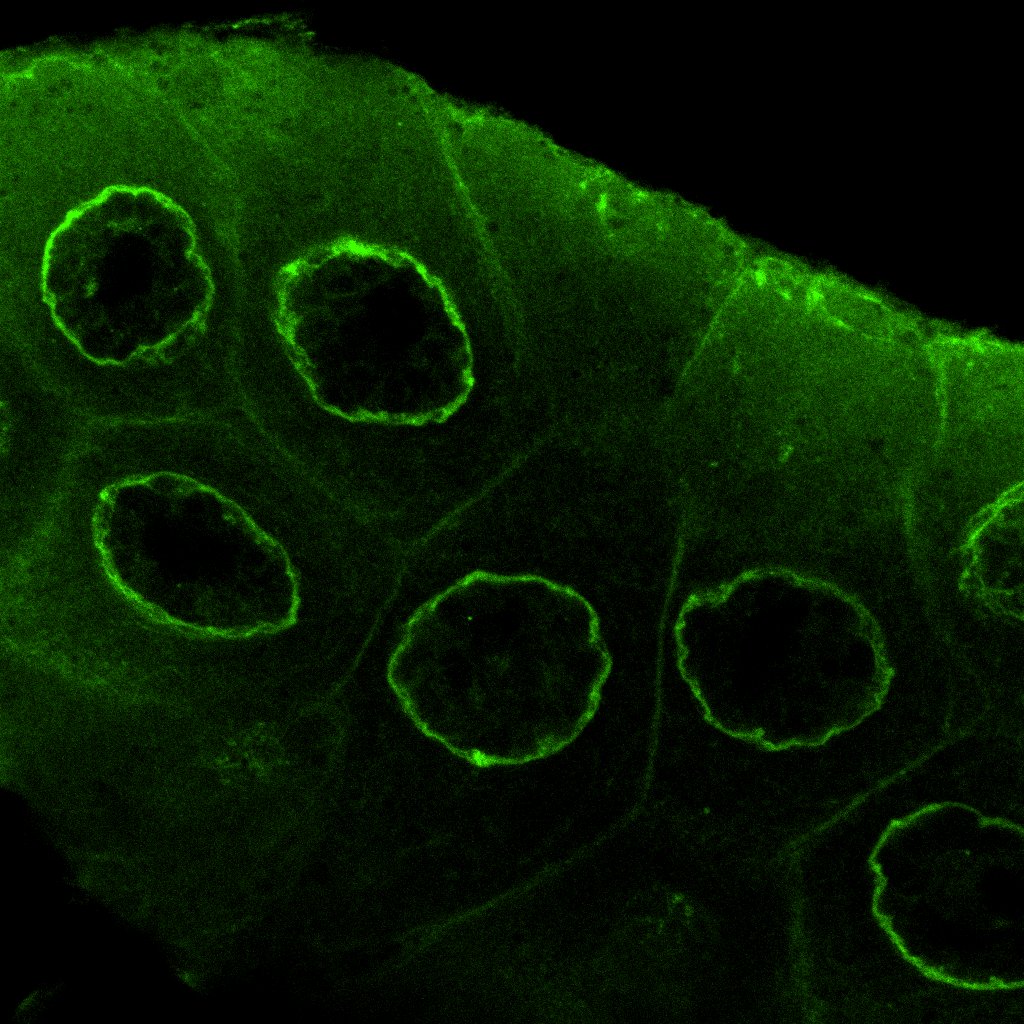

Supplement: Figure 1—figure supplement 1—source data 2. [file elife-105165-fig1-figsupp1-data2.zip › Figure 1-figure supplement 1_Source data 2/S1B_C3.jpg]

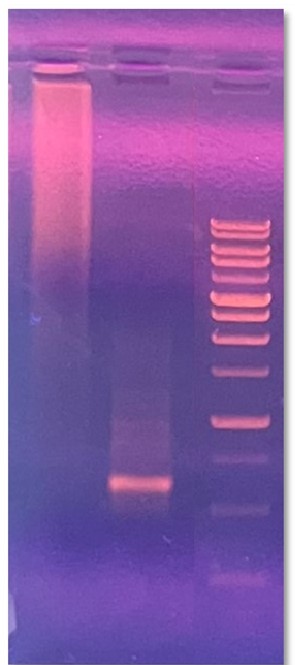

Supplement: Figure 1—figure supplement 2—source data 2. [file elife-105165-fig1-figsupp2-data2.zip › Figure 1-figure supplement 2_Source data 2/S2.jpg]

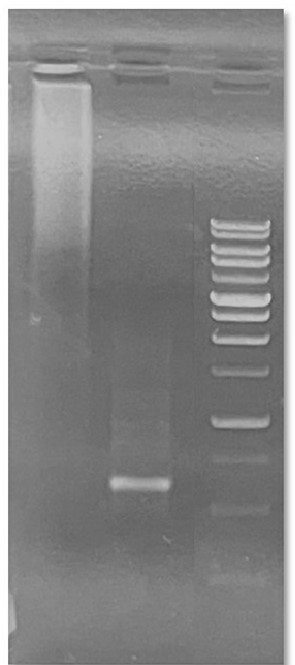

Supplement: Figure 1—figure supplement 2—source data 2. [file elife-105165-fig1-figsupp2-data2.zip › Figure 1-figure supplement 2_Source data 2/Gel Black and white.tif]

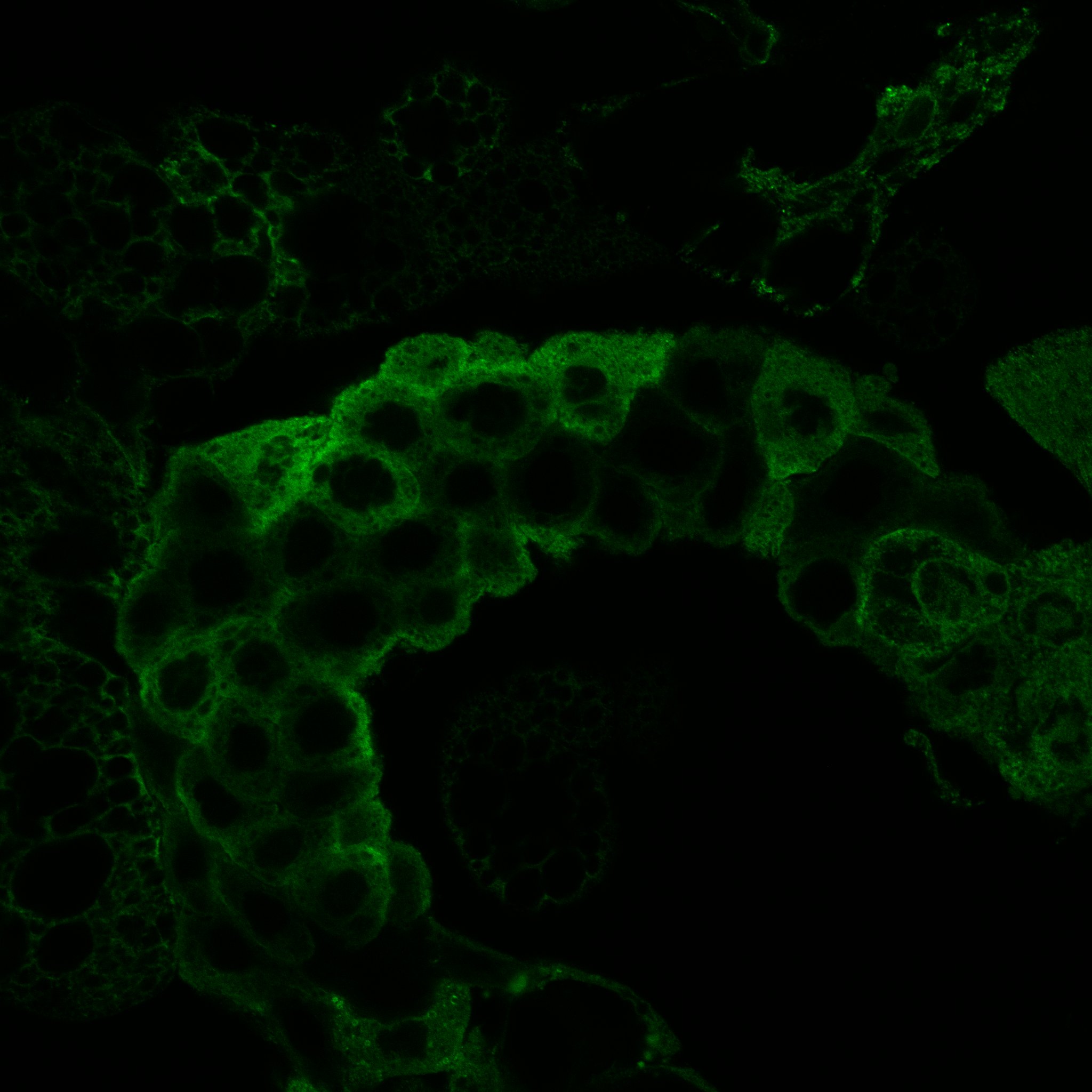

Supplement: Figure 2—source data 2. [file elife-105165-fig2-data2.zip › Figure 2 source data 2/2B_C2.jpg]

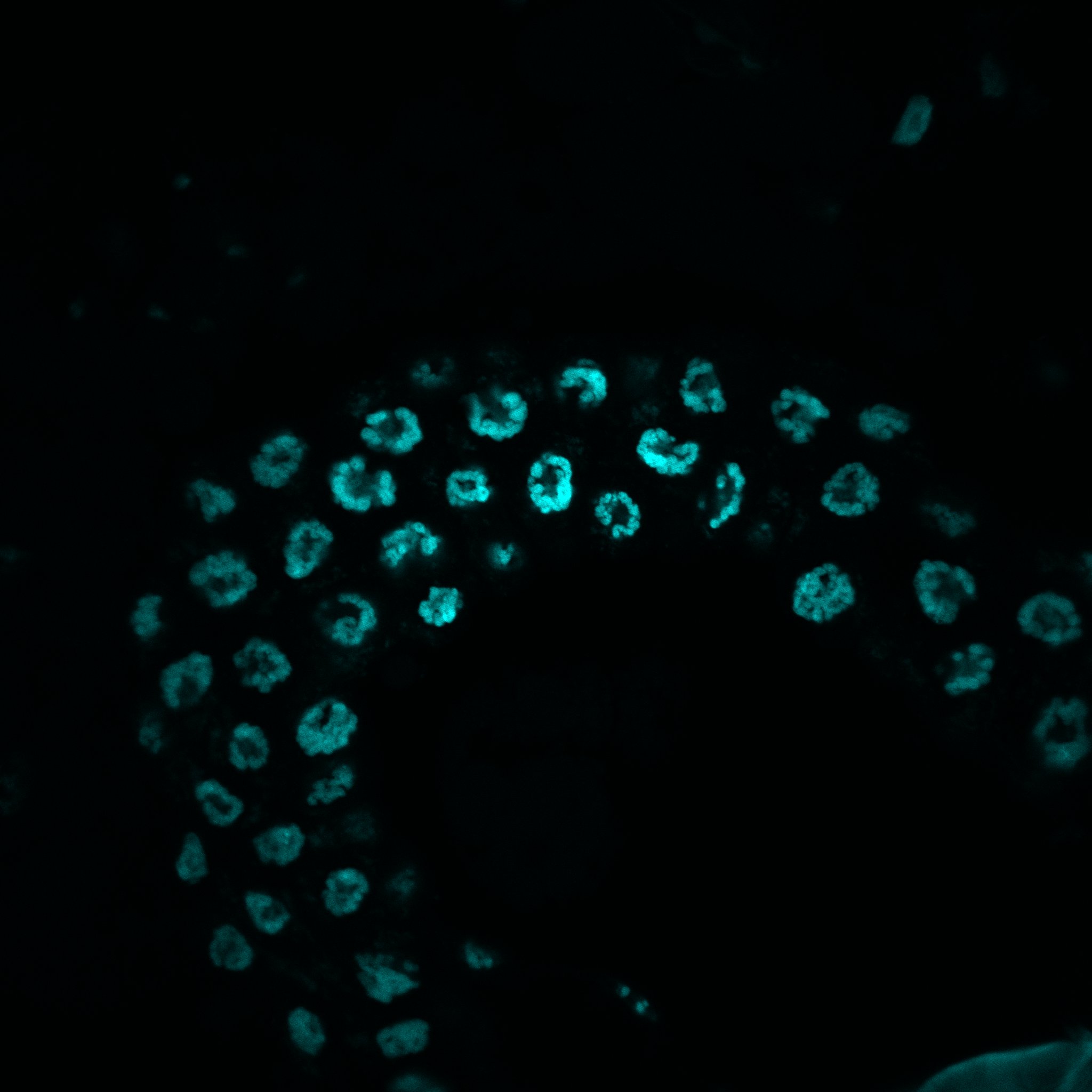

Supplement: Figure 2—source data 2. [file elife-105165-fig2-data2.zip › Figure 2 source data 2/2B_C1.jpg]

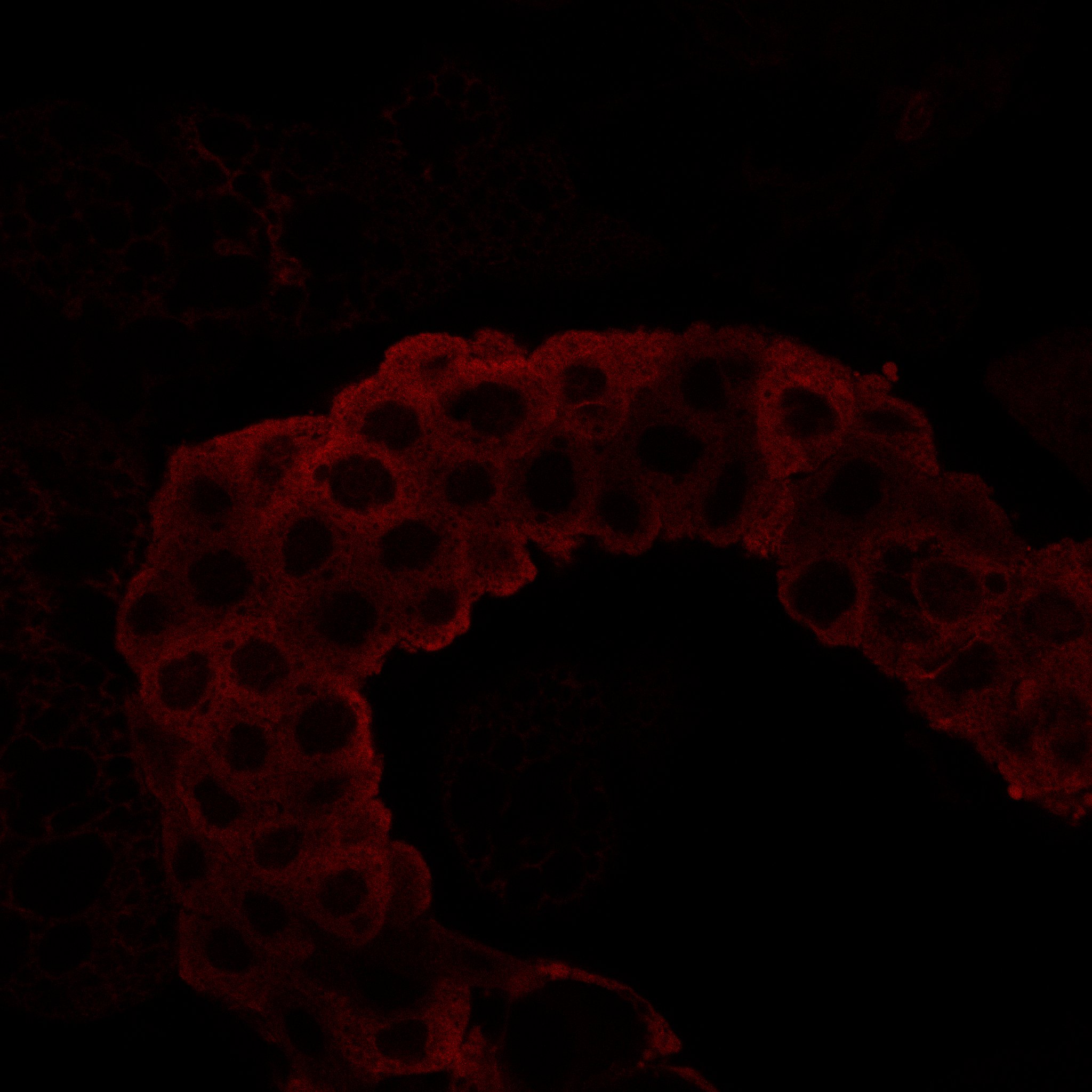

Supplement: Figure 2—source data 2. [file elife-105165-fig2-data2.zip › Figure 2 source data 2/2B_C3.jpg]

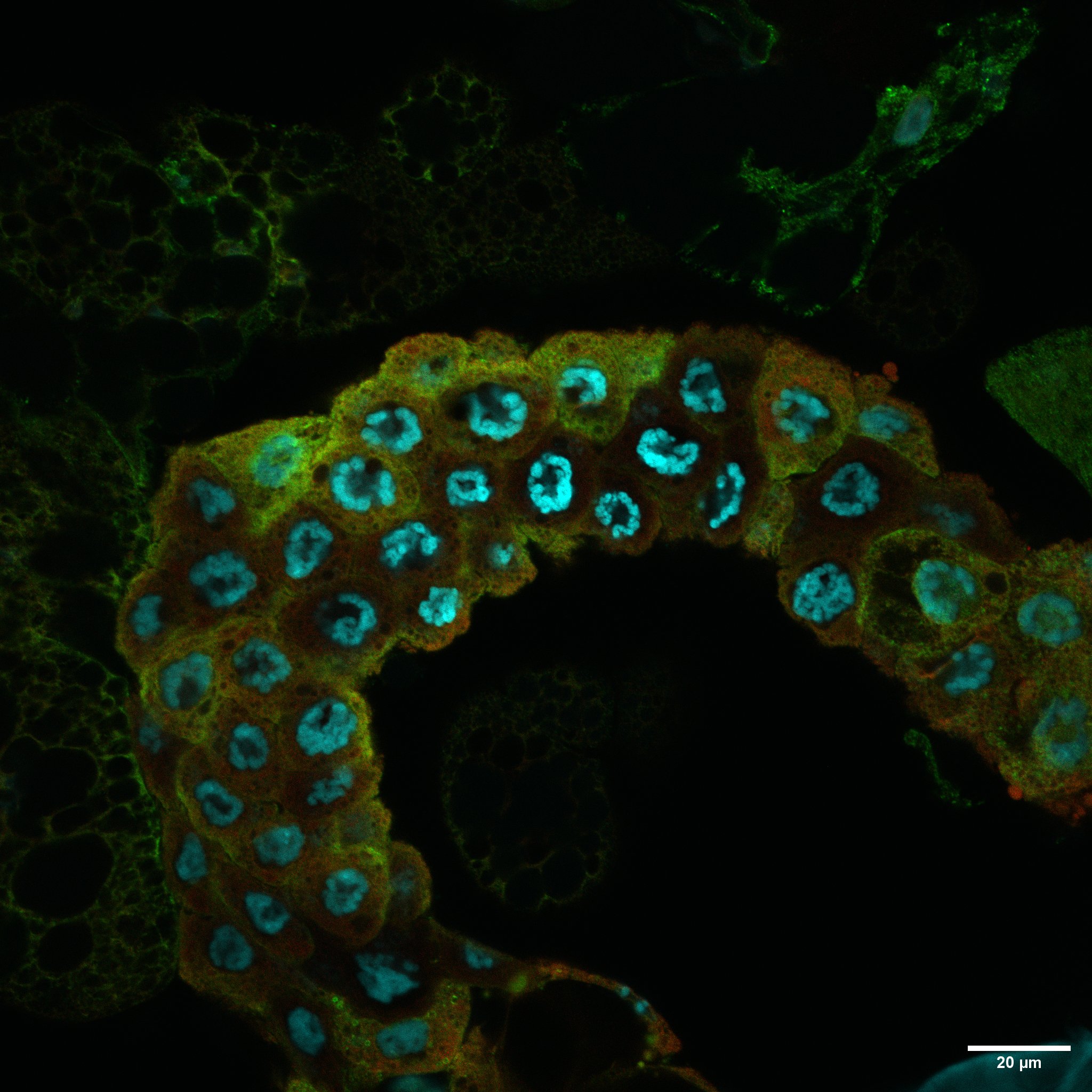

Supplement: Figure 2—source data 2. [file elife-105165-fig2-data2.zip › Figure 2 source data 2/2B_.jpg]

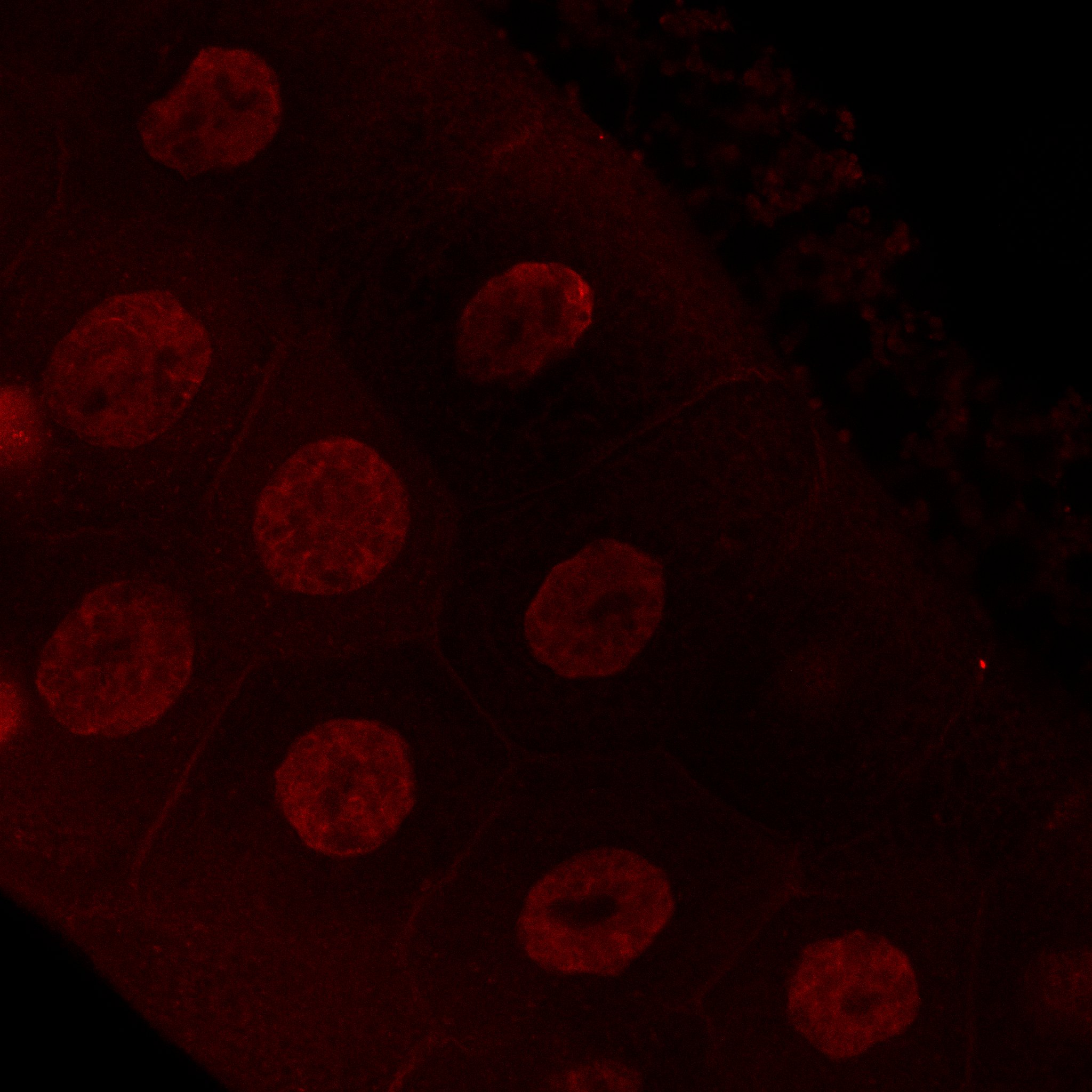

Supplement: Figure 2—source data 2. [file elife-105165-fig2-data2.zip › Figure 2 source data 2/2A_C3.jpg]

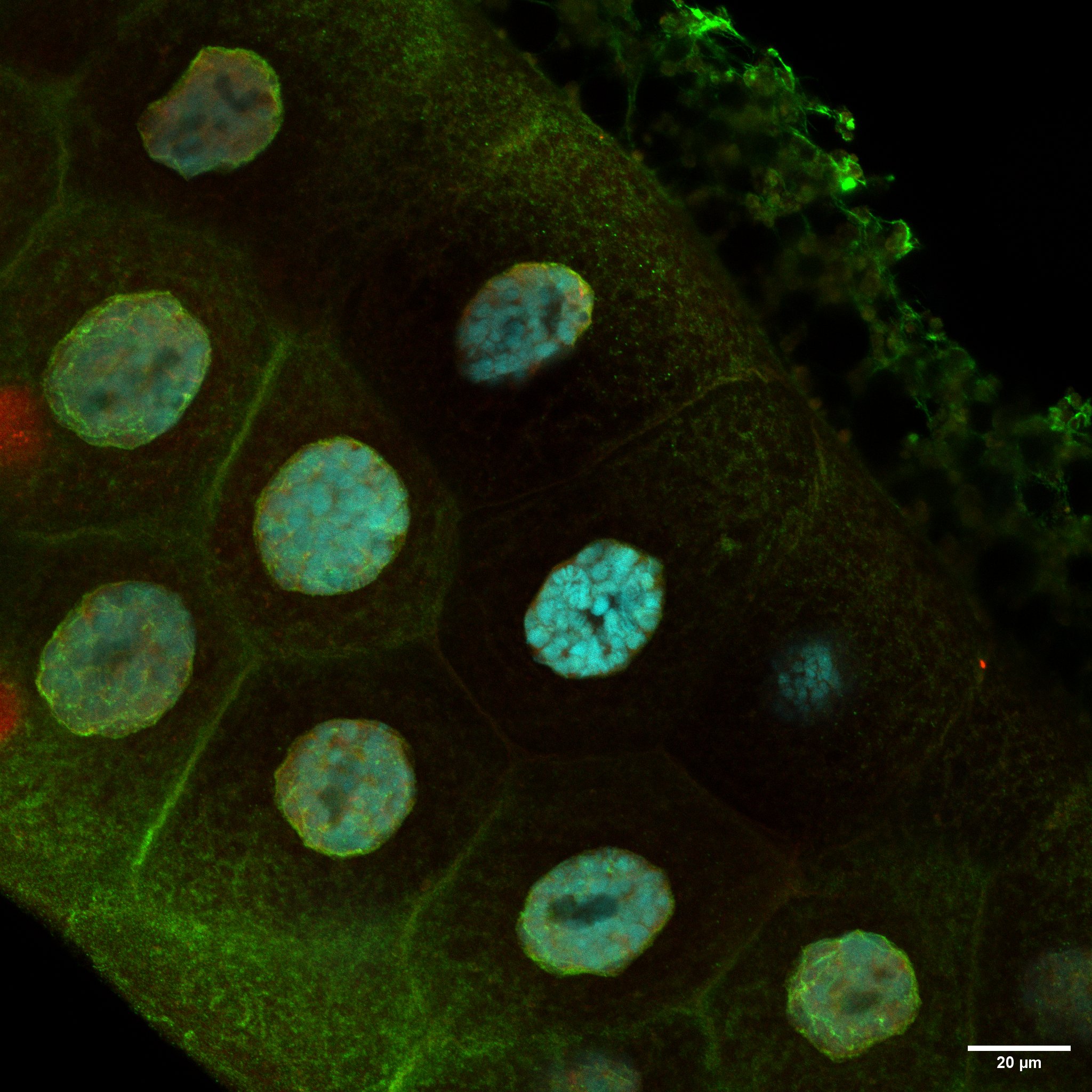

Supplement: Figure 2—source data 2. [file elife-105165-fig2-data2.zip › Figure 2 source data 2/2A_.jpg]

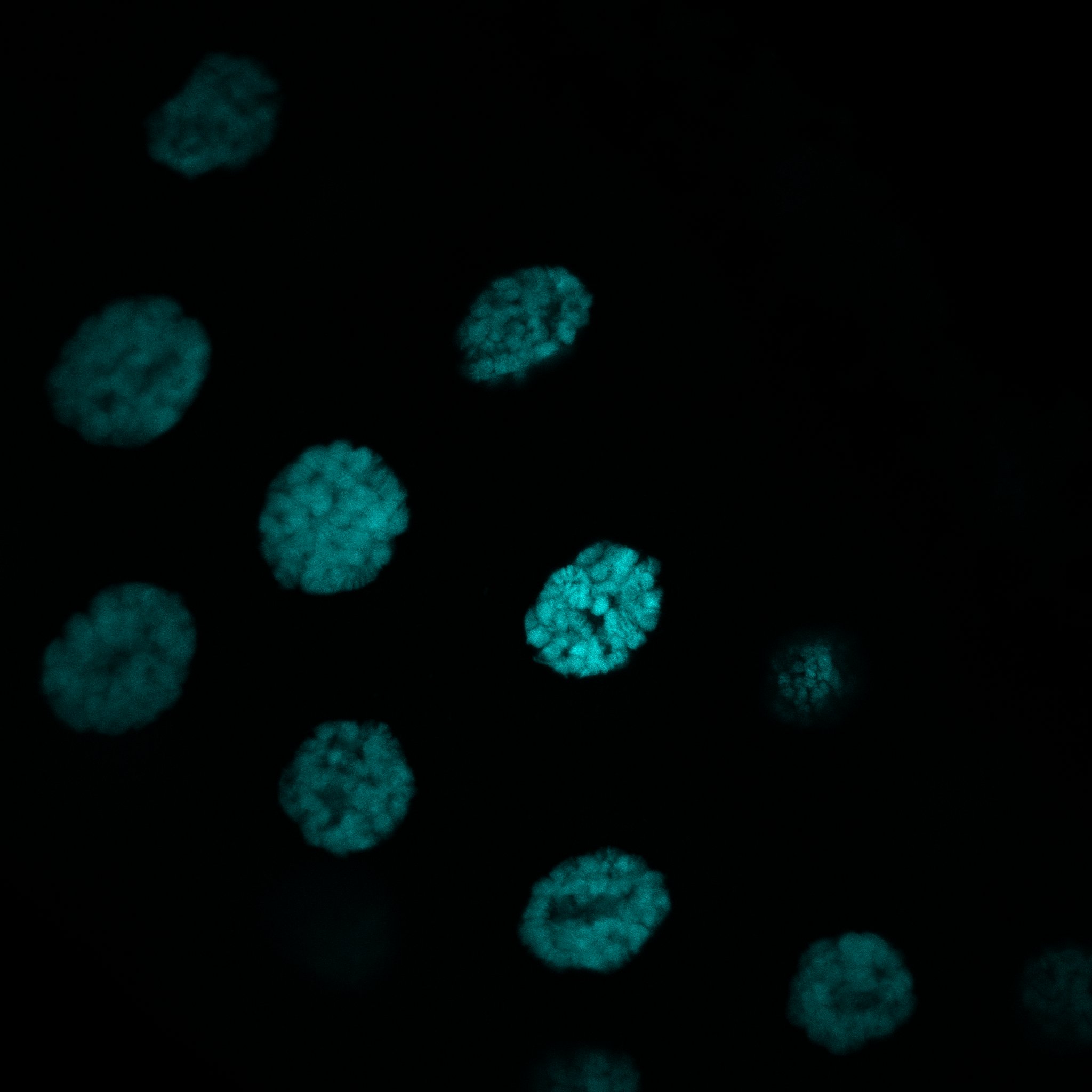

Supplement: Figure 2—source data 2. [file elife-105165-fig2-data2.zip › Figure 2 source data 2/2A_C1.jpg]

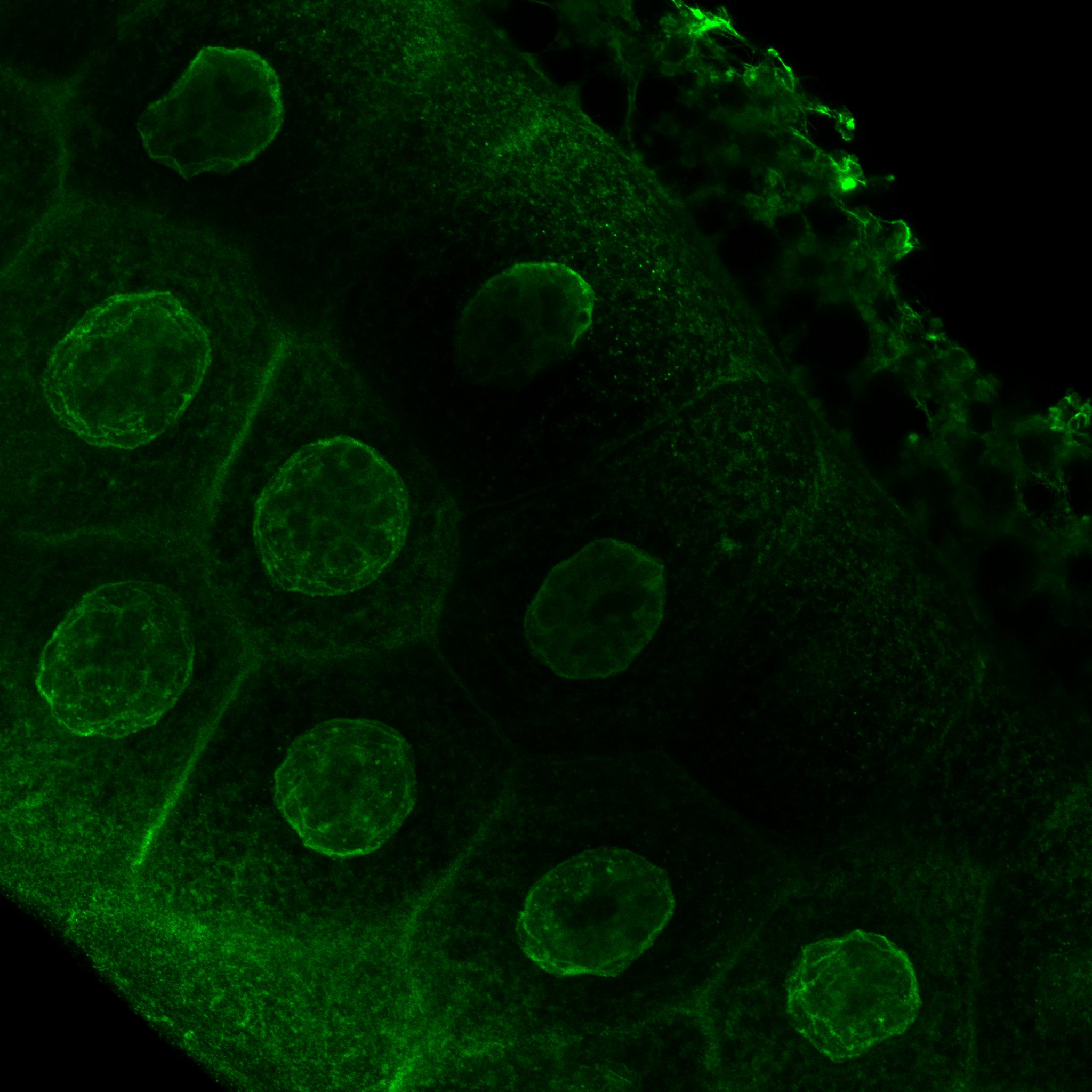

Supplement: Figure 2—source data 2. [file elife-105165-fig2-data2.zip › Figure 2 source data 2/2A_C2.jpg]

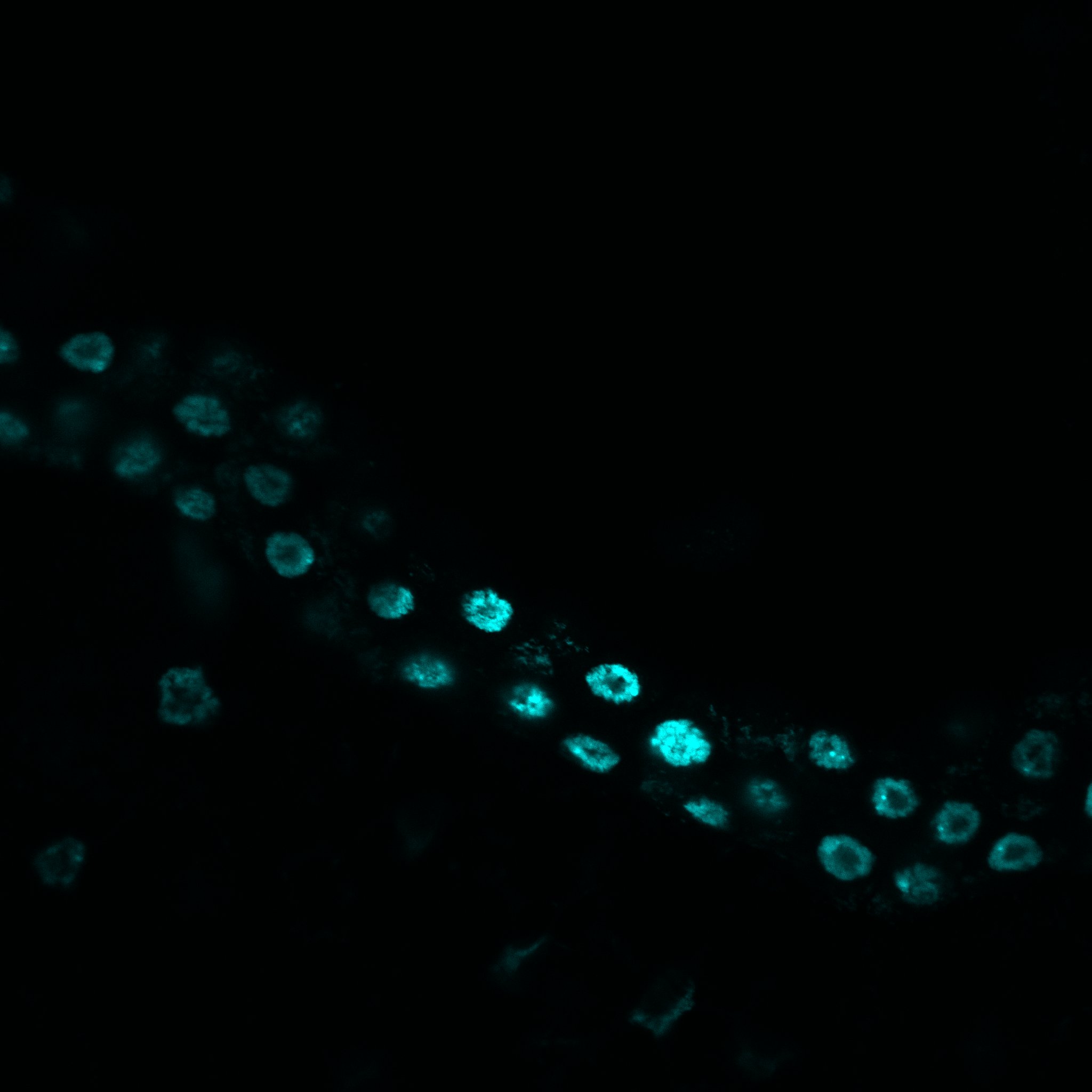

Supplement: Figure 2—figure supplement 2—source data 2. [file elife-105165-fig2-figsupp2-data2.zip › Figure 2-figure supplement 2_Source data 2/S4_B_C1.jpg]

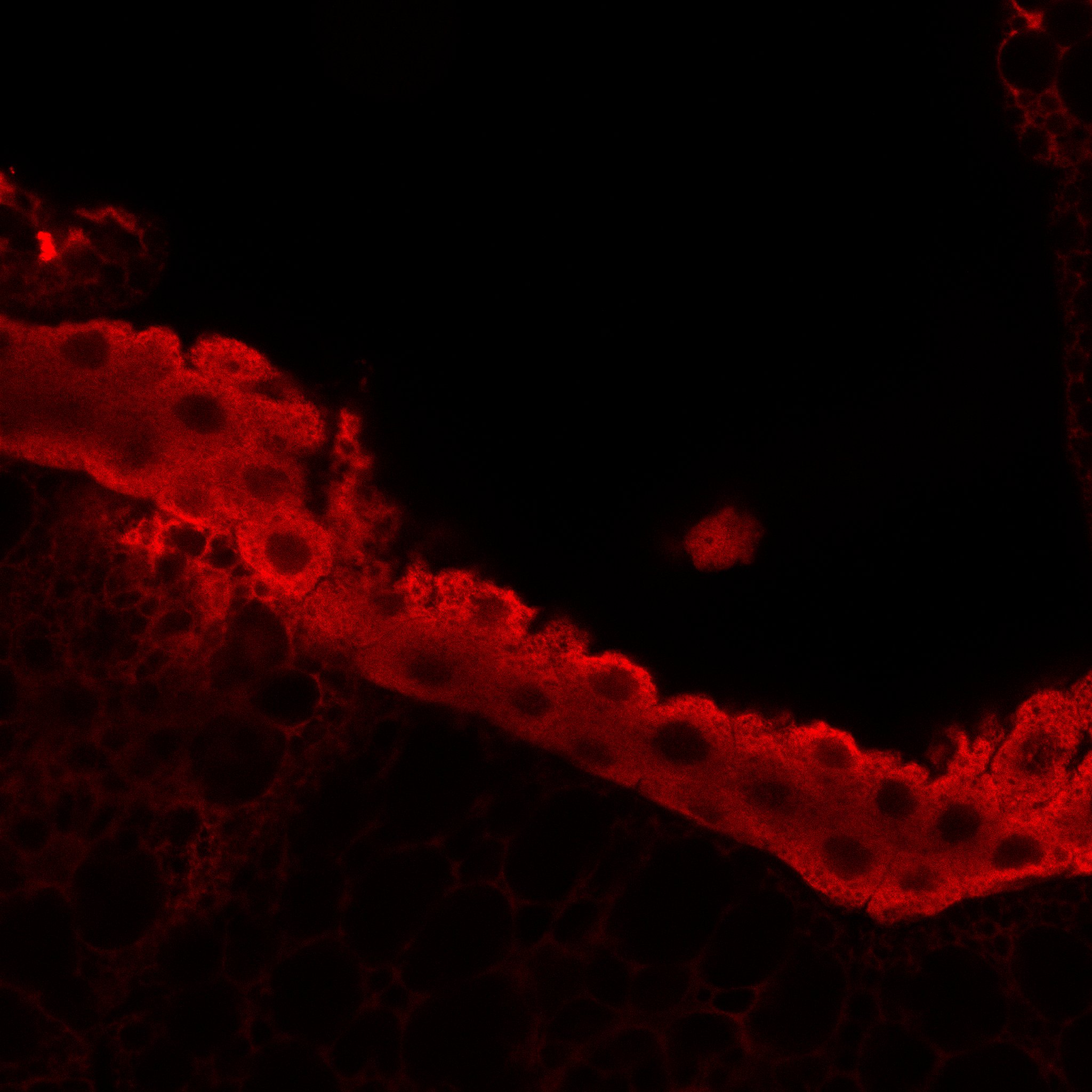

Supplement: Figure 2—figure supplement 2—source data 2. [file elife-105165-fig2-figsupp2-data2.zip › Figure 2-figure supplement 2_Source data 2/S4_B_C3.jpg]

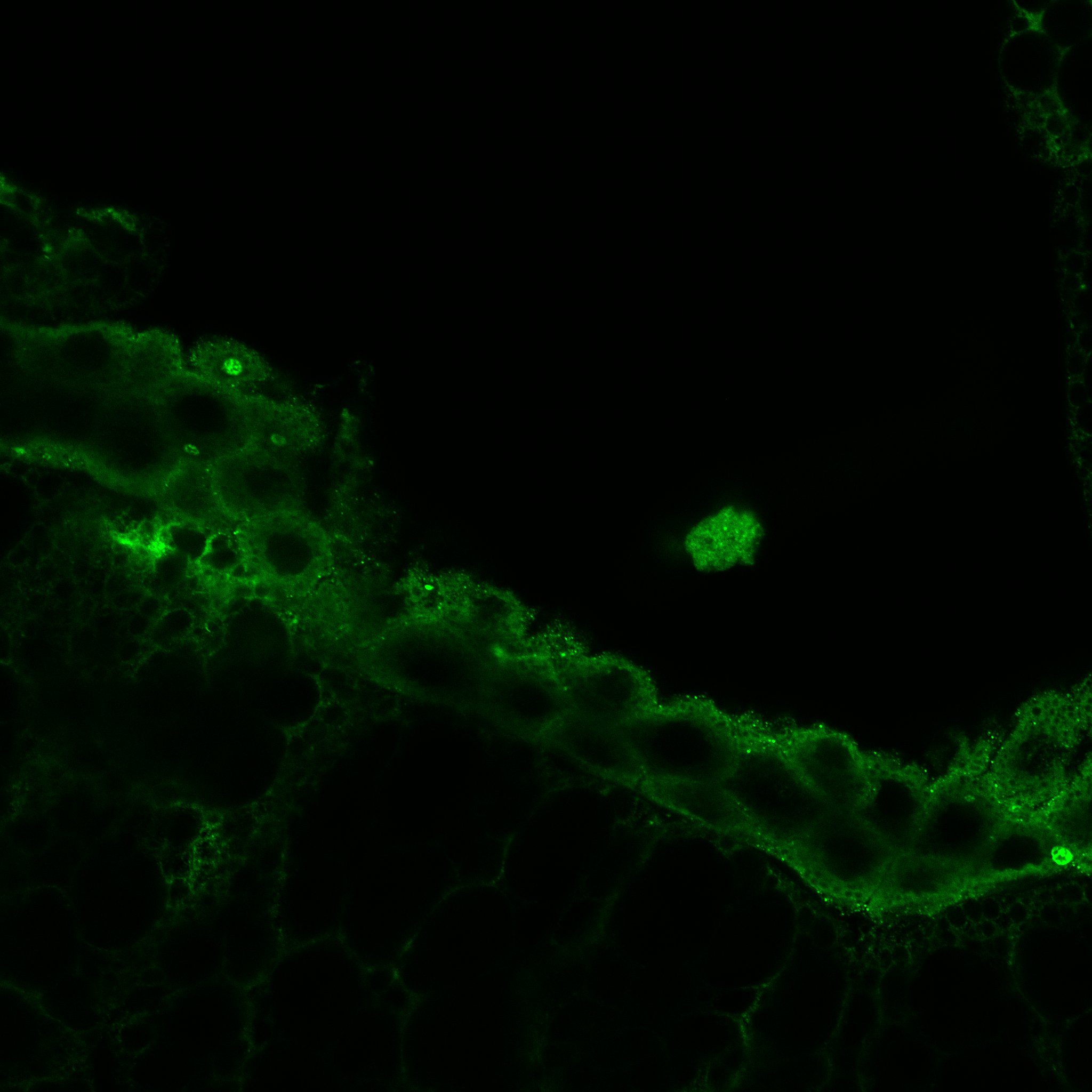

Supplement: Figure 2—figure supplement 2—source data 2. [file elife-105165-fig2-figsupp2-data2.zip › Figure 2-figure supplement 2_Source data 2/S4_B_C2.jpg]

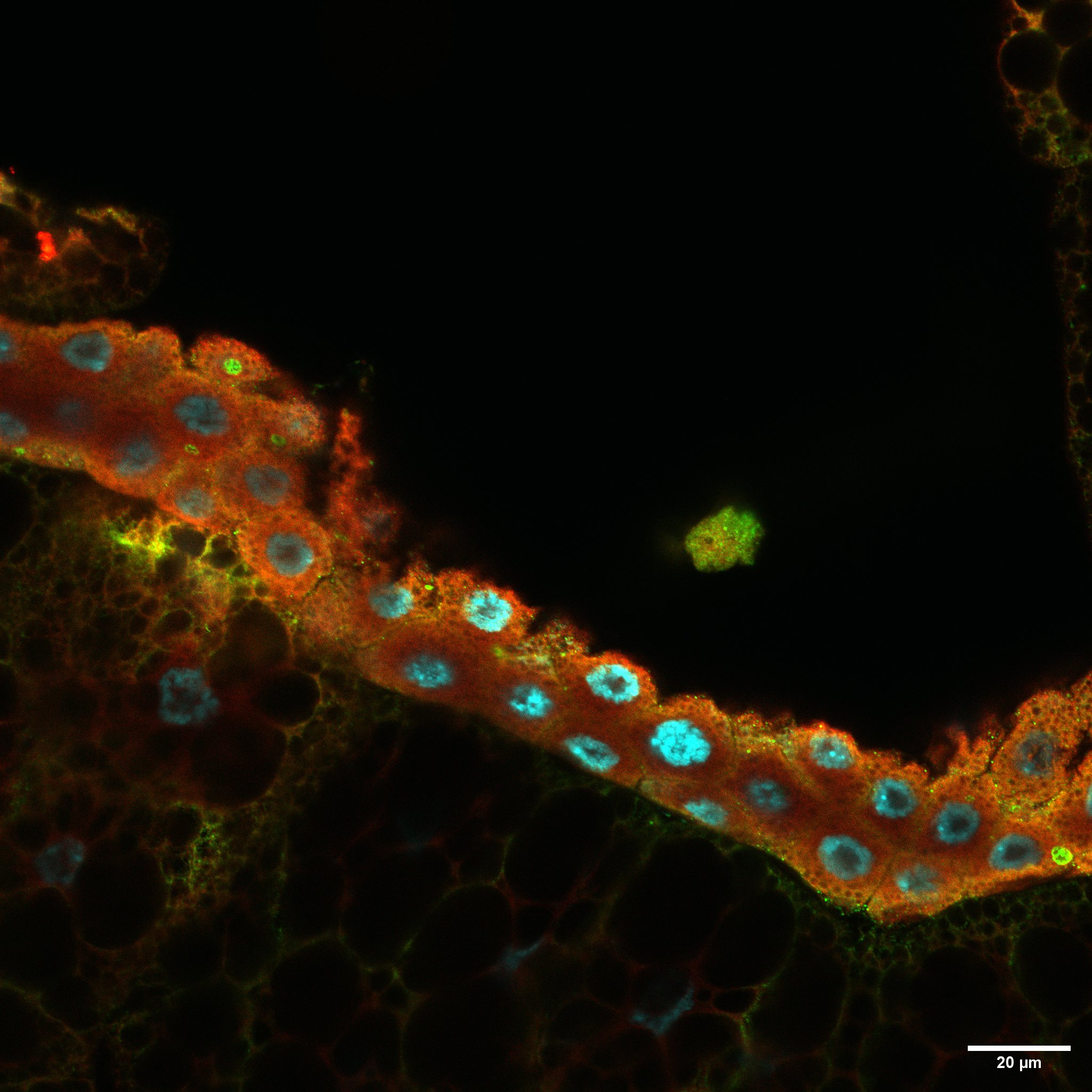

Supplement: Figure 2—figure supplement 2—source data 2. [file elife-105165-fig2-figsupp2-data2.zip › Figure 2-figure supplement 2_Source data 2/S4_B_.jpg]

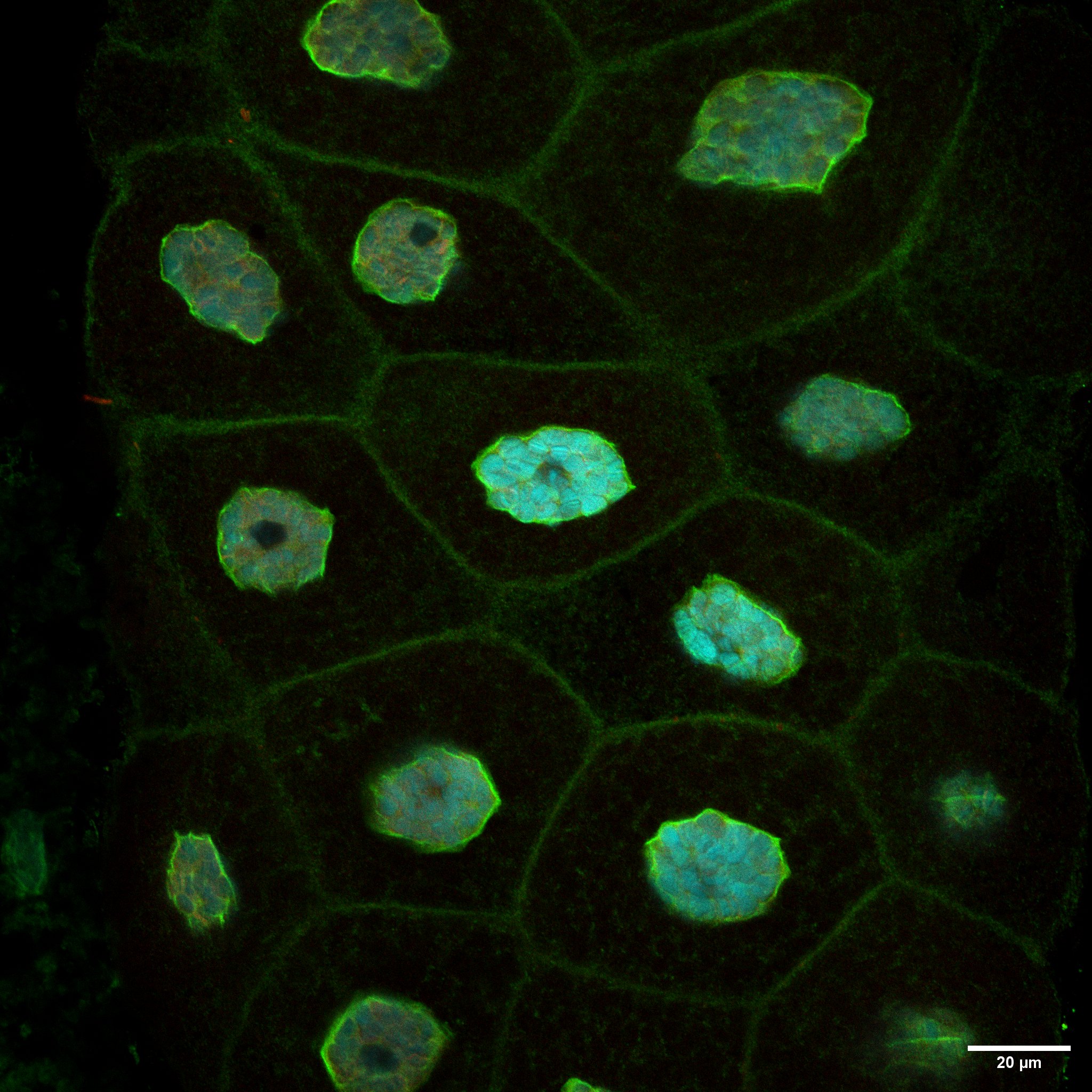

Supplement: Figure 3—source data 2. [file elife-105165-fig3-data2.zip › Figure 3 source data 2/3A_.jpg]

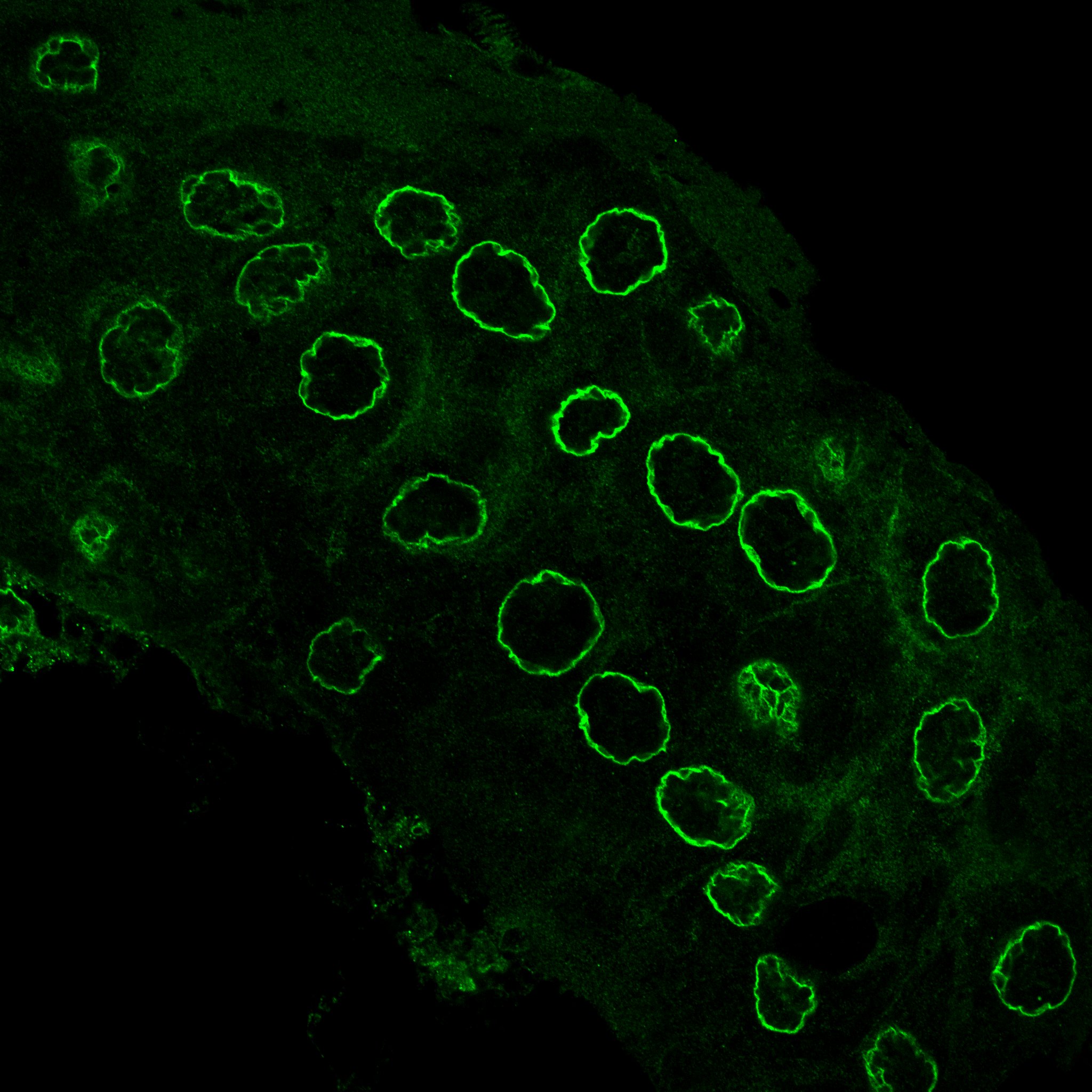

Supplement: Figure 3—source data 2. [file elife-105165-fig3-data2.zip › Figure 3 source data 2/3C_C2.jpg]

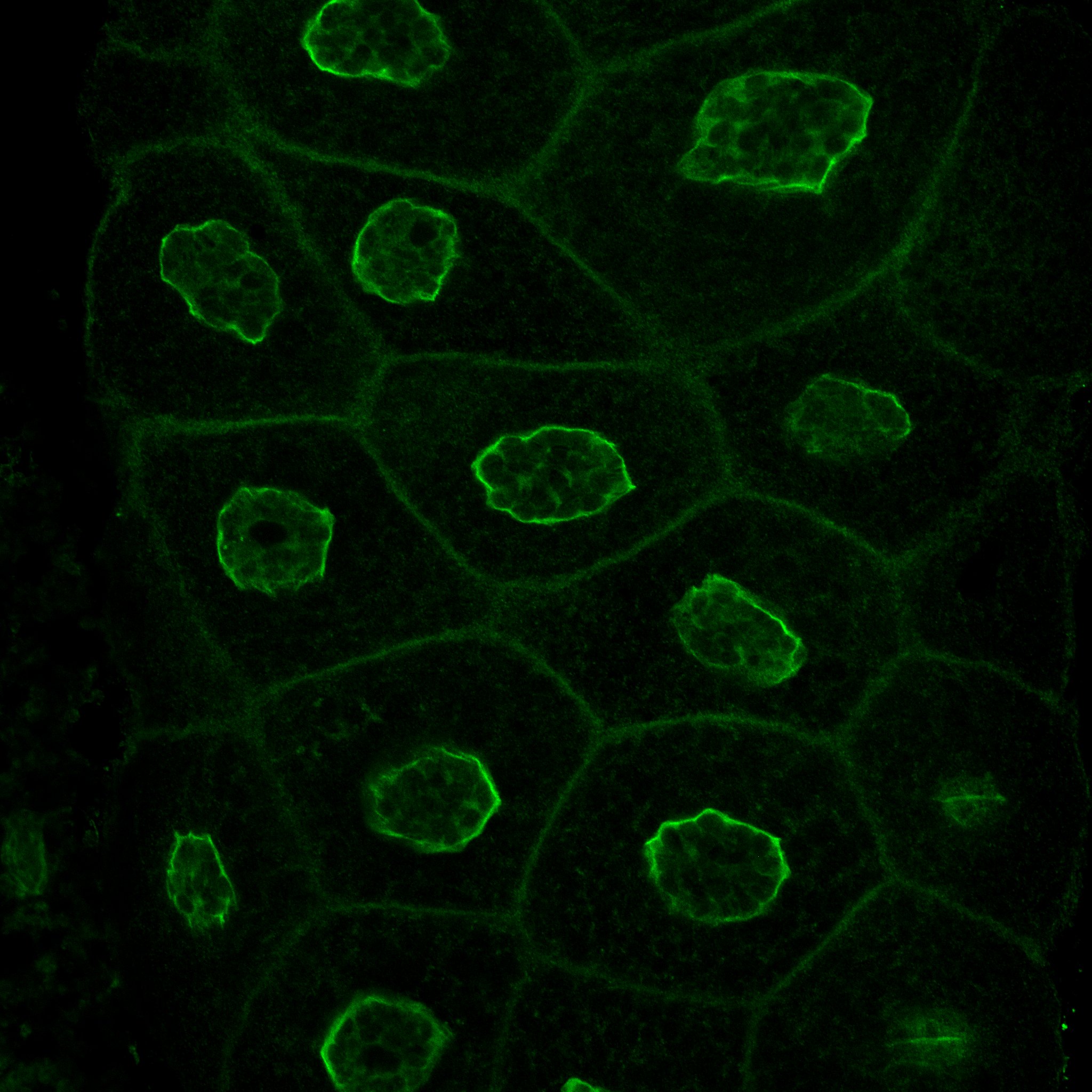

Supplement: Figure 3—source data 2. [file elife-105165-fig3-data2.zip › Figure 3 source data 2/3A_C2.jpg]

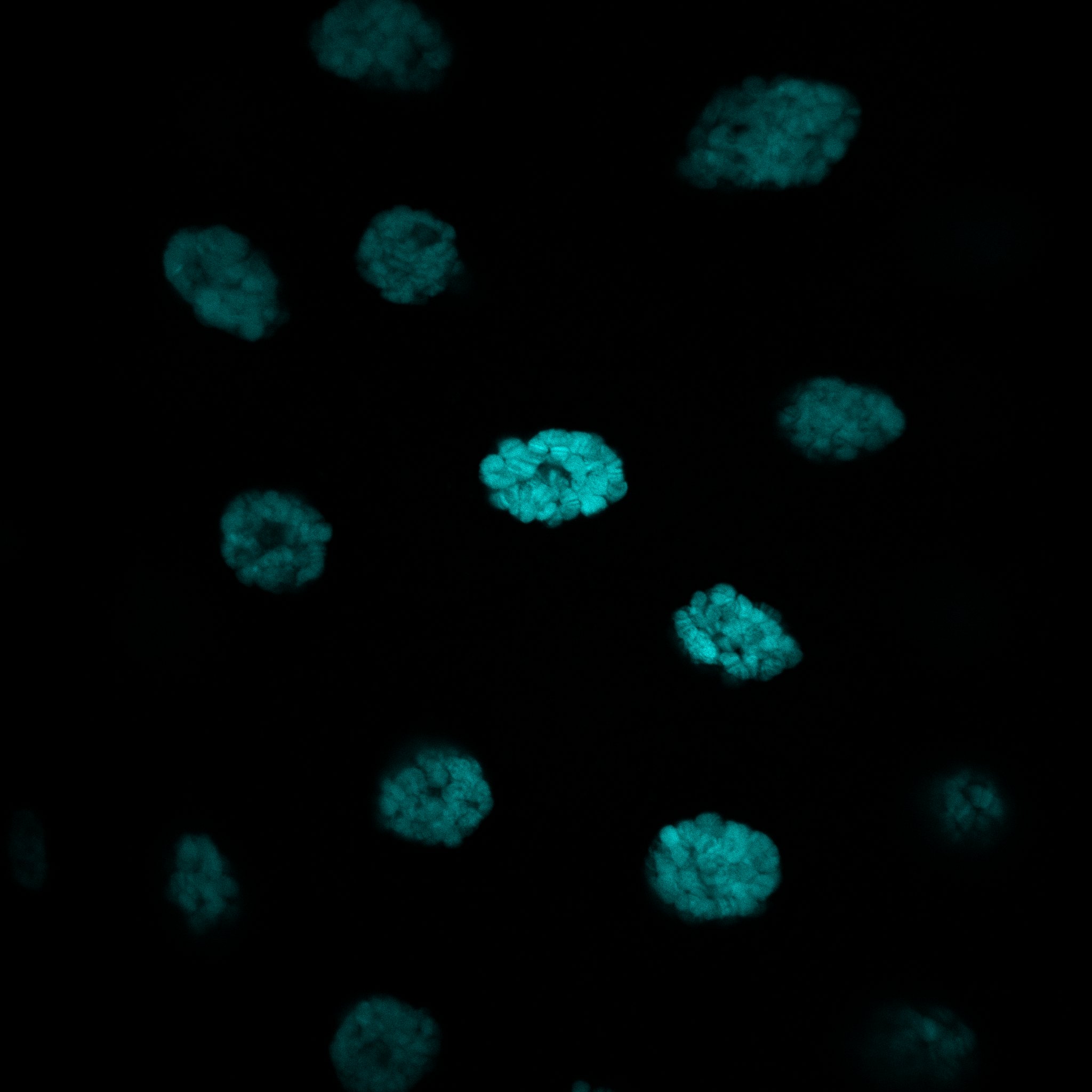

Supplement: Figure 3—source data 2. [file elife-105165-fig3-data2.zip › Figure 3 source data 2/3A_C1.jpg]

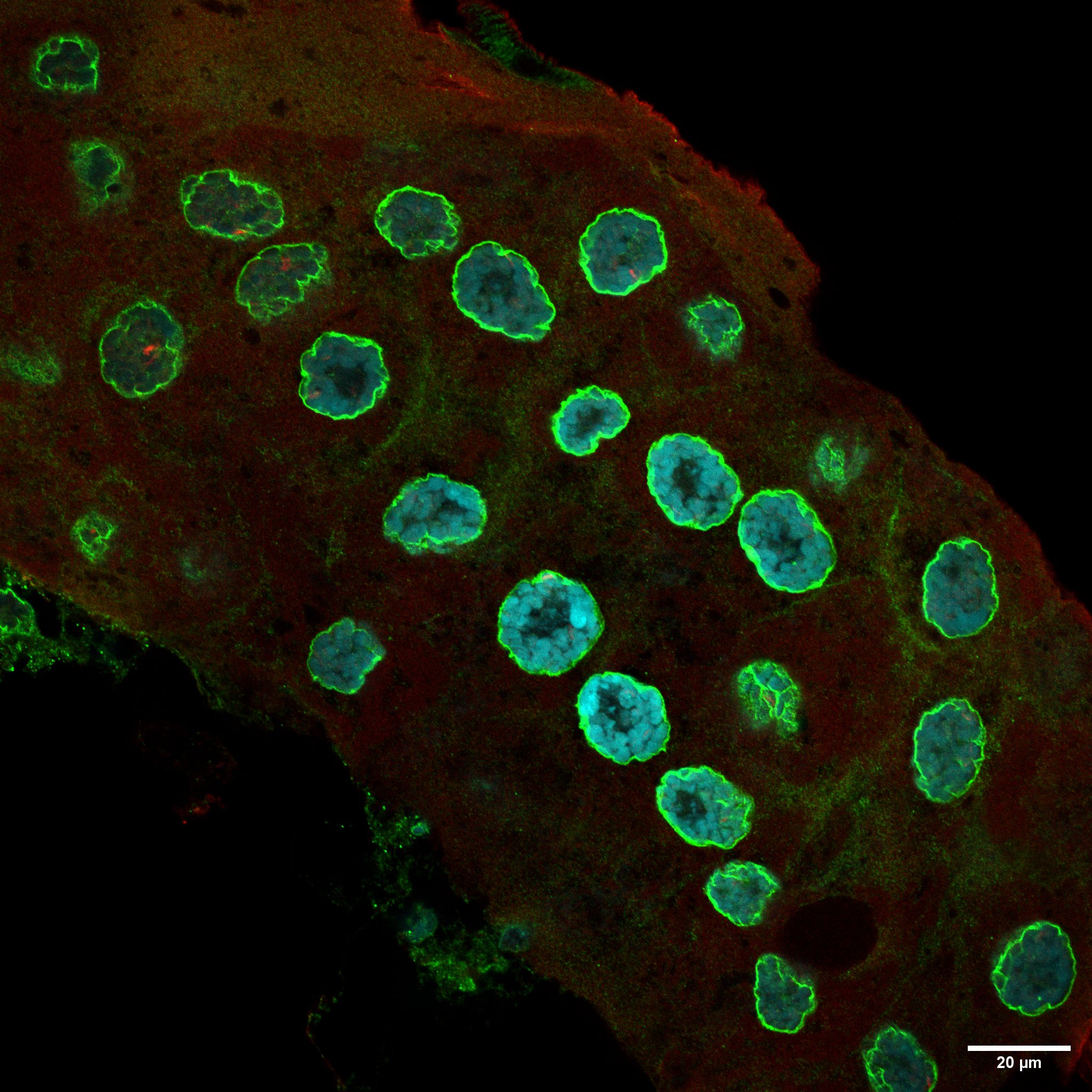

Supplement: Figure 3—source data 2. [file elife-105165-fig3-data2.zip › Figure 3 source data 2/3C_.jpg]

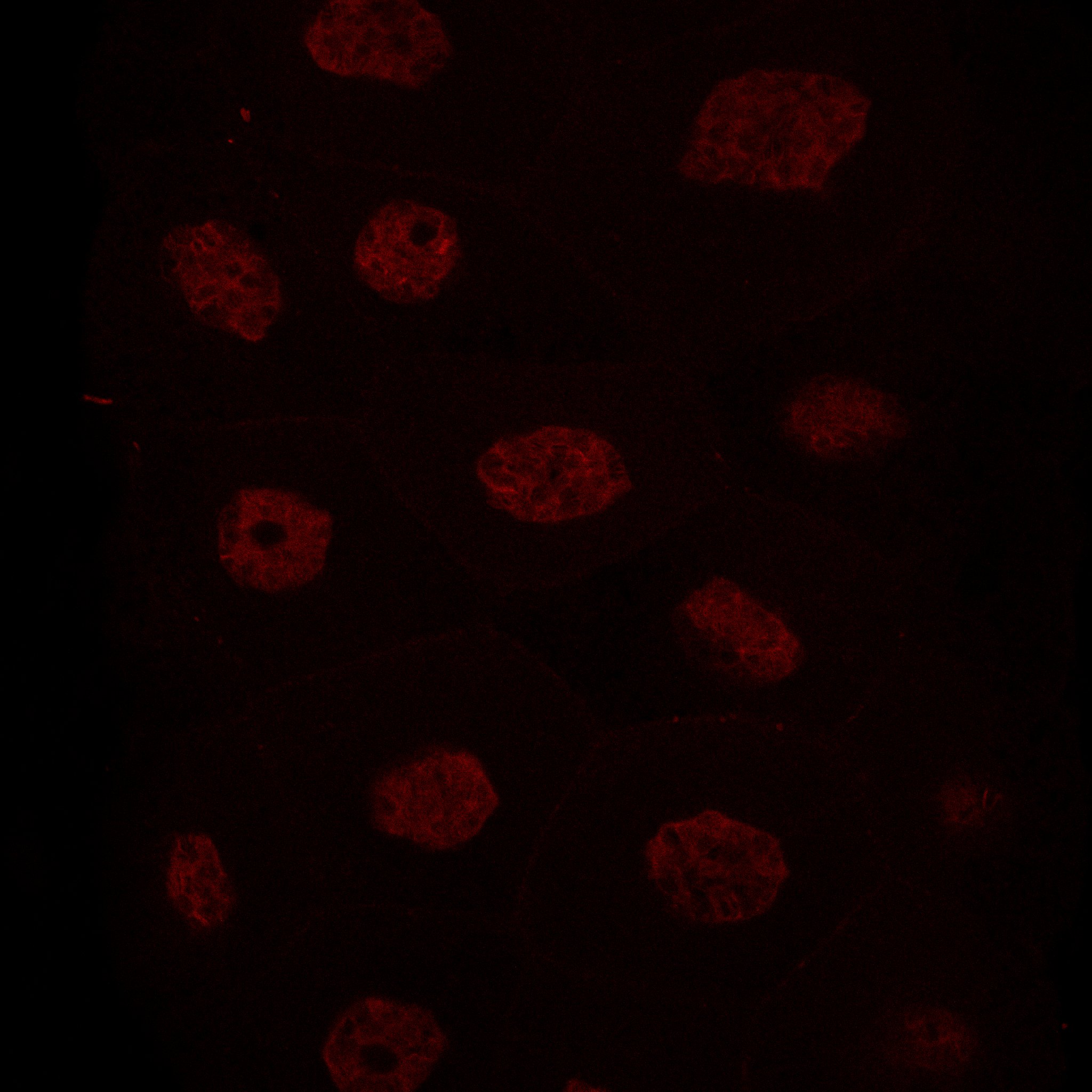

Supplement: Figure 3—source data 2. [file elife-105165-fig3-data2.zip › Figure 3 source data 2/3A_C3.jpg]

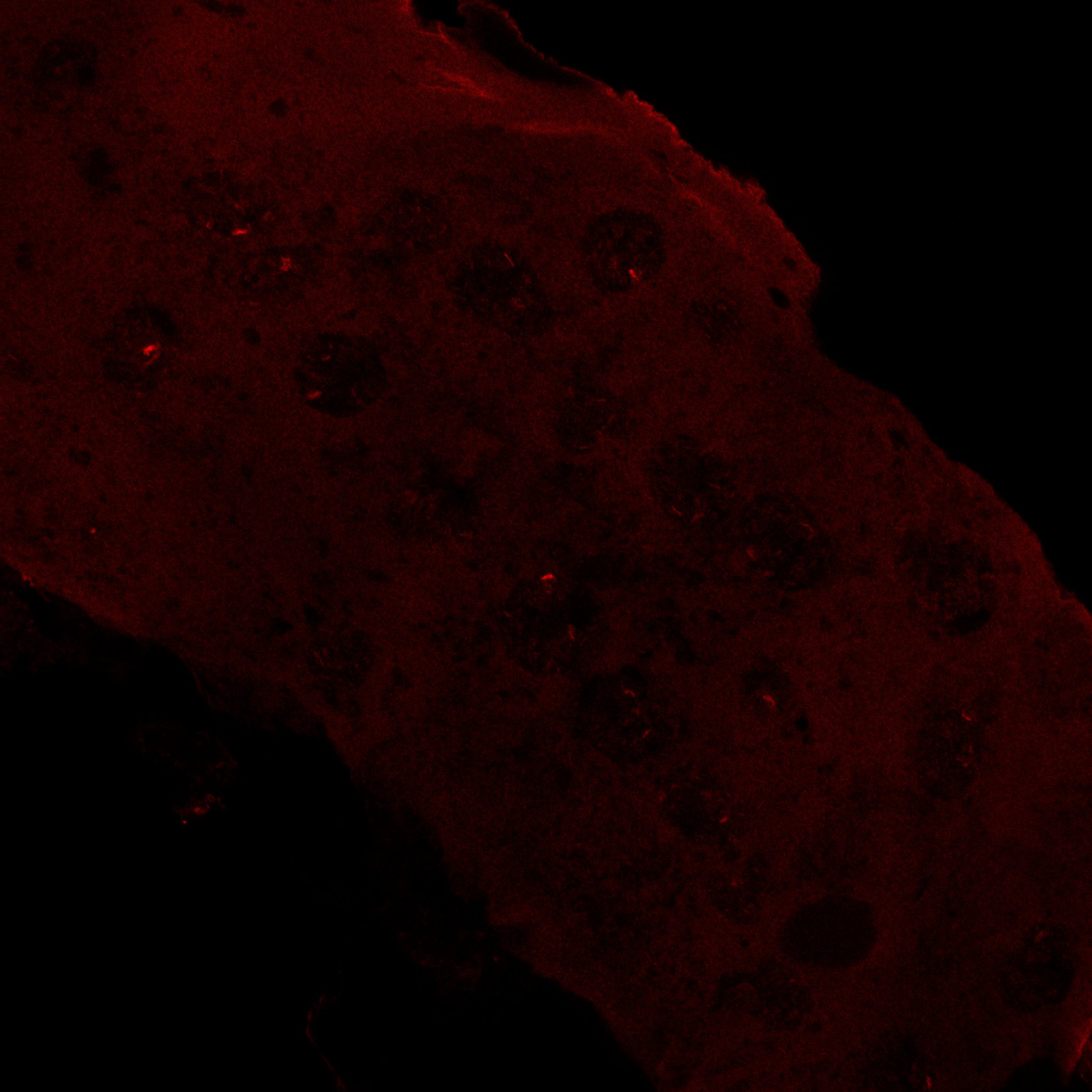

Supplement: Figure 3—source data 2. [file elife-105165-fig3-data2.zip › Figure 3 source data 2/3C_C3.jpg]

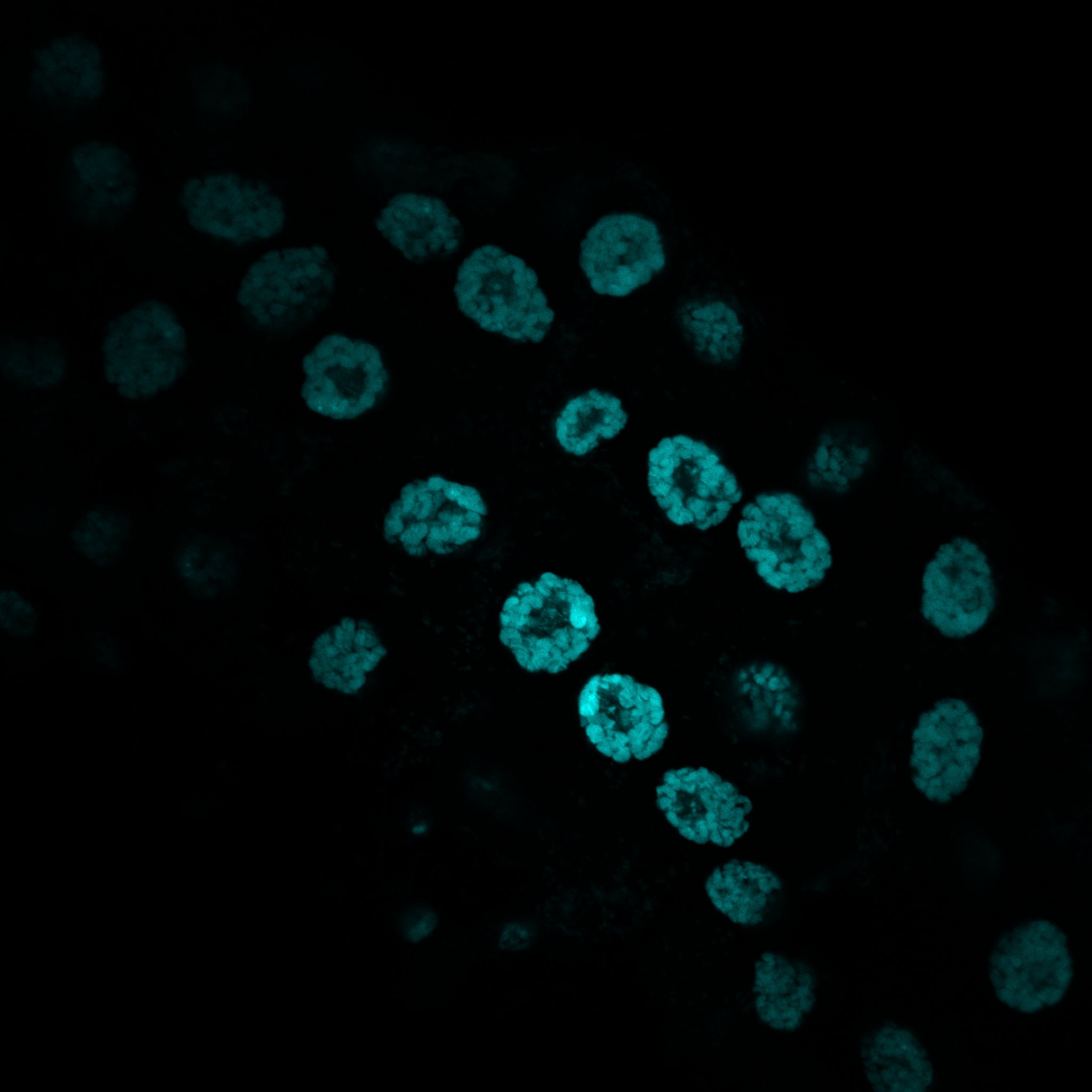

Supplement: Figure 3—source data 2. [file elife-105165-fig3-data2.zip › Figure 3 source data 2/3C_C1.jpg]

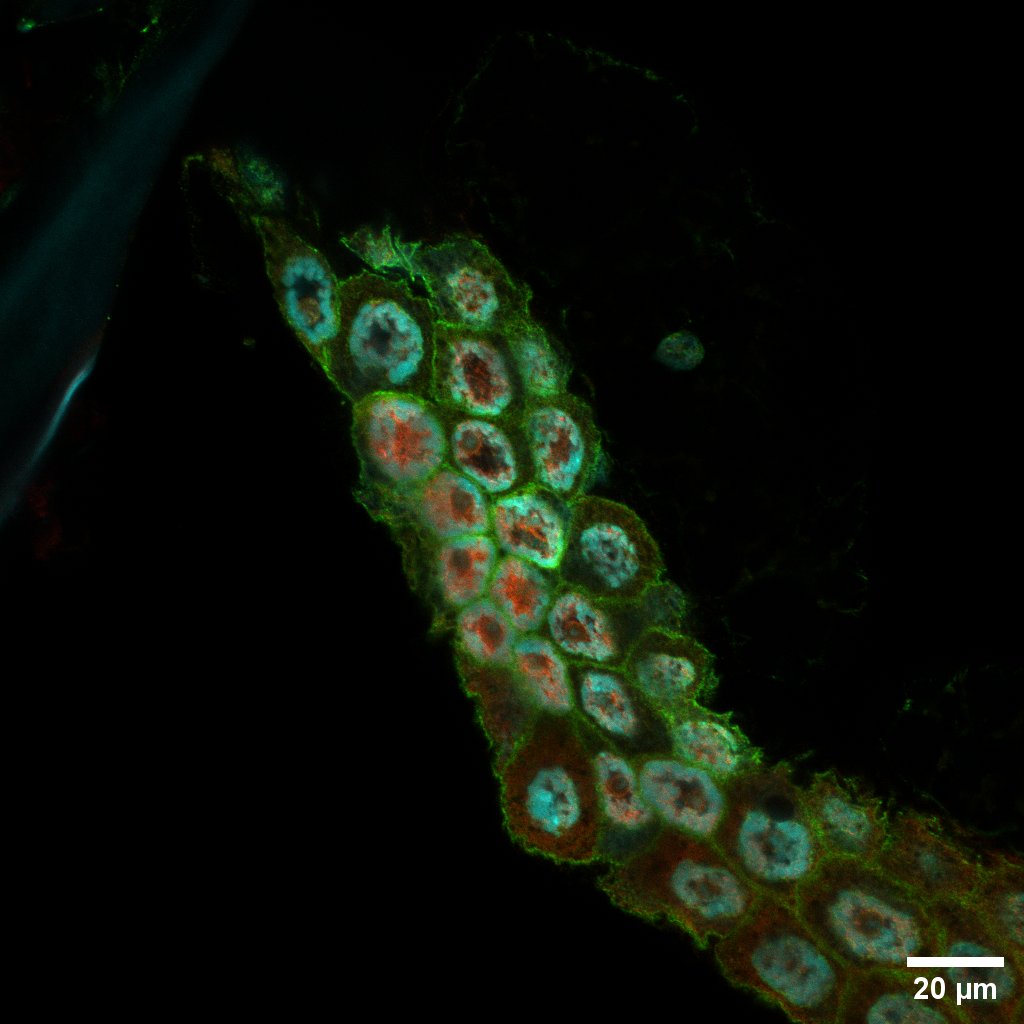

Supplement: Figure 3—source data 2. [file elife-105165-fig3-data2.zip › Figure 3 source data 2/3B_.jpg]

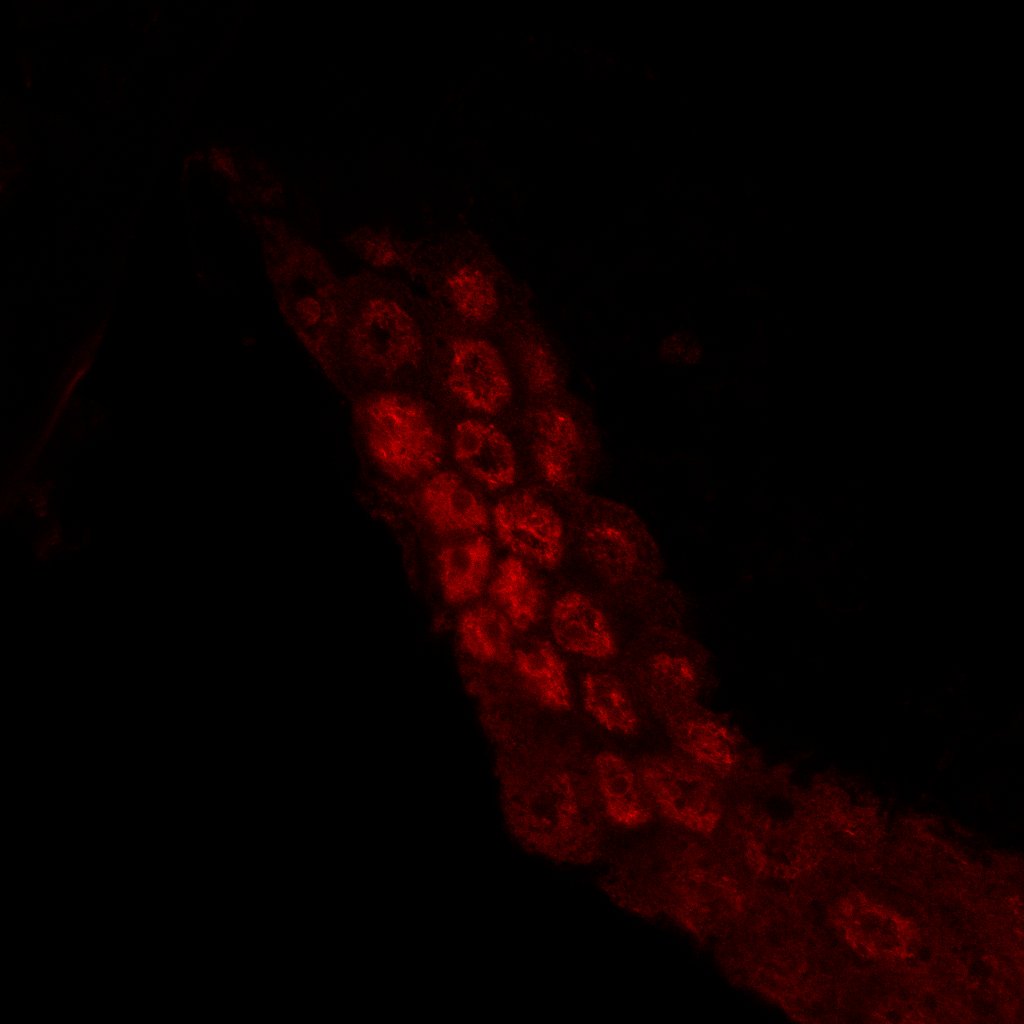

Supplement: Figure 3—source data 2. [file elife-105165-fig3-data2.zip › Figure 3 source data 2/3B_C3.jpg]

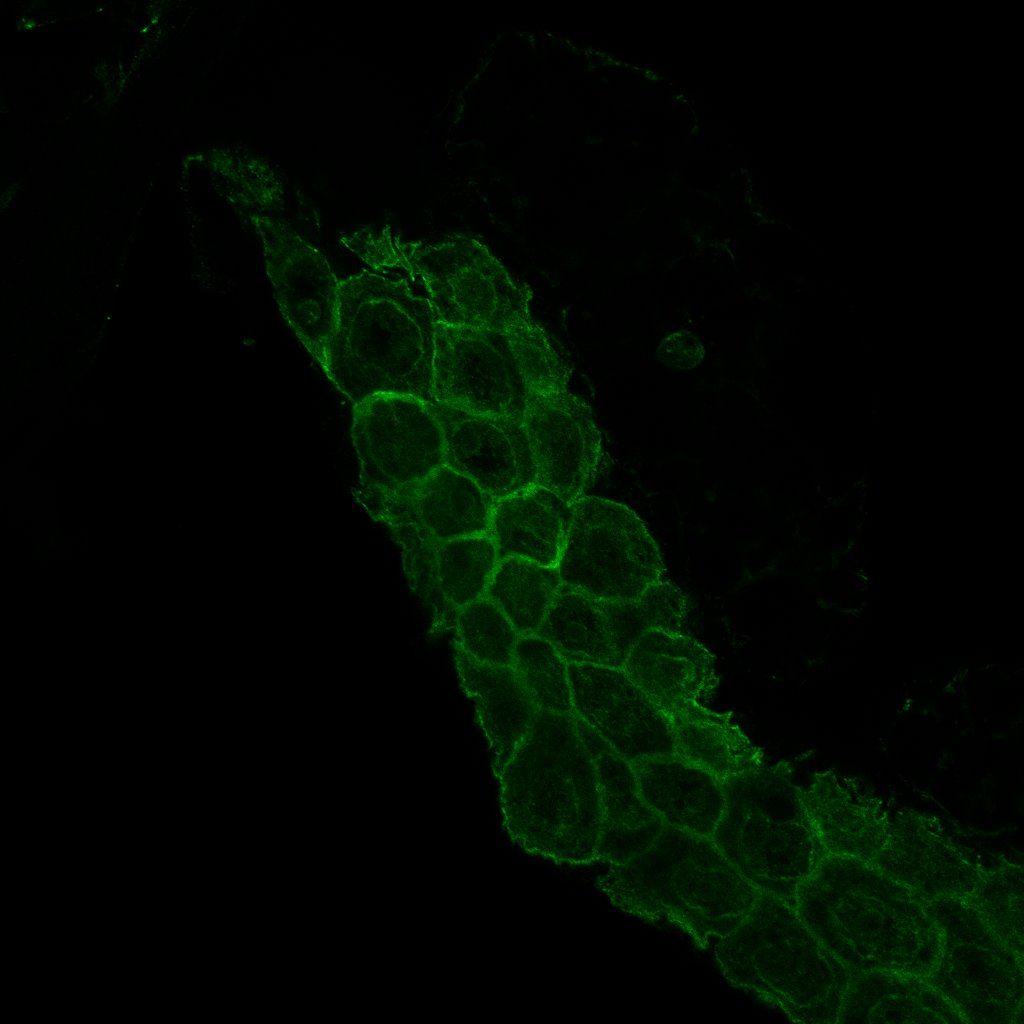

Supplement: Figure 3—source data 2. [file elife-105165-fig3-data2.zip › Figure 3 source data 2/3B_C2.jpg]

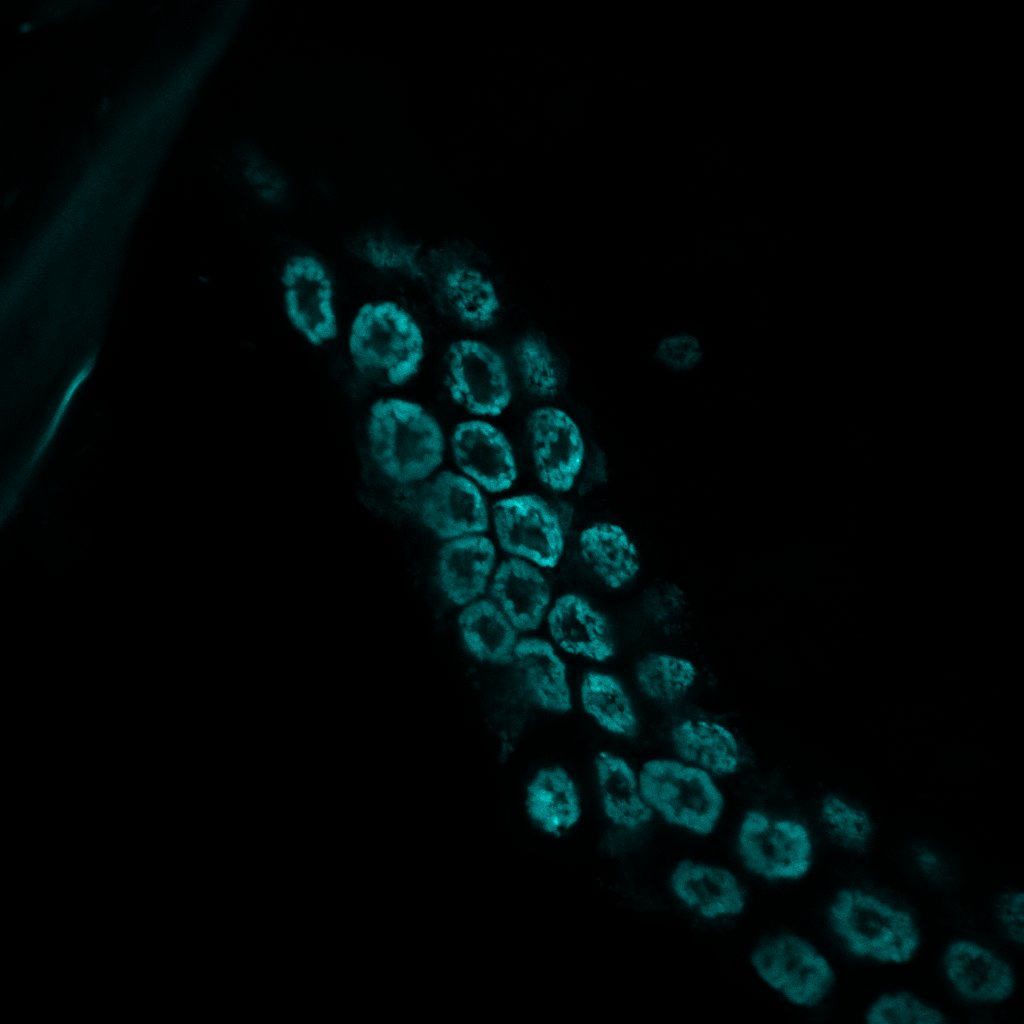

Supplement: Figure 3—source data 2. [file elife-105165-fig3-data2.zip › Figure 3 source data 2/3B_C1.jpg]

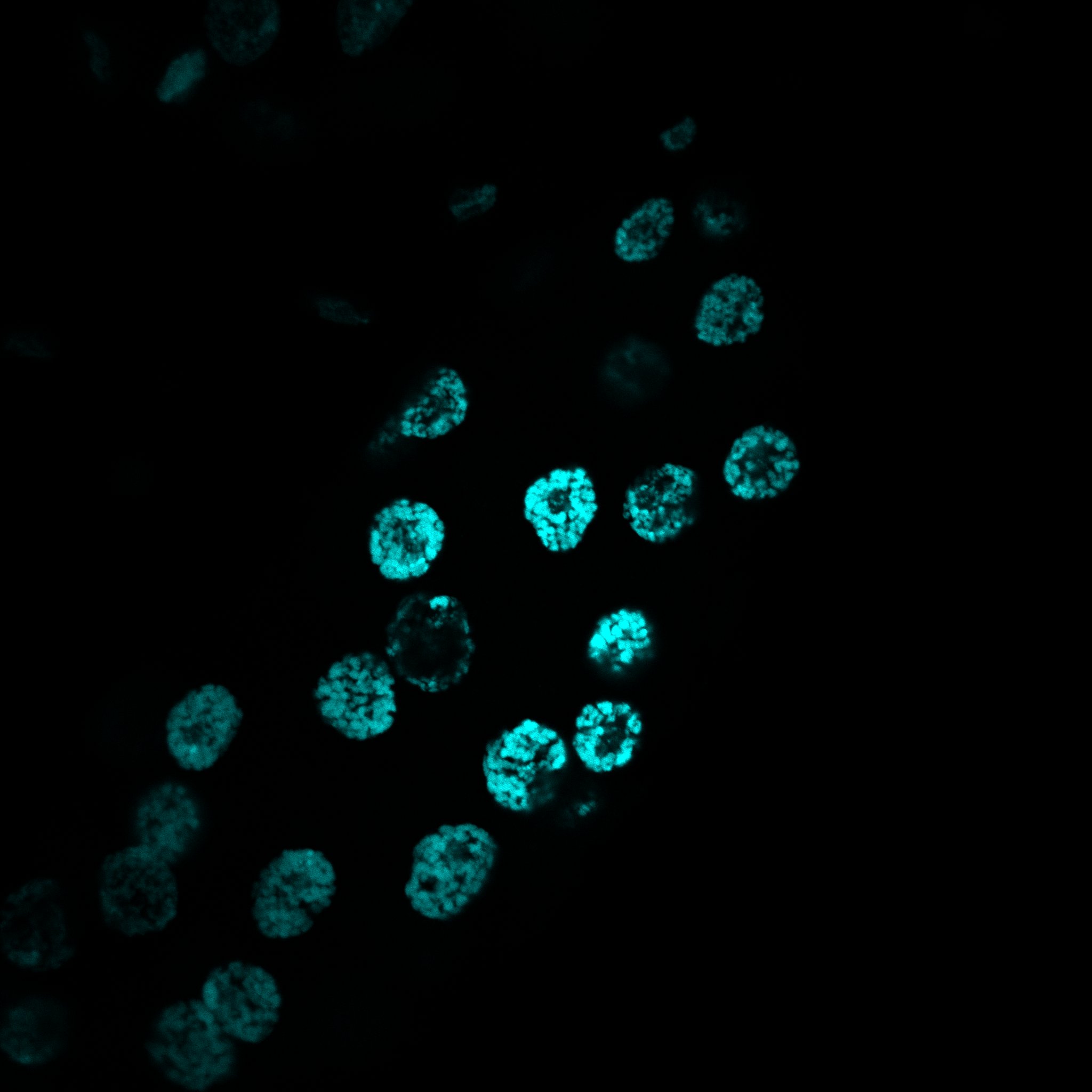

Supplement: Figure 3—figure supplement 1—source data 2. [file elife-105165-fig3-figsupp1-data2.zip › Figure 3-figure supplement 1 Source data 2/S5_C_C1.jpg]

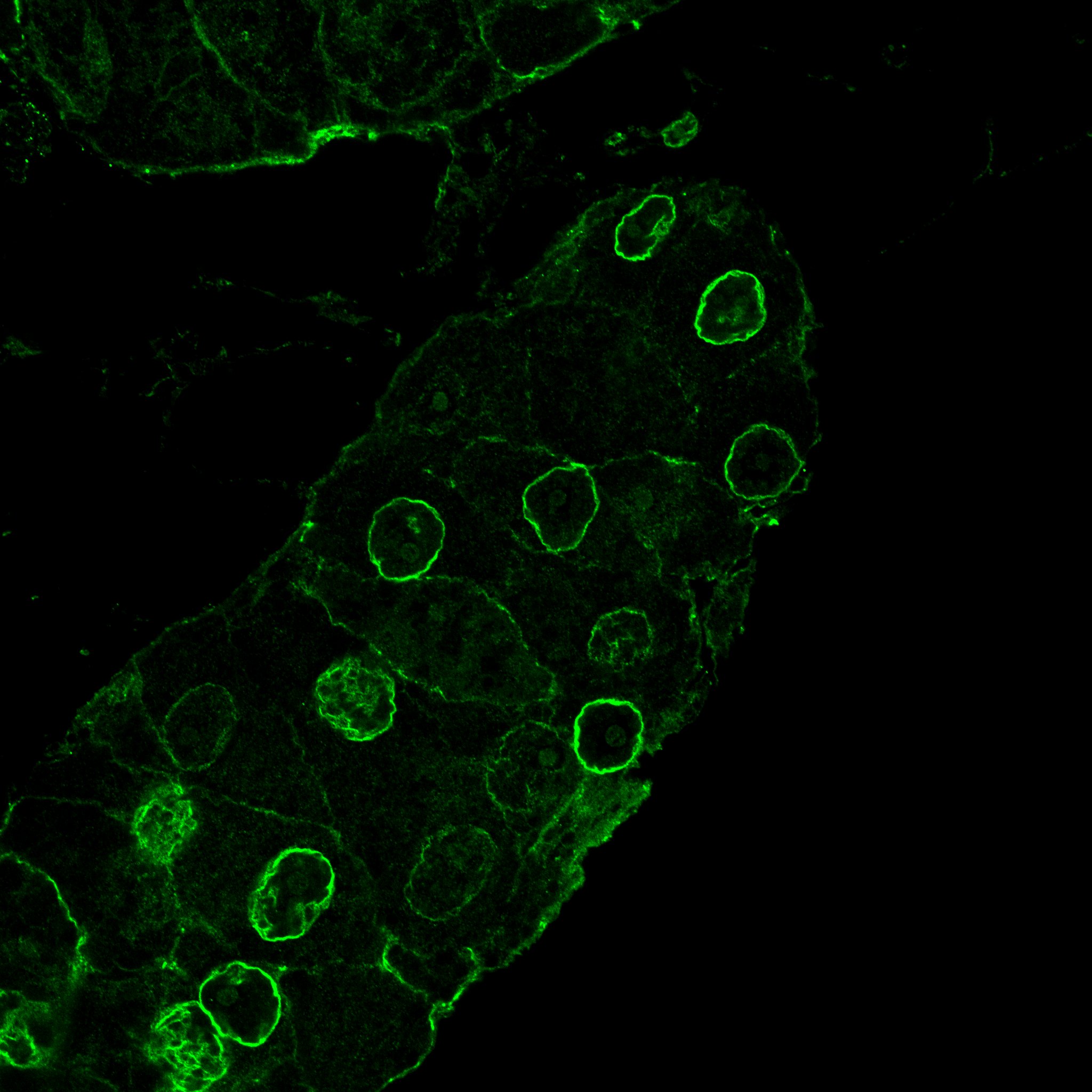

Supplement: Figure 3—figure supplement 1—source data 2. [file elife-105165-fig3-figsupp1-data2.zip › Figure 3-figure supplement 1 Source data 2/S5_C_C2.jpg]

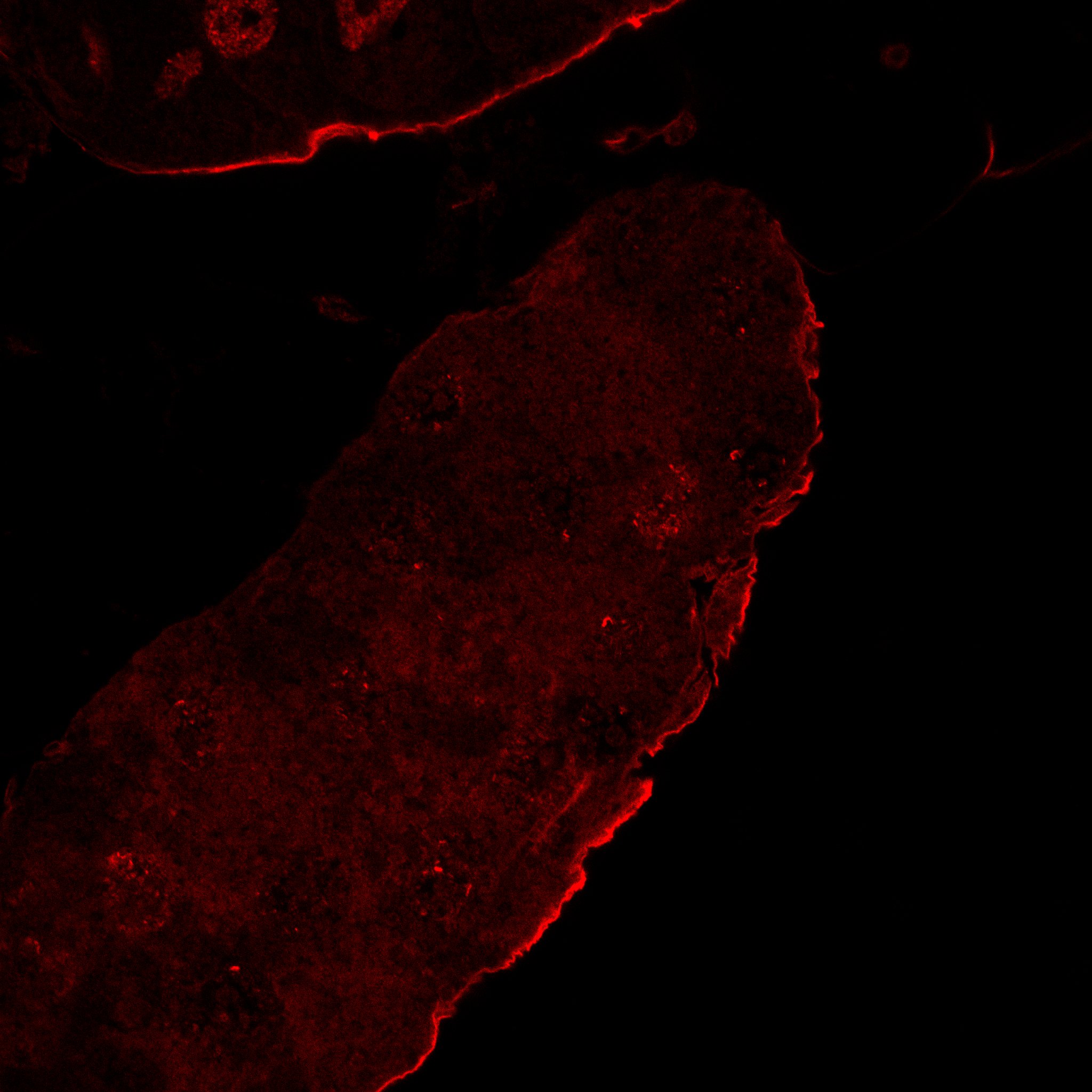

Supplement: Figure 3—figure supplement 1—source data 2. [file elife-105165-fig3-figsupp1-data2.zip › Figure 3-figure supplement 1 Source data 2/S5_C_C3.jpg]

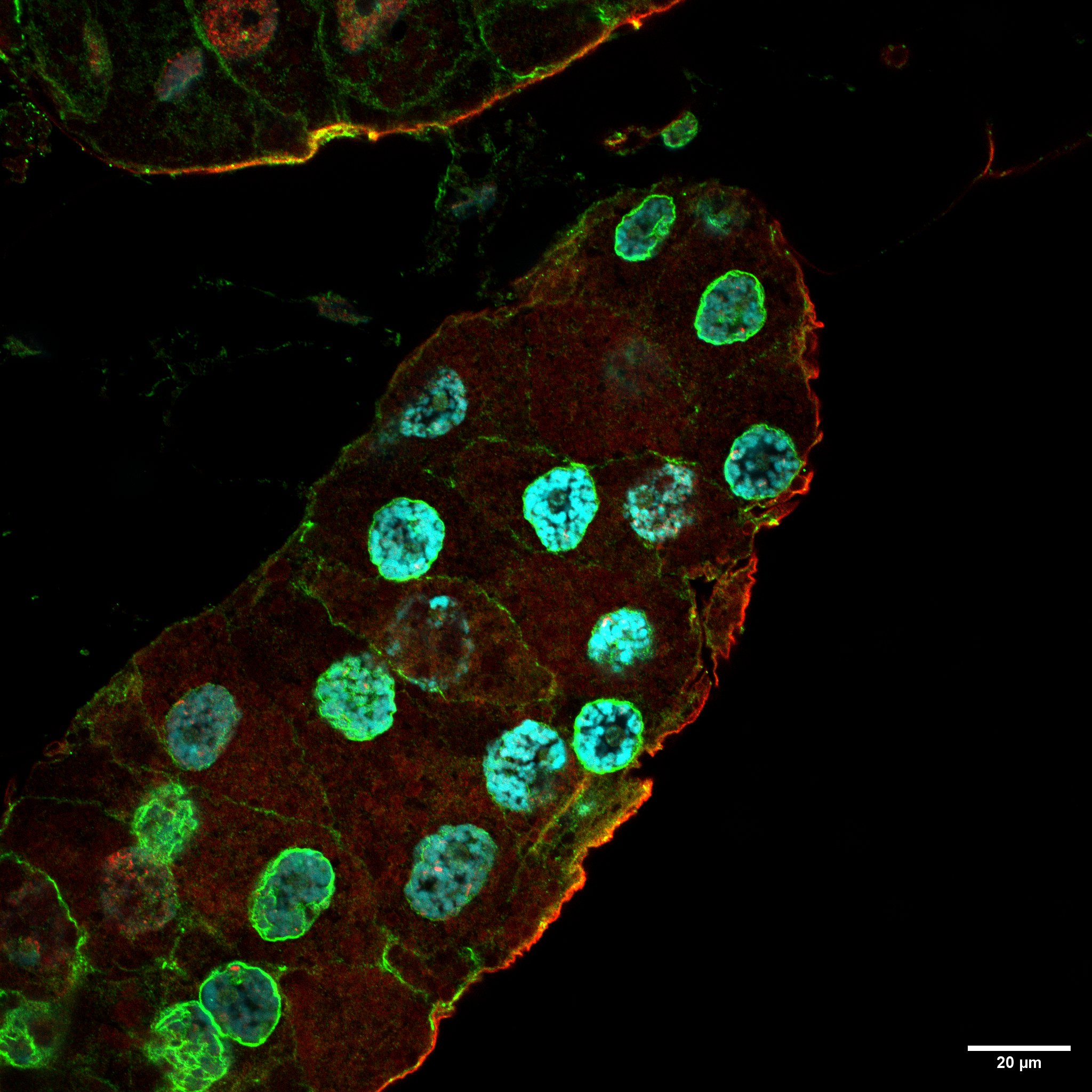

Supplement: Figure 3—figure supplement 1—source data 2. [file elife-105165-fig3-figsupp1-data2.zip › Figure 3-figure supplement 1 Source data 2/S5_C.jpg]

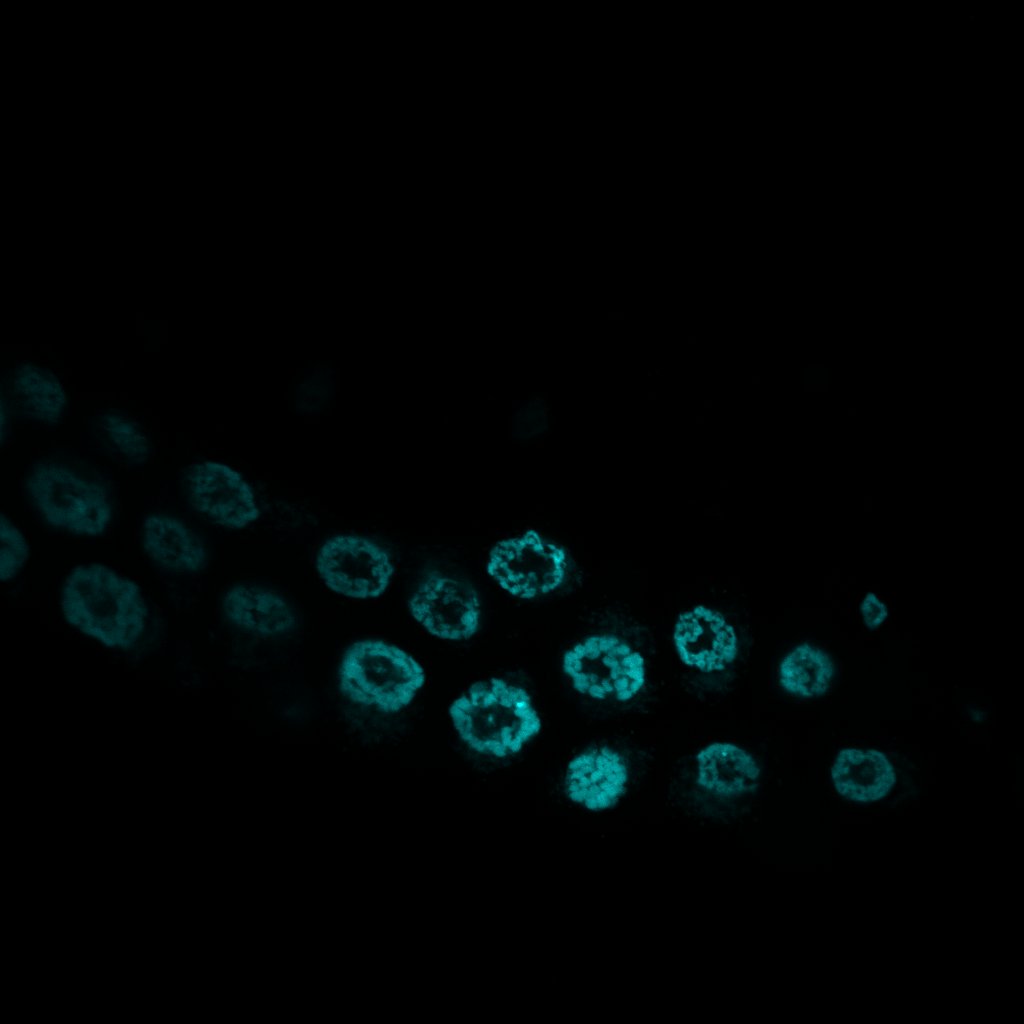

Supplement: Figure 3—figure supplement 1—source data 2. [file elife-105165-fig3-figsupp1-data2.zip › Figure 3-figure supplement 1 Source data 2/S5_B_C1.jpg]

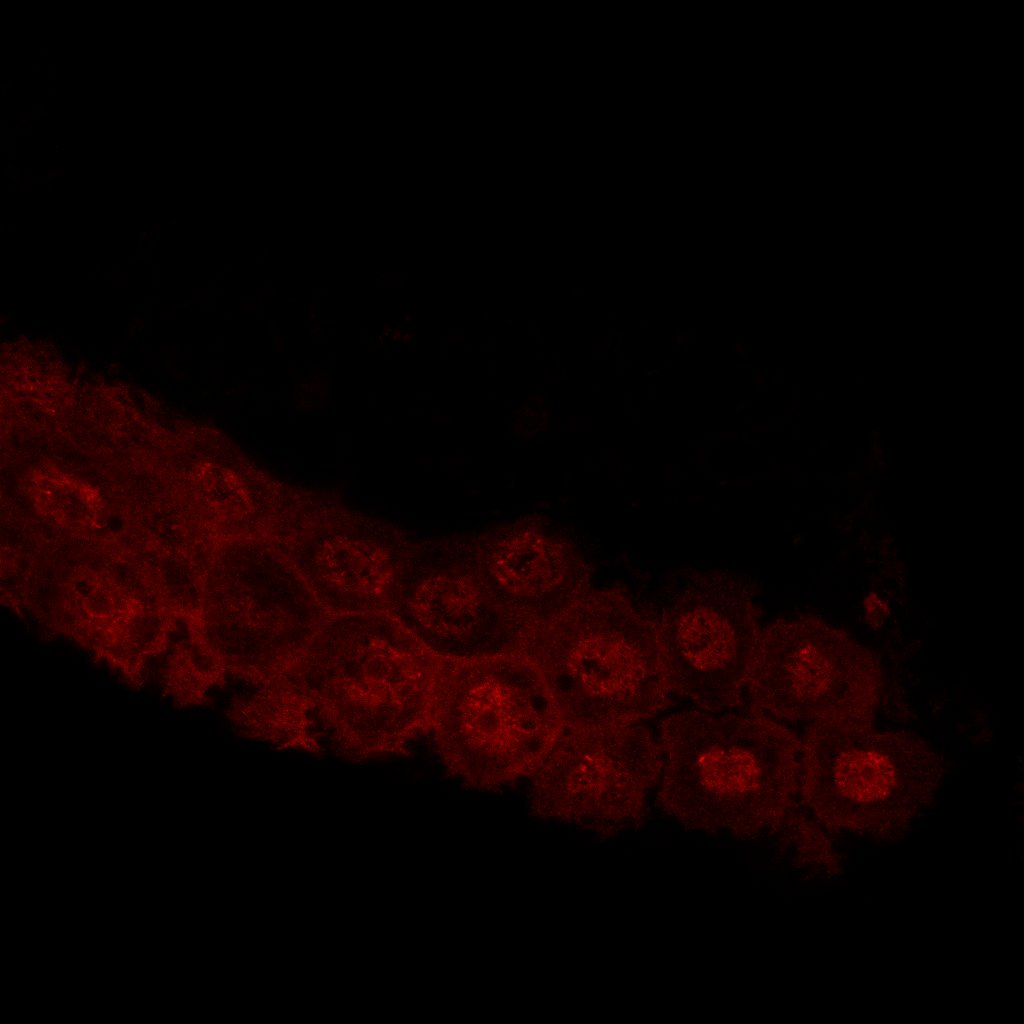

Supplement: Figure 3—figure supplement 1—source data 2. [file elife-105165-fig3-figsupp1-data2.zip › Figure 3-figure supplement 1 Source data 2/S5_B_C3.jpg]

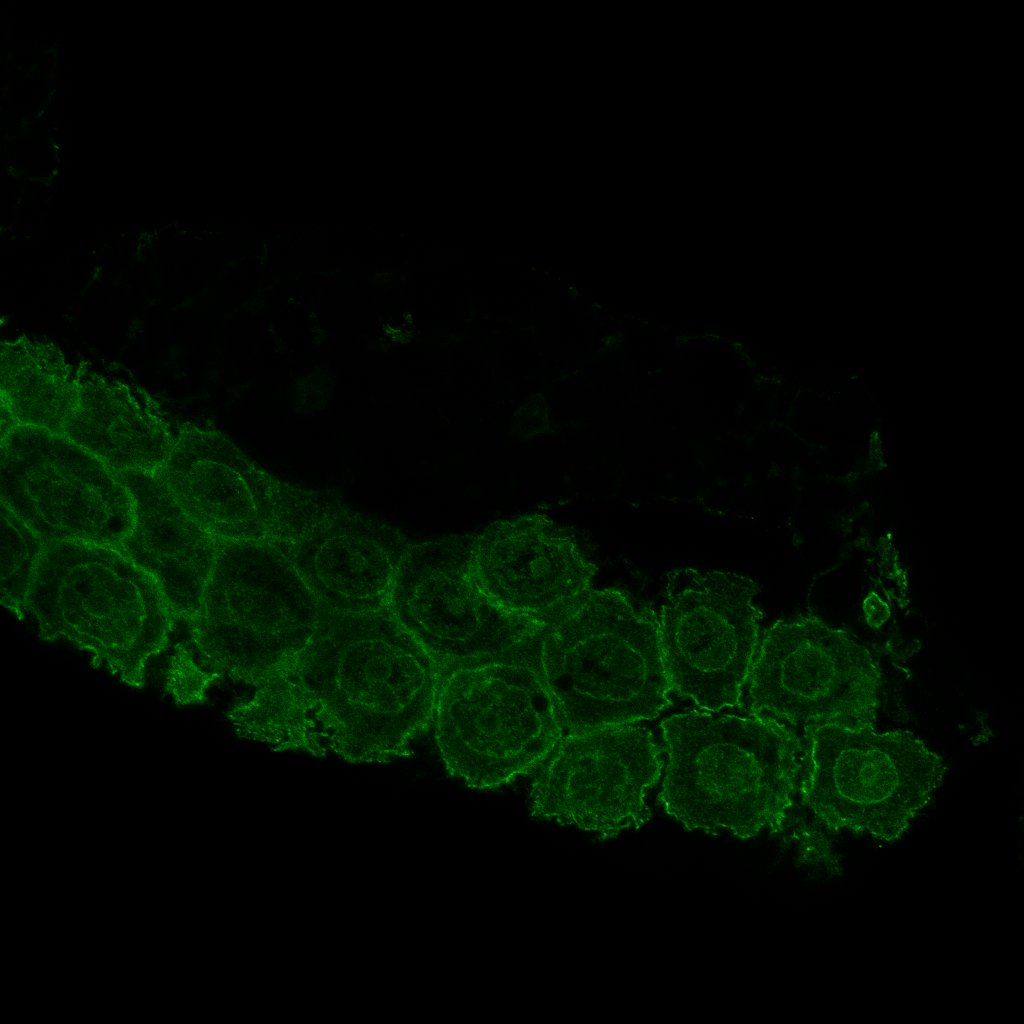

Supplement: Figure 3—figure supplement 1—source data 2. [file elife-105165-fig3-figsupp1-data2.zip › Figure 3-figure supplement 1 Source data 2/S5_B_C2.jpg]

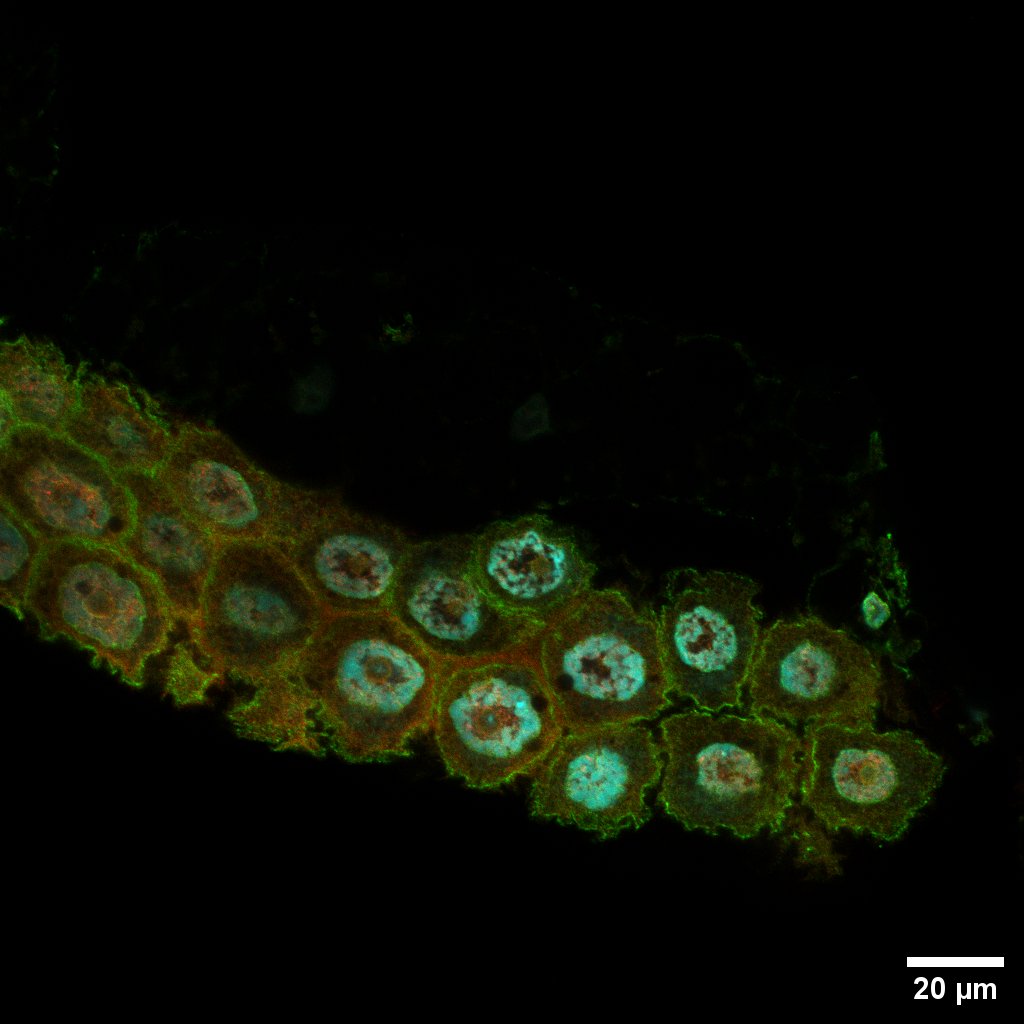

Supplement: Figure 3—figure supplement 1—source data 2. [file elife-105165-fig3-figsupp1-data2.zip › Figure 3-figure supplement 1 Source data 2/S5_B.jpg]

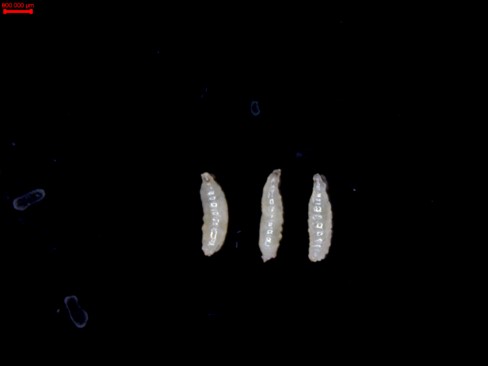

Supplement: Figure 3—figure supplement 2—source data 2. [file elife-105165-fig3-figsupp2-data2.zip › Figure 3-figure supplement 2 source data 2/AB1_01.jpg]

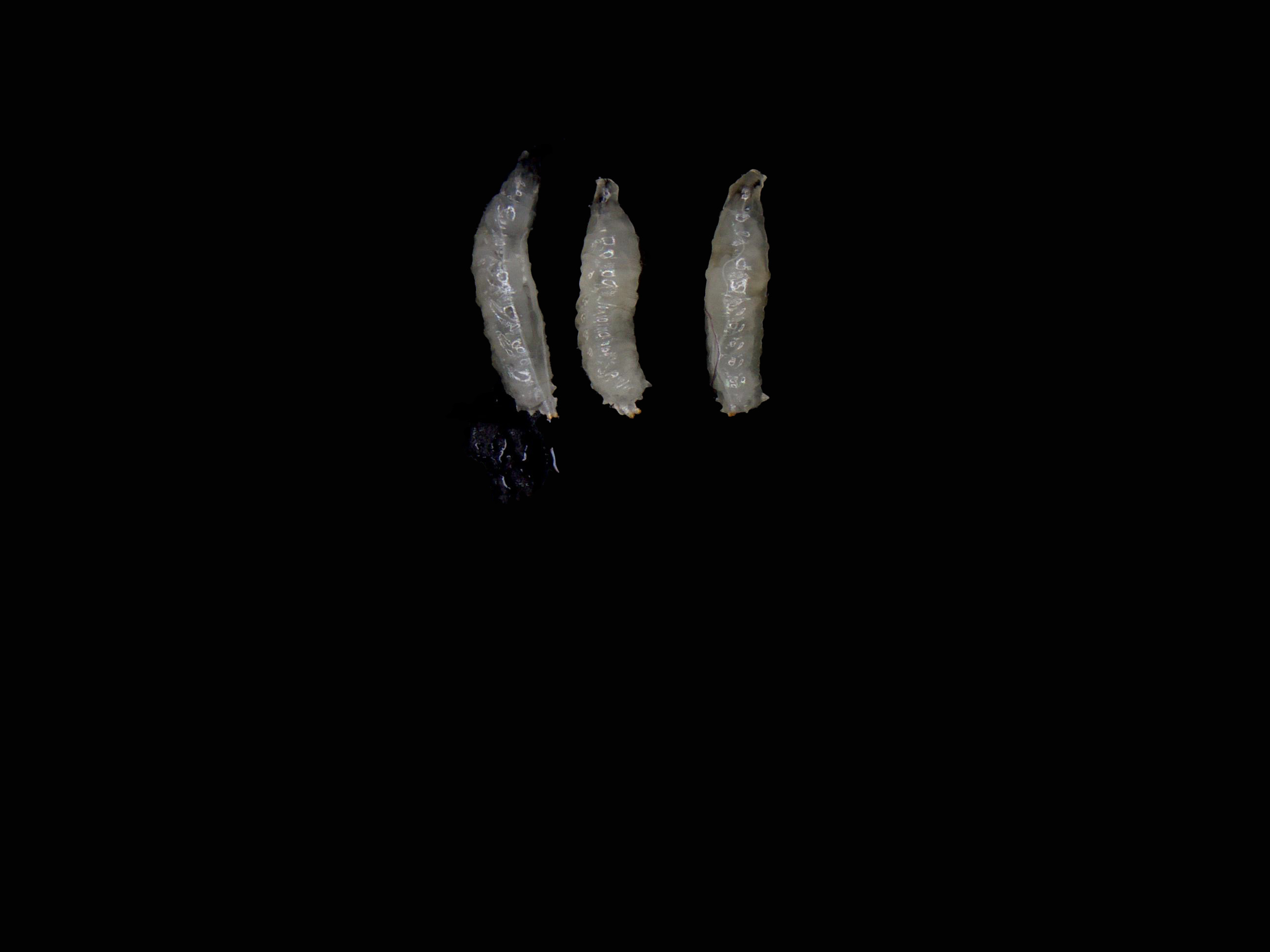

Supplement: Figure 3—figure supplement 2—source data 2. [file elife-105165-fig3-figsupp2-data2.zip › Figure 3-figure supplement 2 source data 2/phm.jpg]

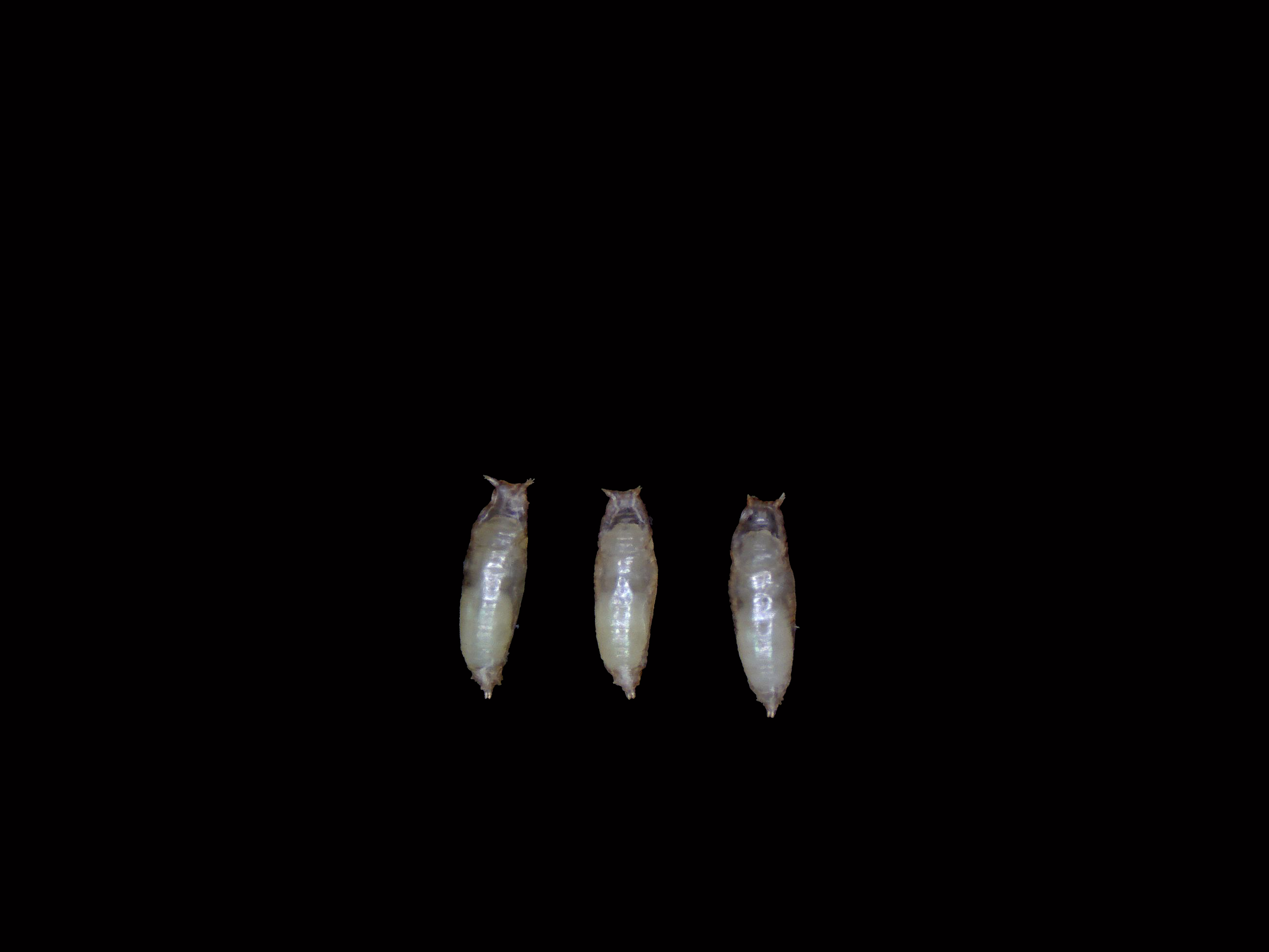

Supplement: Figure 3—figure supplement 2—source data 2. [file elife-105165-fig3-figsupp2-data2.zip › Figure 3-figure supplement 2 source data 2/AB1.jpg]

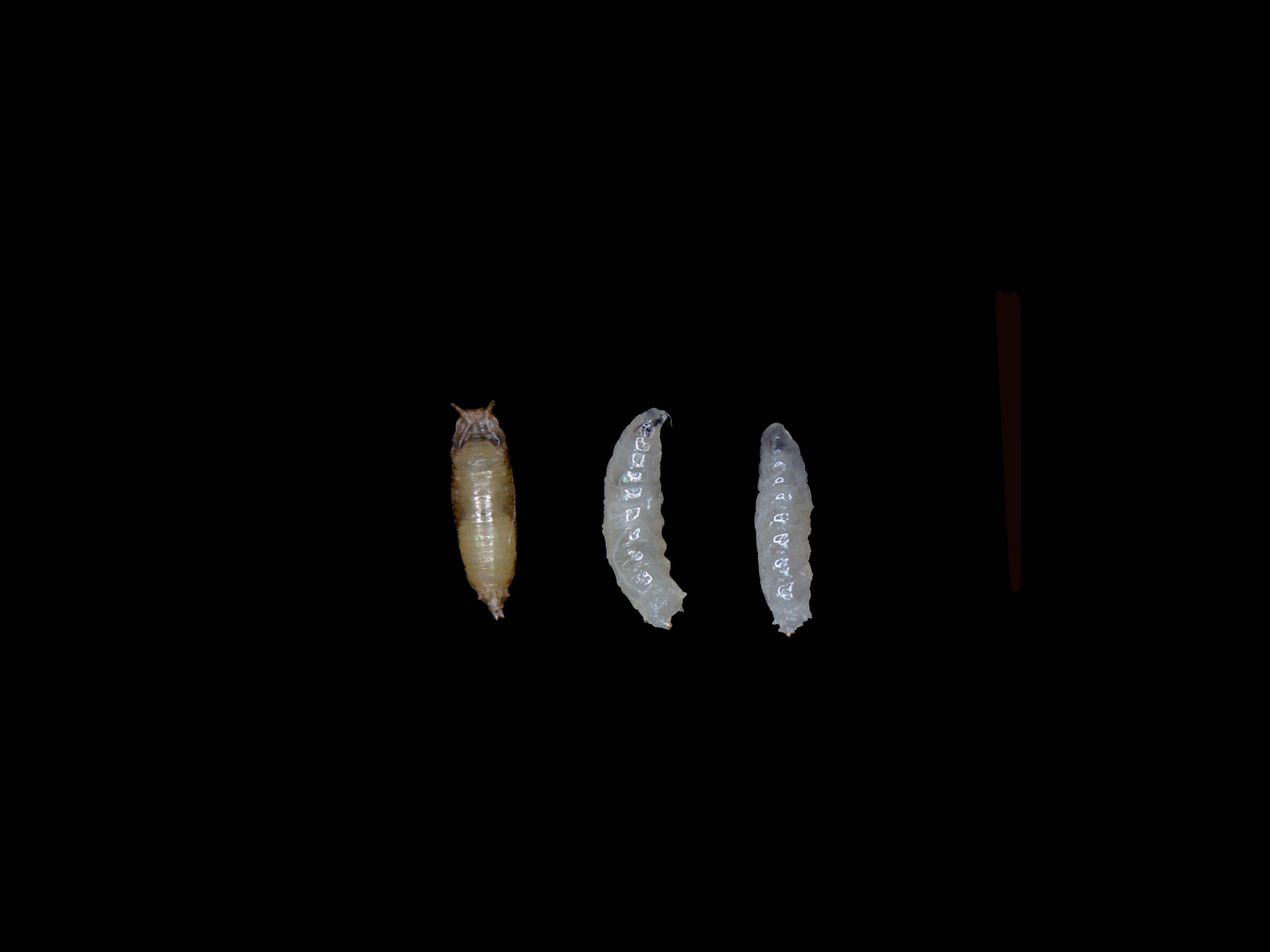

Supplement: Figure 3—figure supplement 2—source data 2. [file elife-105165-fig3-figsupp2-data2.zip › Figure 3-figure supplement 2 source data 2/phm_01.jpg]

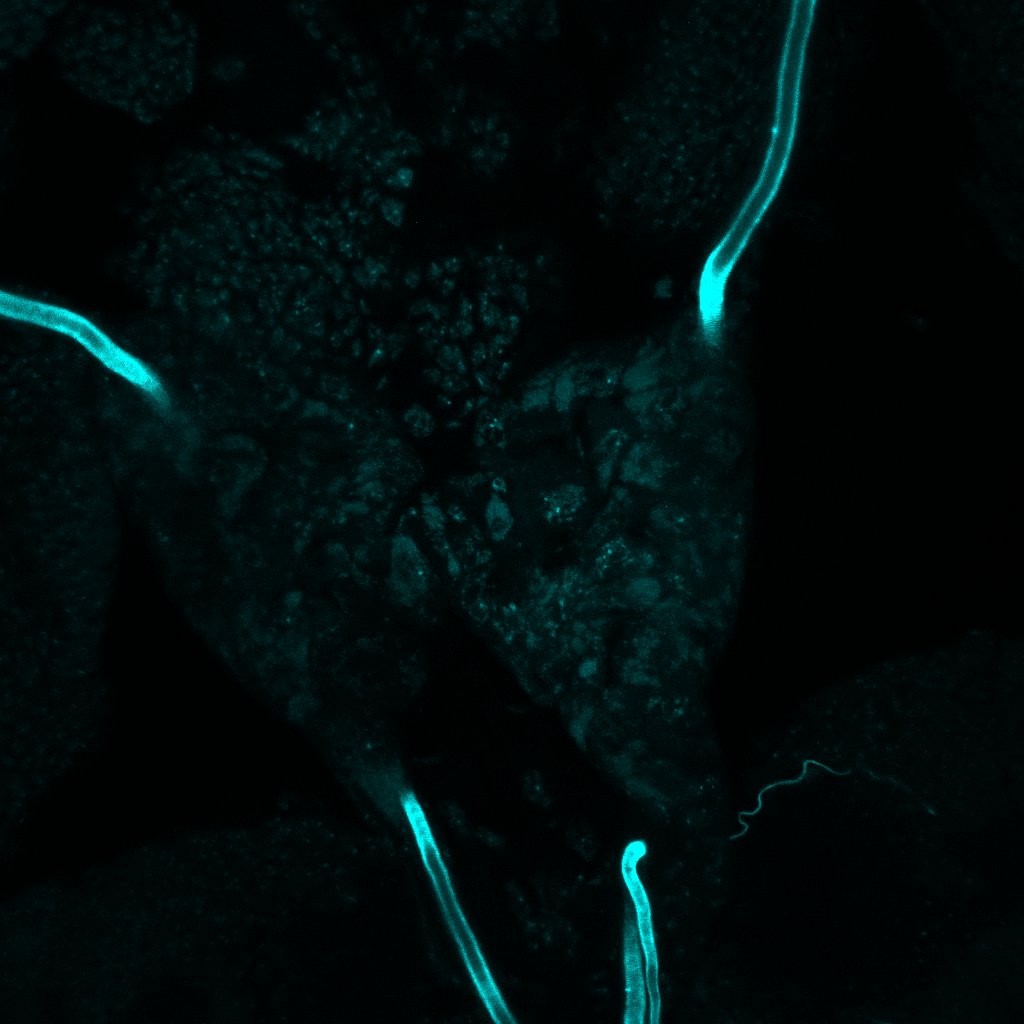

Supplement: Figure 3—figure supplement 3—source data 2. [file elife-105165-fig3-figsupp3-data2.zip › Figure 3-figure supplement 3 Source data 2/S7_Nup107KK_C1.jpg]

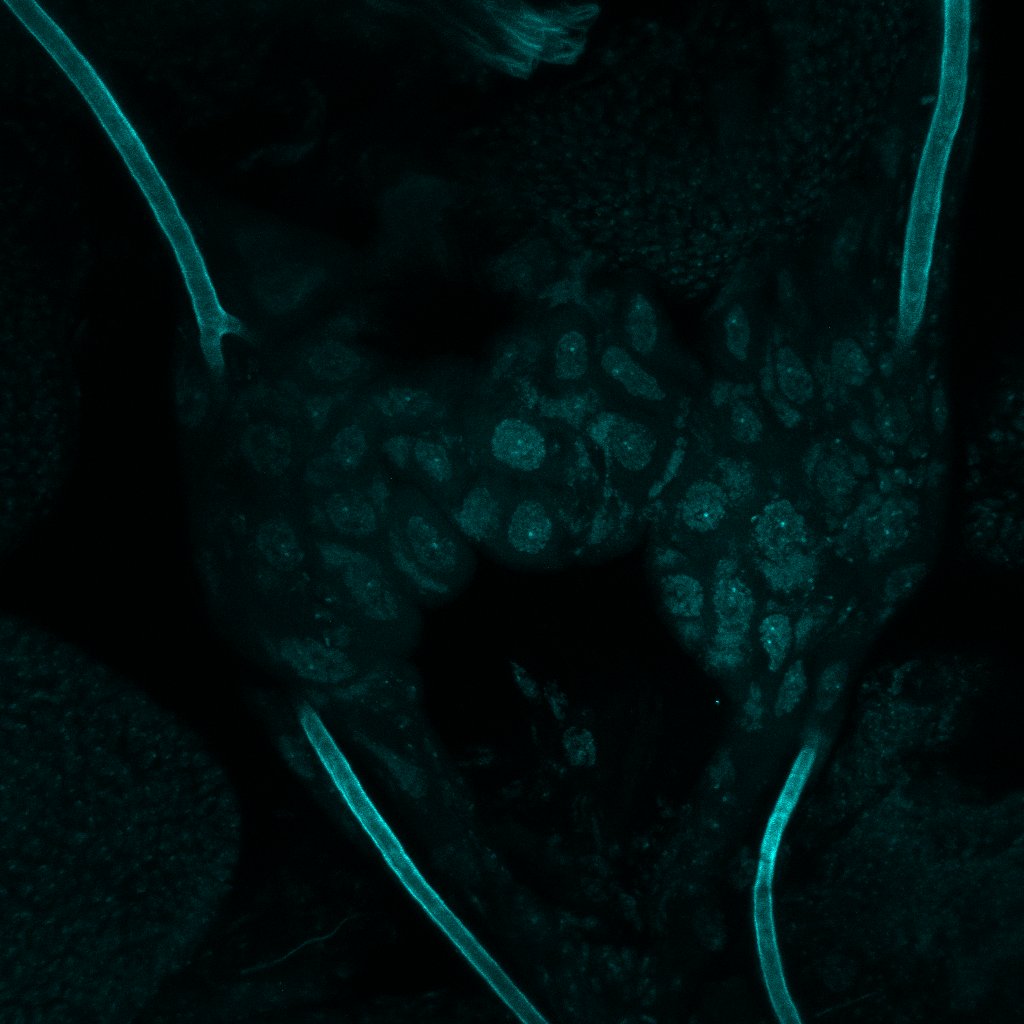

Supplement: Figure 3—figure supplement 3—source data 2. [file elife-105165-fig3-figsupp3-data2.zip › Figure 3-figure supplement 3 Source data 2/S7_Control_C1.jpg]

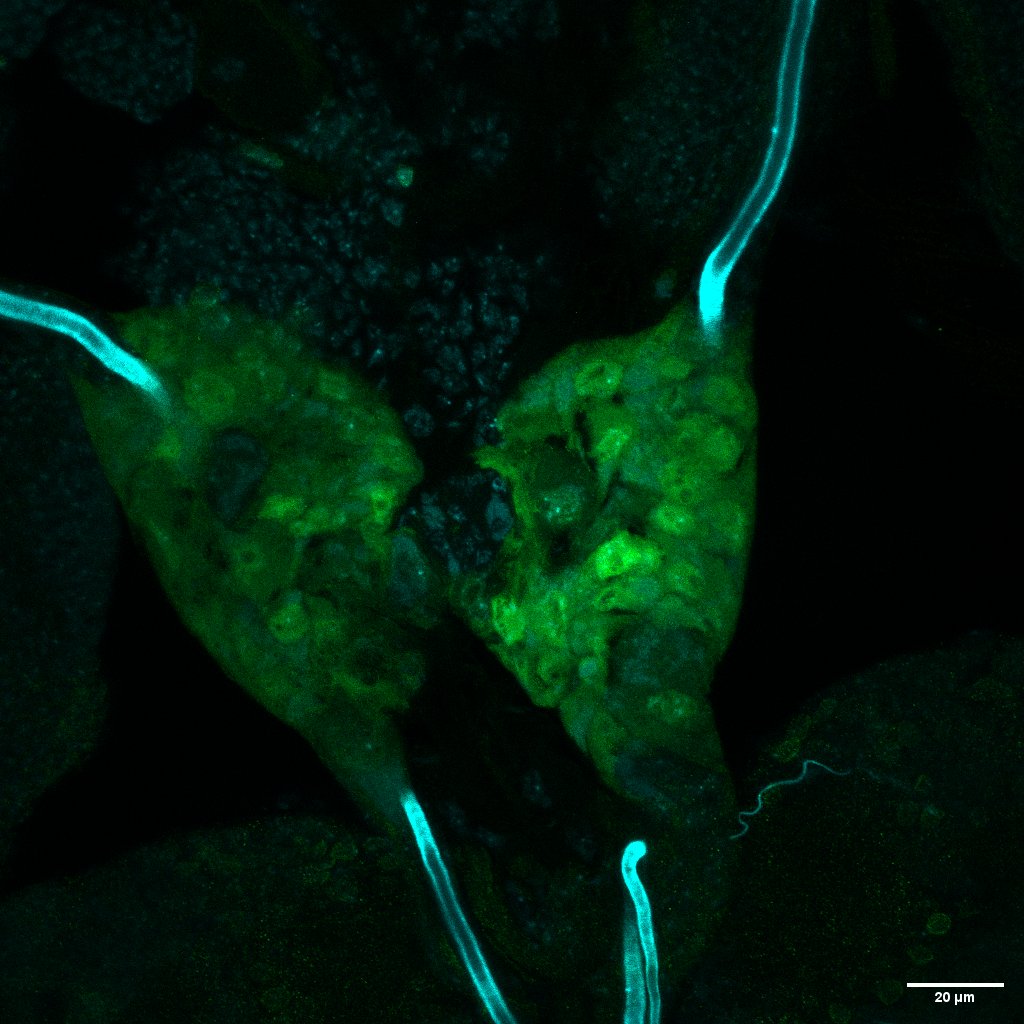

Supplement: Figure 3—figure supplement 3—source data 2. [file elife-105165-fig3-figsupp3-data2.zip › Figure 3-figure supplement 3 Source data 2/S7_Nup107KK.jpg]

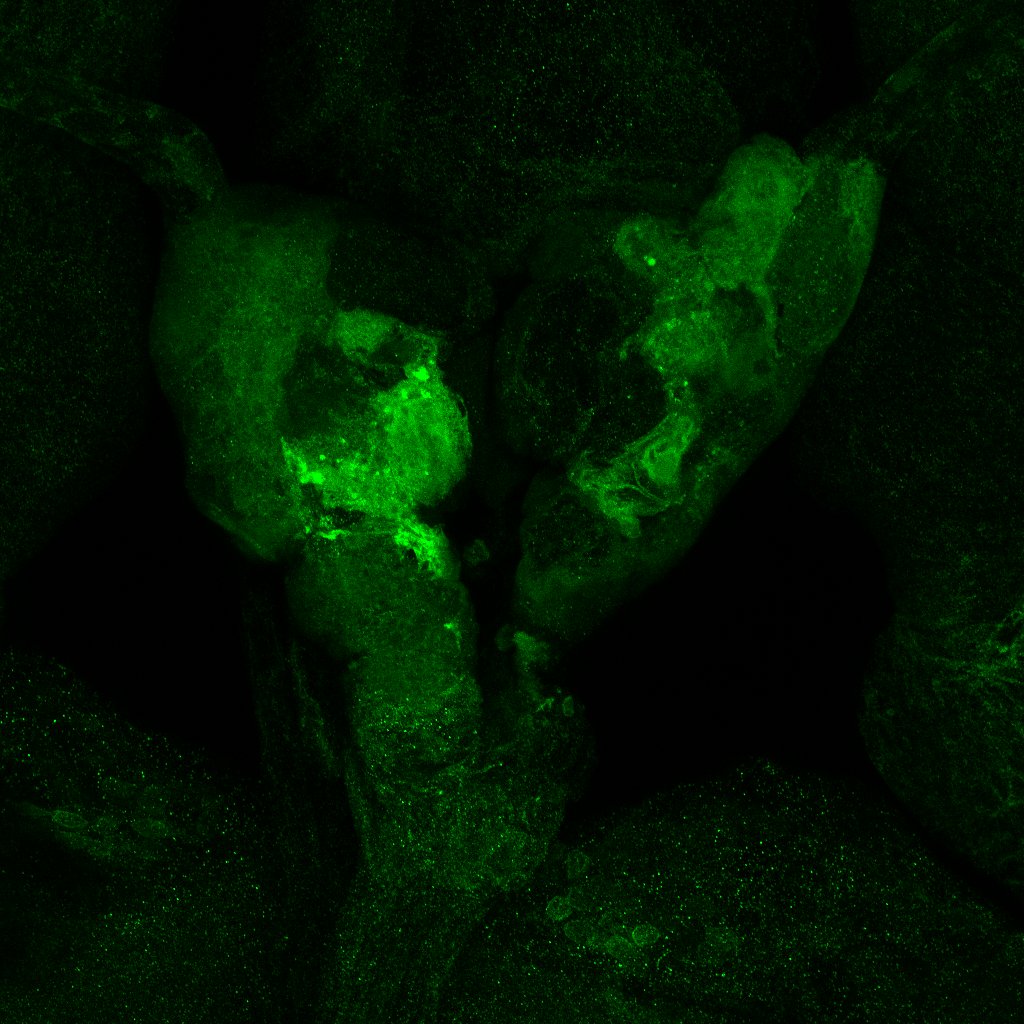

Supplement: Figure 3—figure supplement 3—source data 2. [file elife-105165-fig3-figsupp3-data2.zip › Figure 3-figure supplement 3 Source data 2/S7_Nup107GD_C2.jpg]

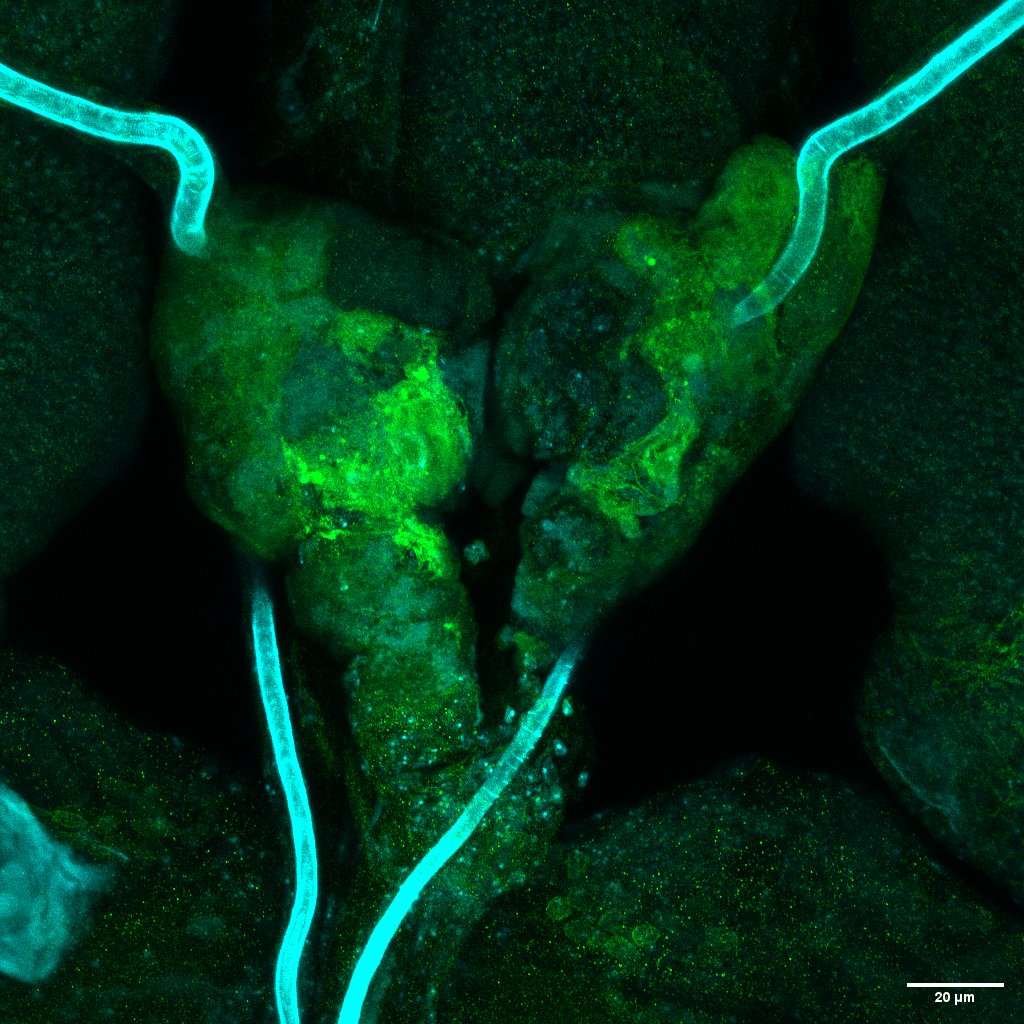

Supplement: Figure 3—figure supplement 3—source data 2. [file elife-105165-fig3-figsupp3-data2.zip › Figure 3-figure supplement 3 Source data 2/S7_Nup107GD.jpg]

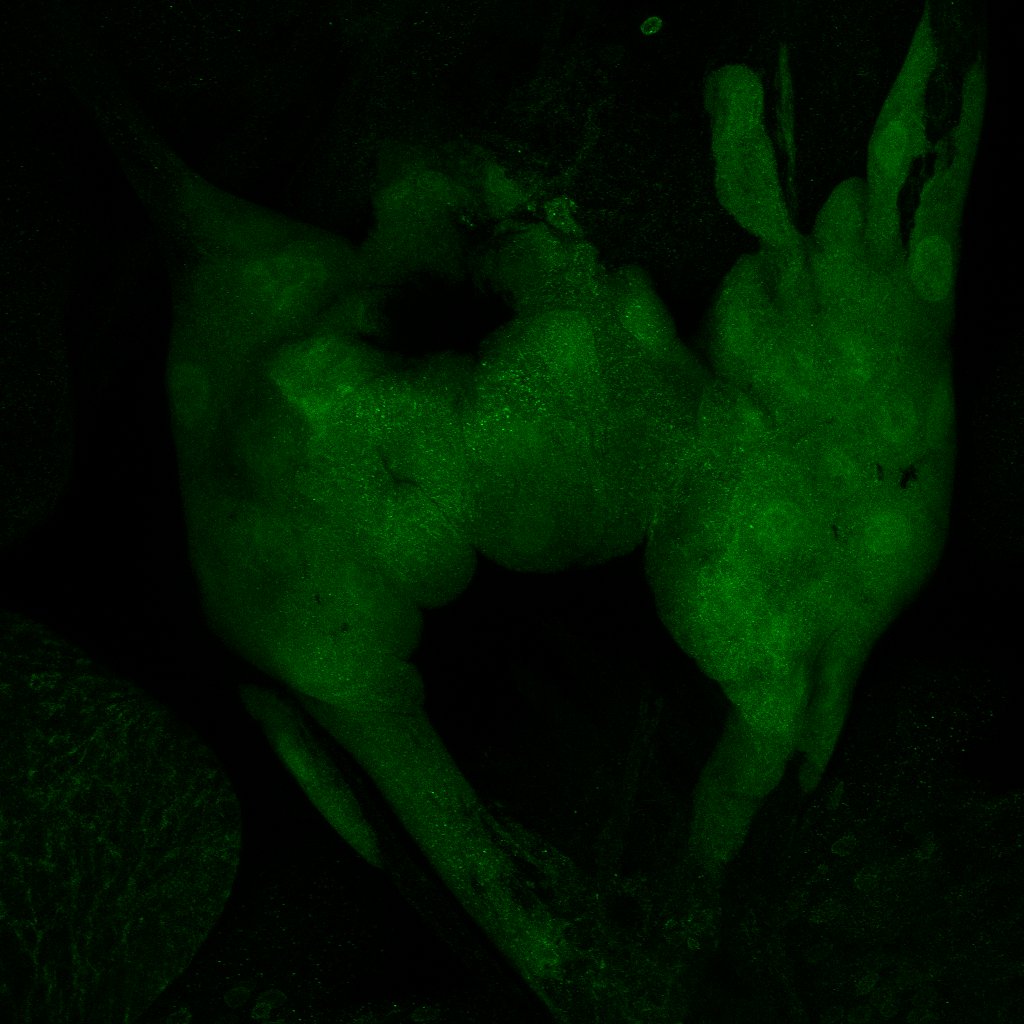

Supplement: Figure 3—figure supplement 3—source data 2. [file elife-105165-fig3-figsupp3-data2.zip › Figure 3-figure supplement 3 Source data 2/S7_Control_C2.jpg]

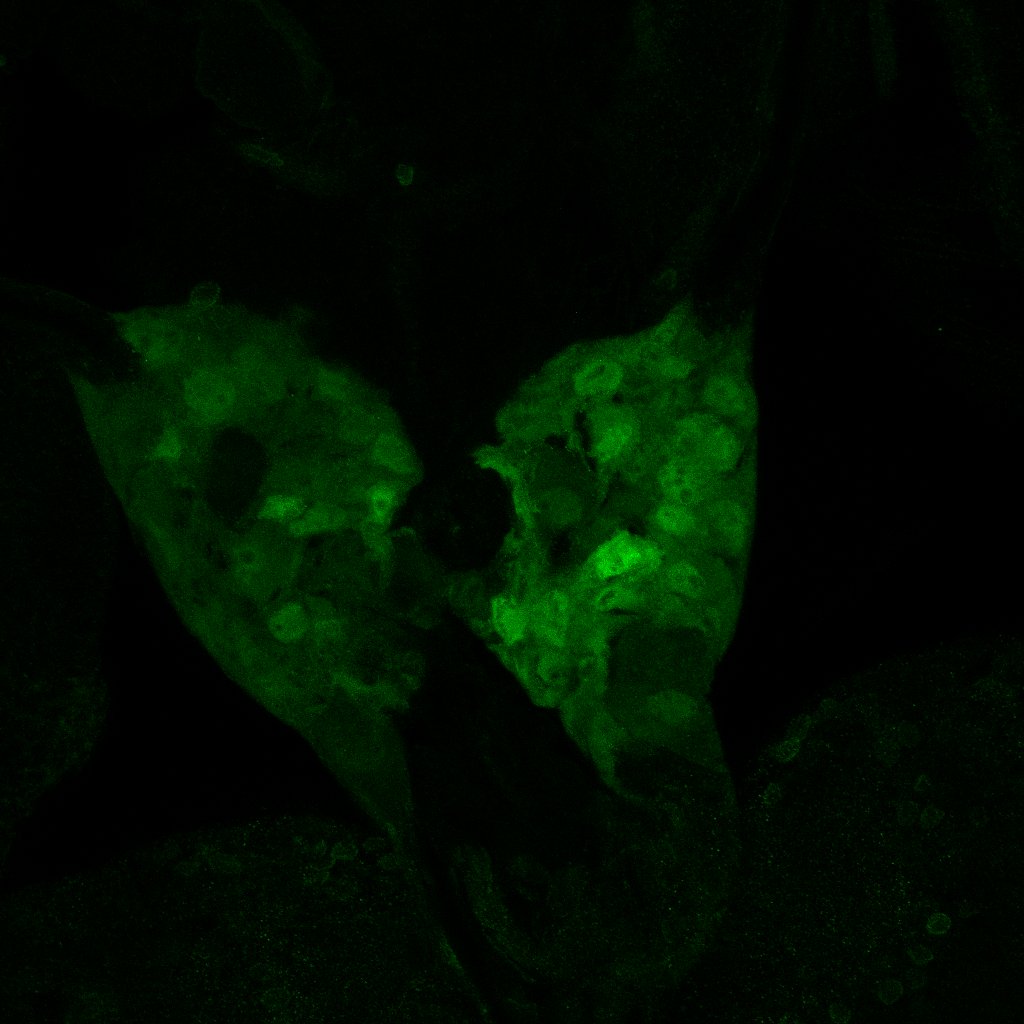

Supplement: Figure 3—figure supplement 3—source data 2. [file elife-105165-fig3-figsupp3-data2.zip › Figure 3-figure supplement 3 Source data 2/S7_Nup107KK_C2.jpg]

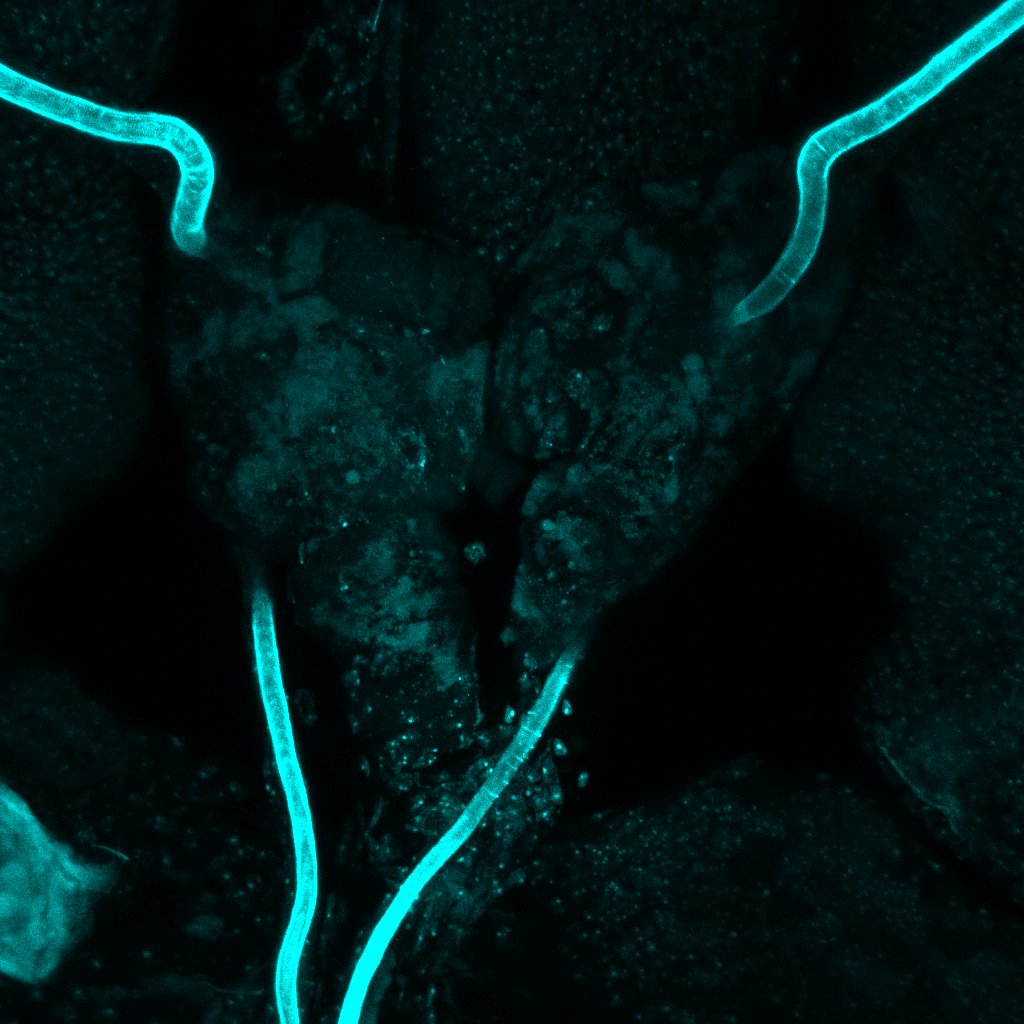

Supplement: Figure 3—figure supplement 3—source data 2. [file elife-105165-fig3-figsupp3-data2.zip › Figure 3-figure supplement 3 Source data 2/S7_Nup107GD_C1.jpg]

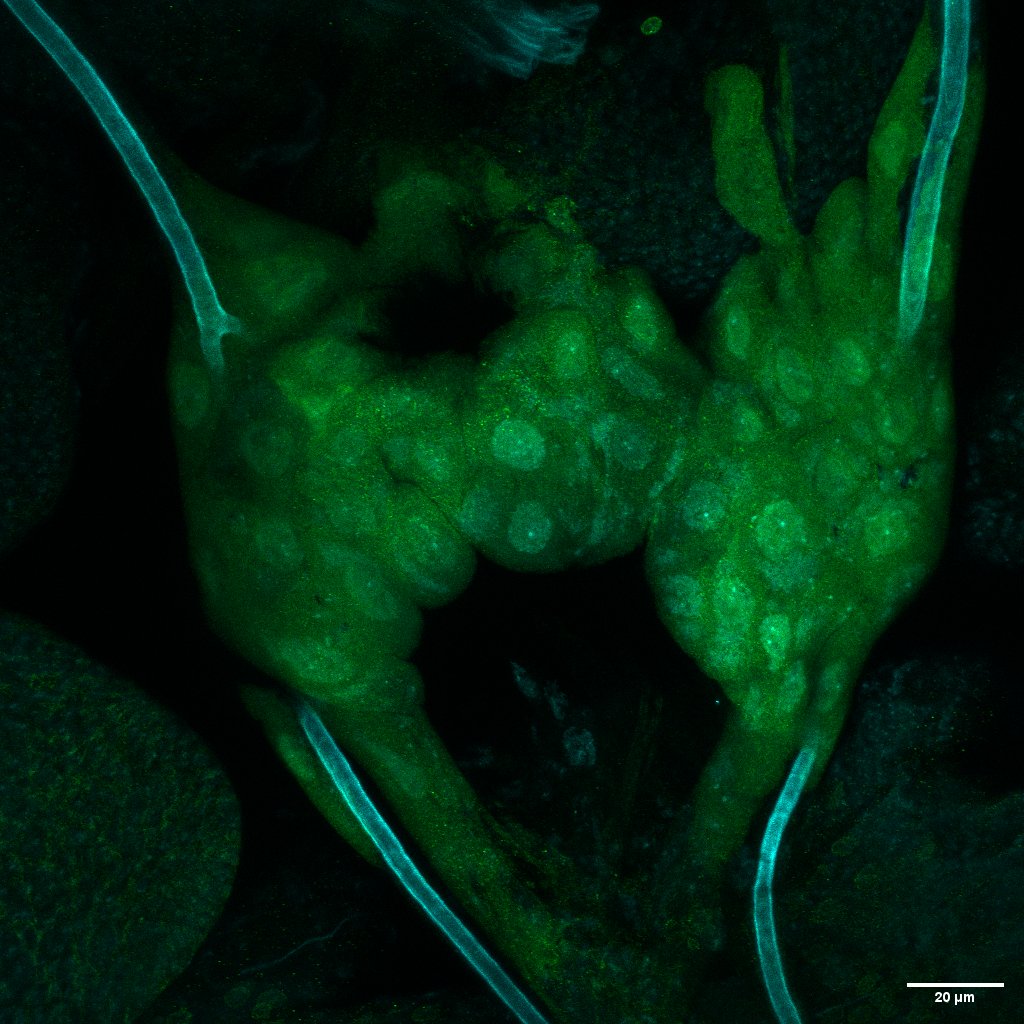

Supplement: Figure 3—figure supplement 3—source data 2. [file elife-105165-fig3-figsupp3-data2.zip › Figure 3-figure supplement 3 Source data 2/S7_Control.jpg]

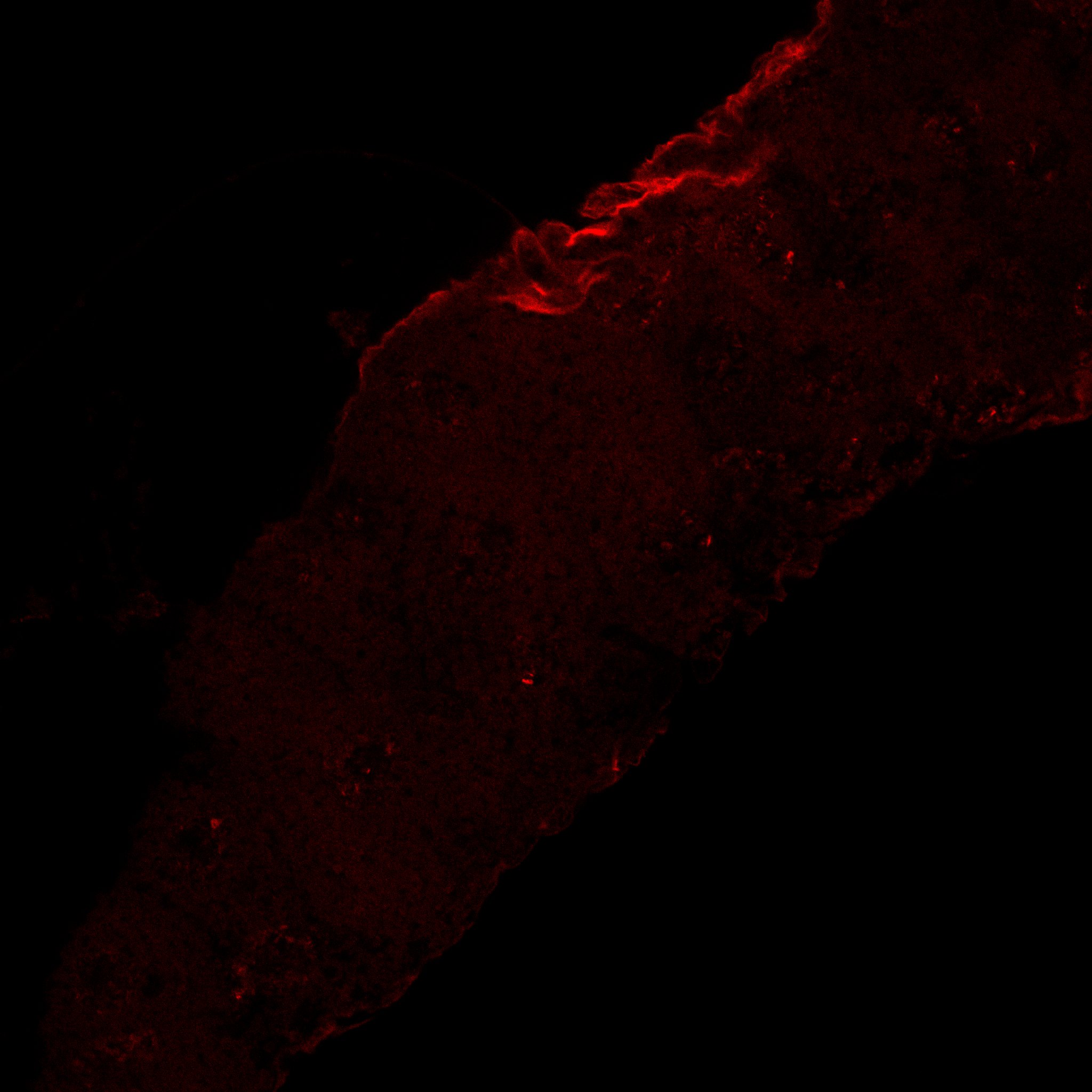

Supplement: Figure 5—source data 2. [file elife-105165-fig5-data2.zip › Figure 5 source data 2/5C_C3-60x_0022.jpg]

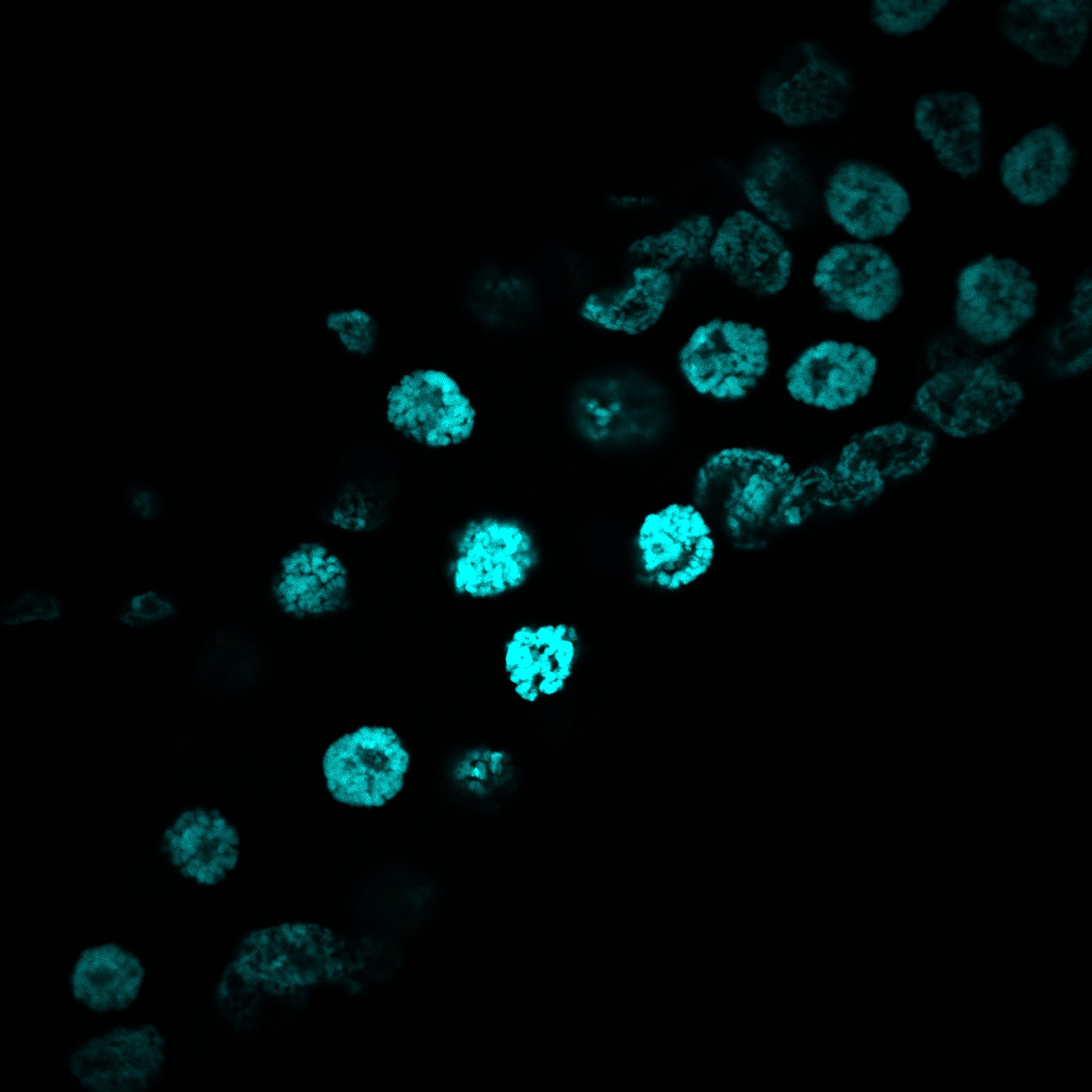

Supplement: Figure 5—source data 2. [file elife-105165-fig5-data2.zip › Figure 5 source data 2/5C_C1-60x_0022.jpg]

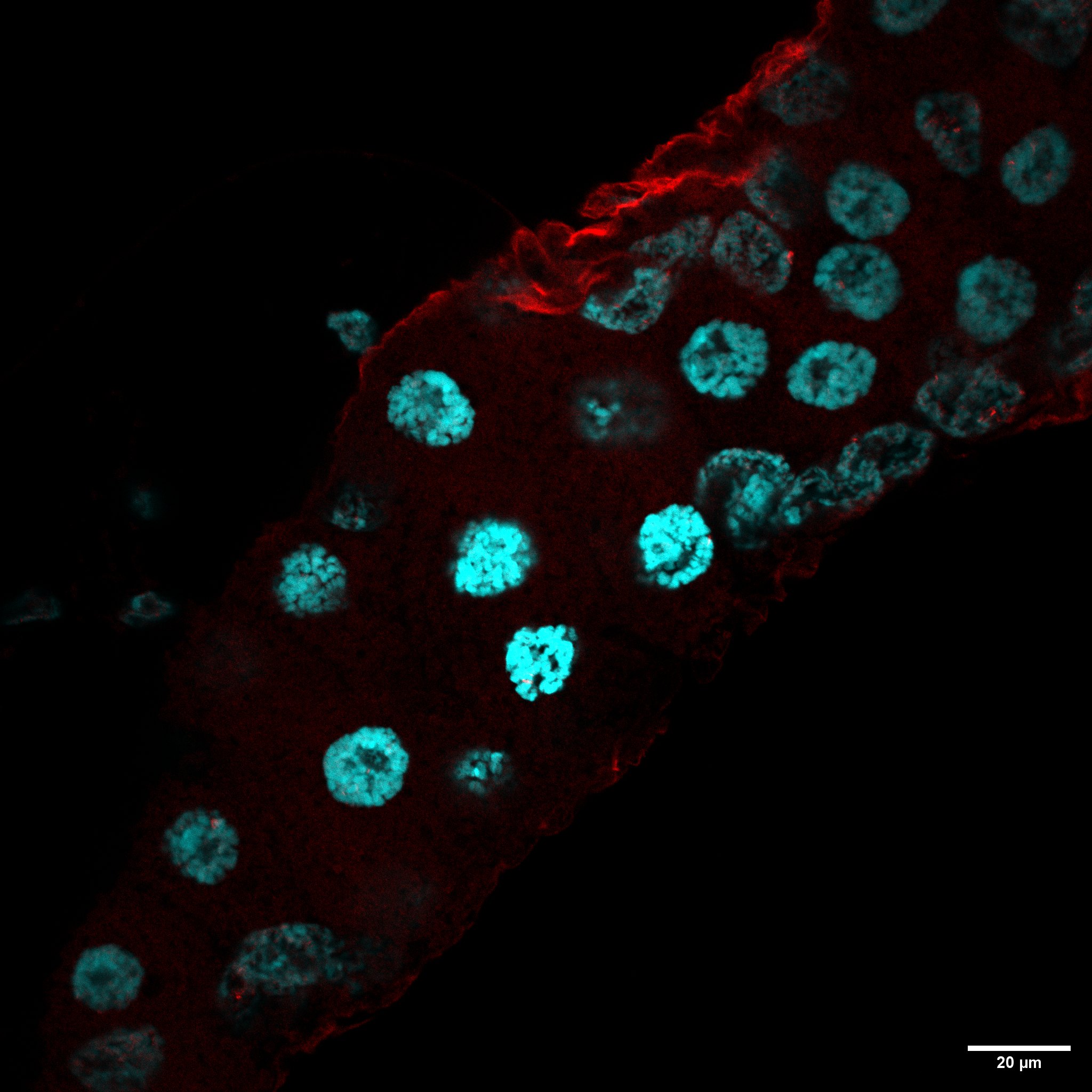

Supplement: Figure 5—source data 2. [file elife-105165-fig5-data2.zip › Figure 5 source data 2/5C_60x_0022.jpg]

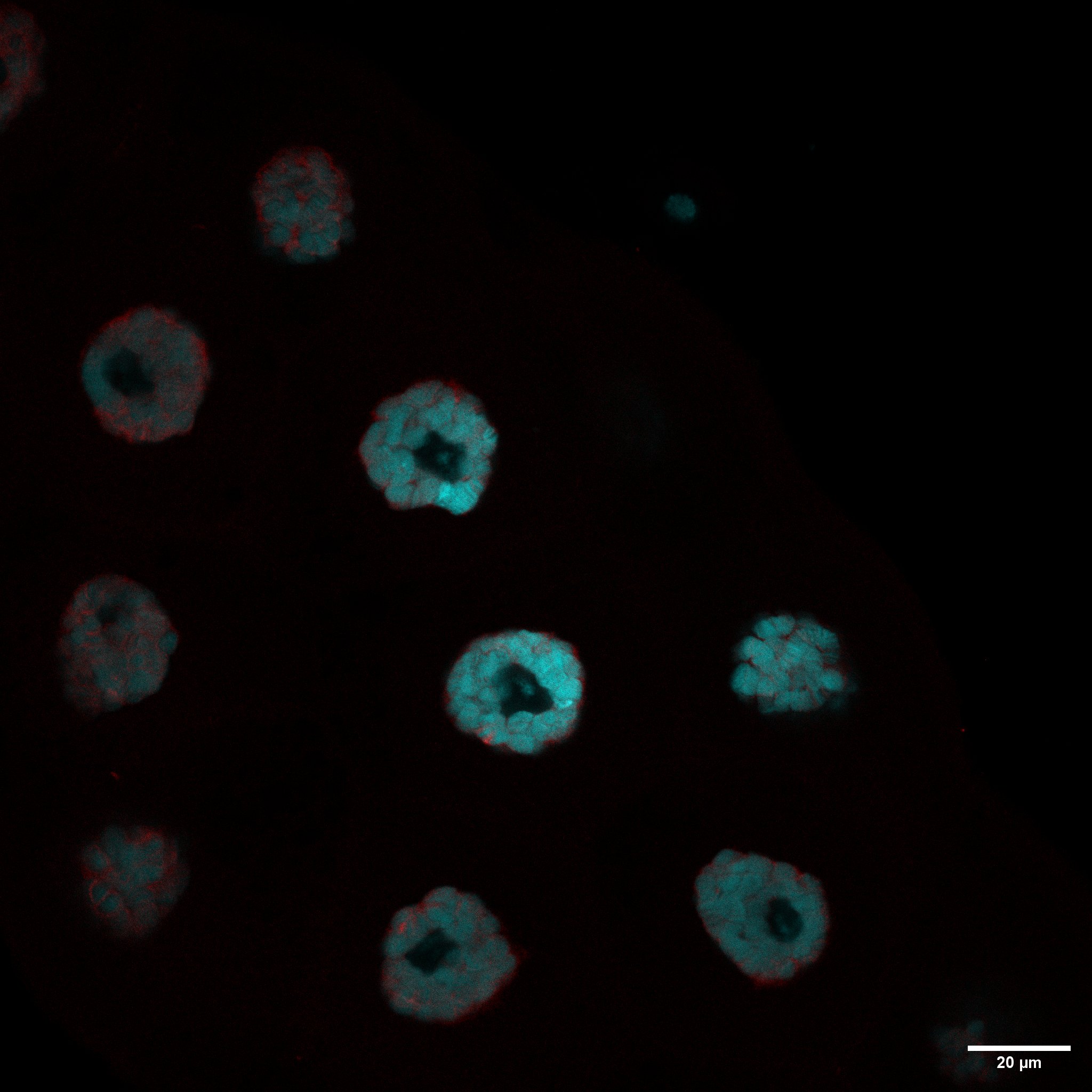

Supplement: Figure 5—source data 2. [file elife-105165-fig5-data2.zip › Figure 5 source data 2/5F_60x.jpg]

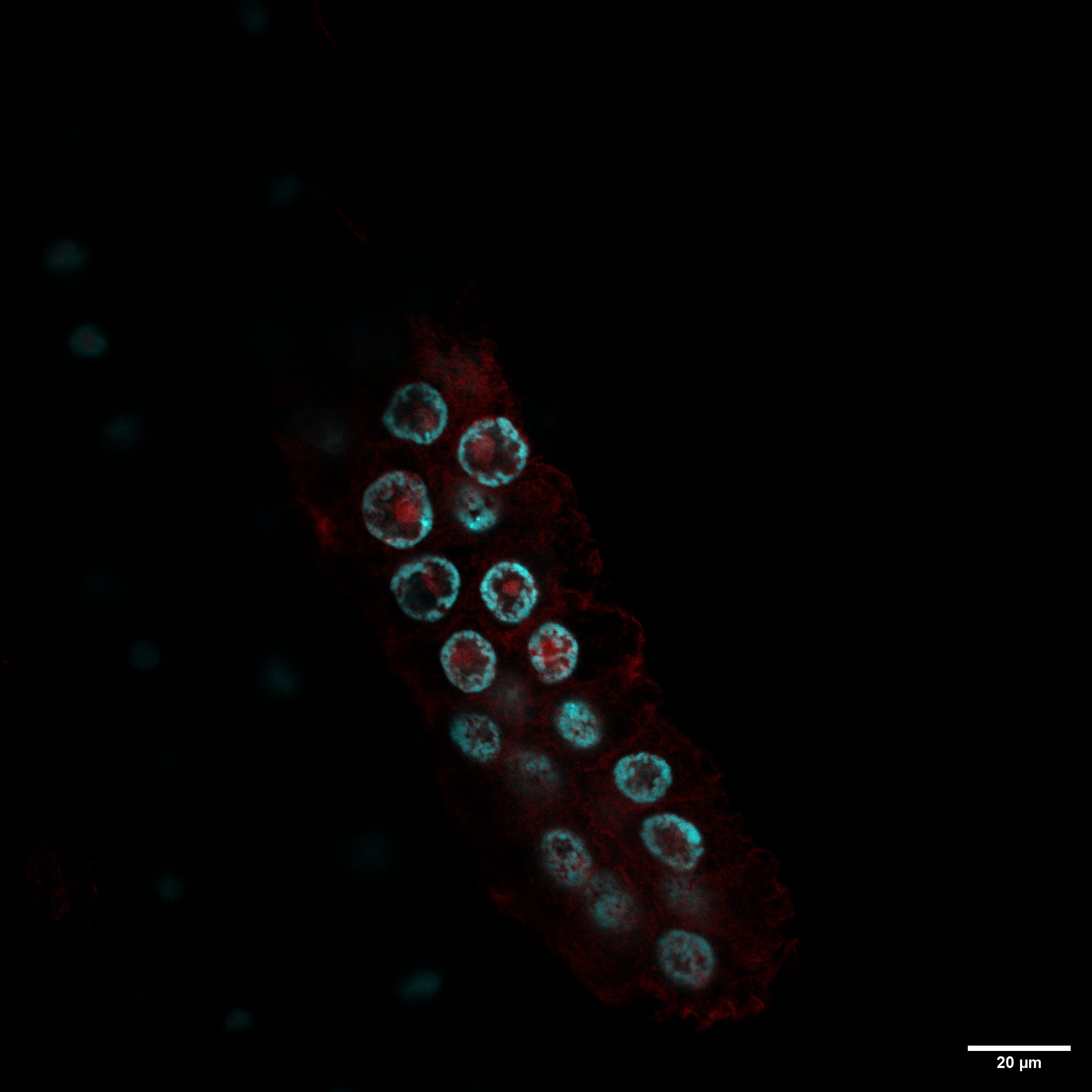

Supplement: Figure 5—source data 2. [file elife-105165-fig5-data2.zip › Figure 5 source data 2/5E_60x_0005.jpg]

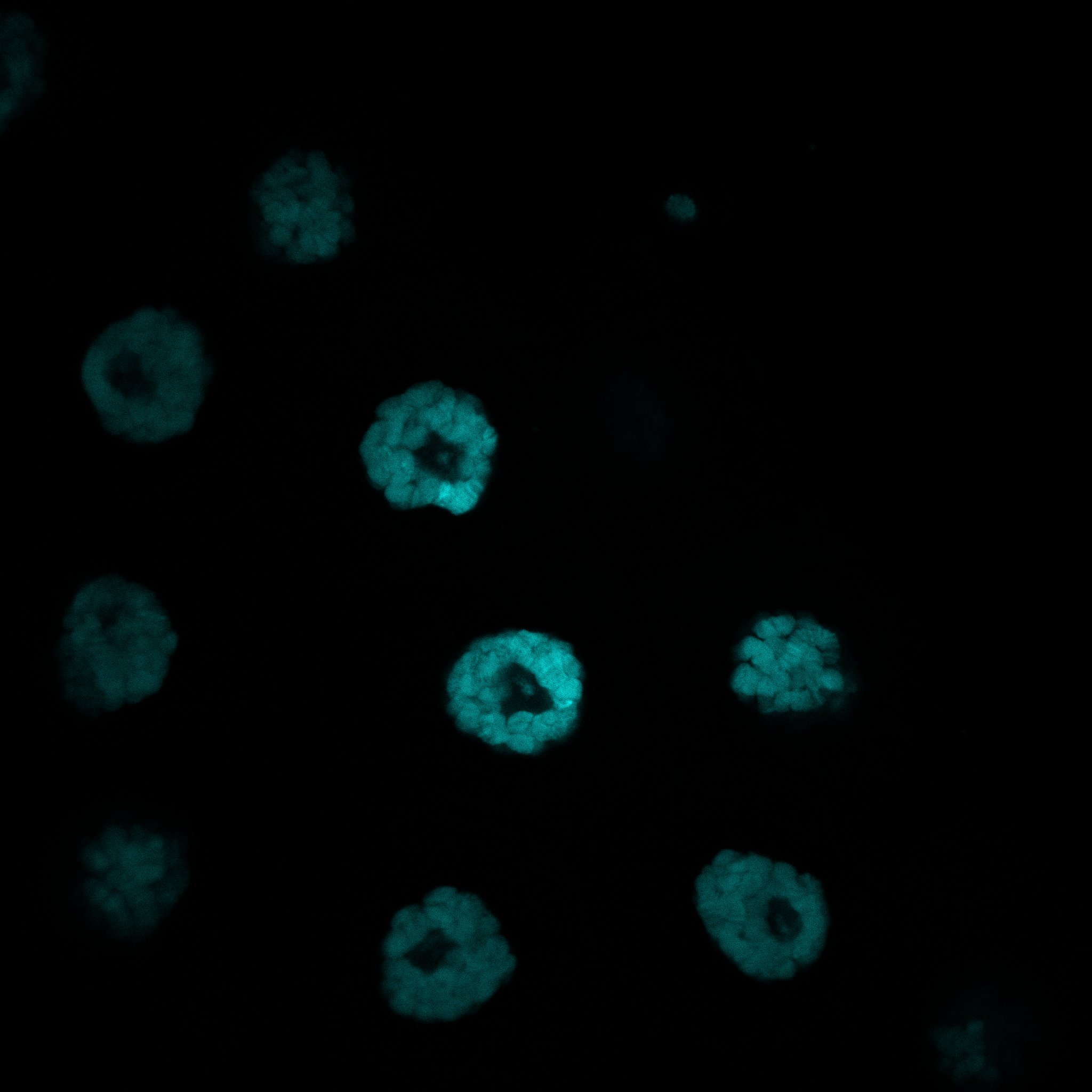

Supplement: Figure 5—source data 2. [file elife-105165-fig5-data2.zip › Figure 5 source data 2/5F_C1-60x.jpg]

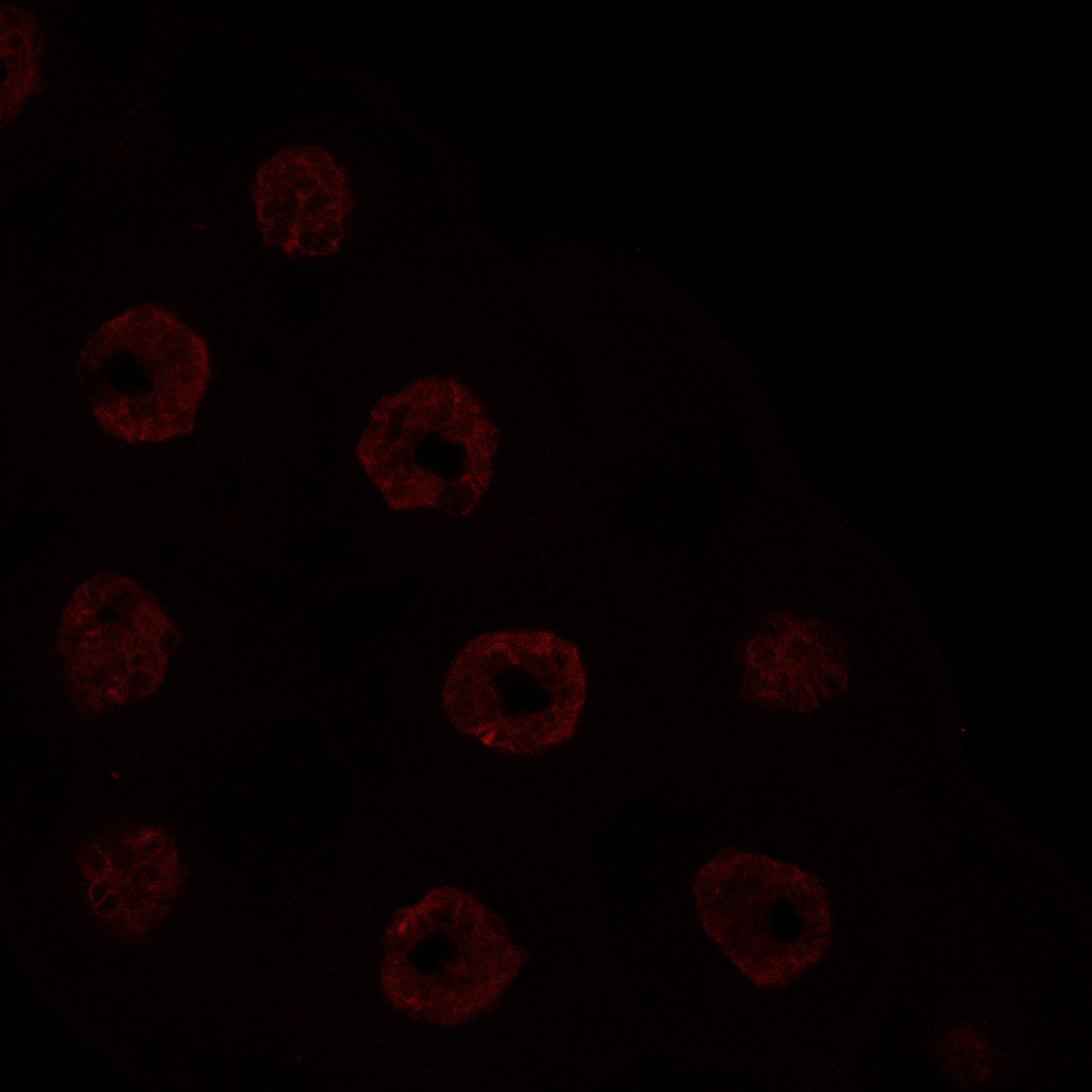

Supplement: Figure 5—source data 2. [file elife-105165-fig5-data2.zip › Figure 5 source data 2/5F_C3-60x.jpg]

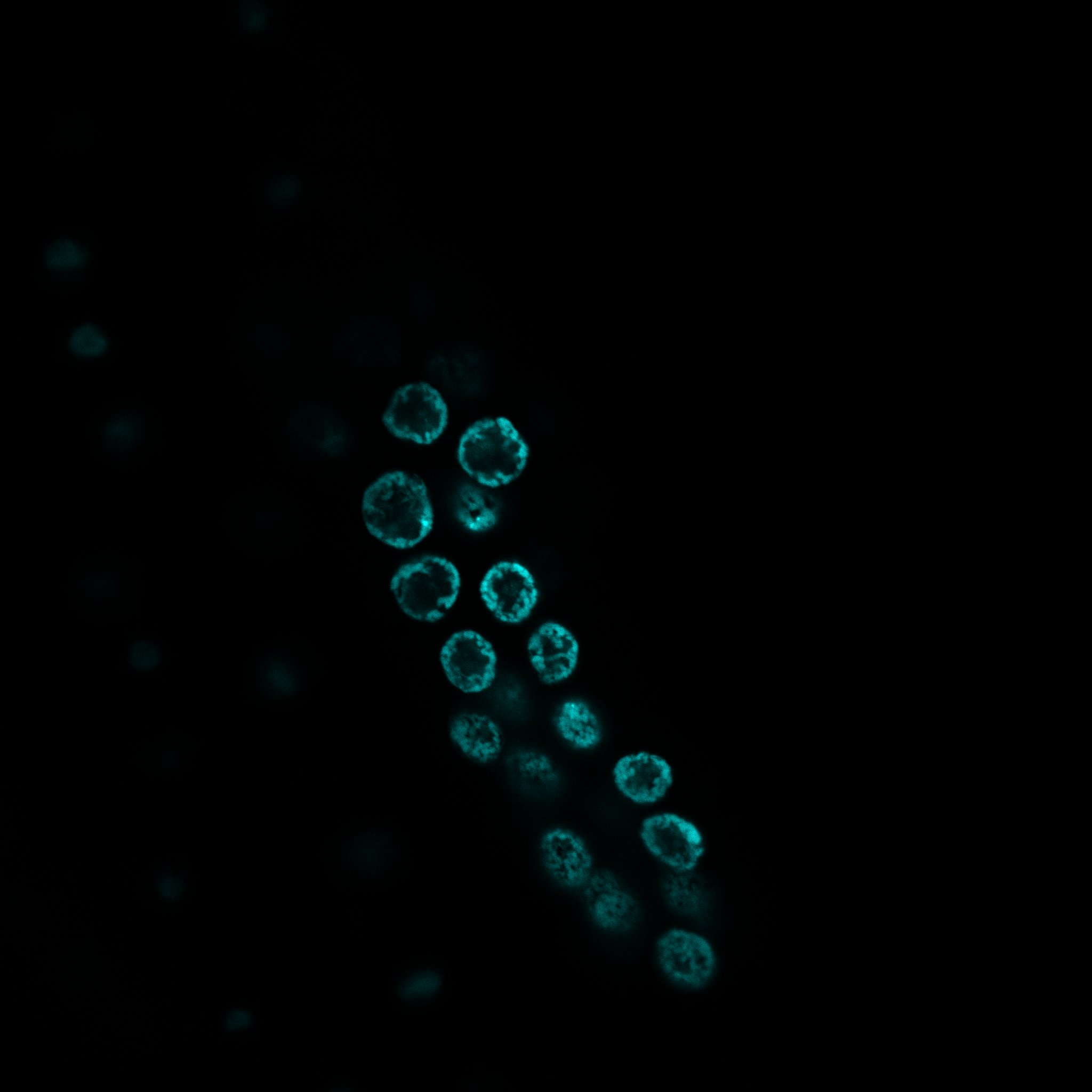

Supplement: Figure 5—source data 2. [file elife-105165-fig5-data2.zip › Figure 5 source data 2/5E_C1-60x_0005.jpg]

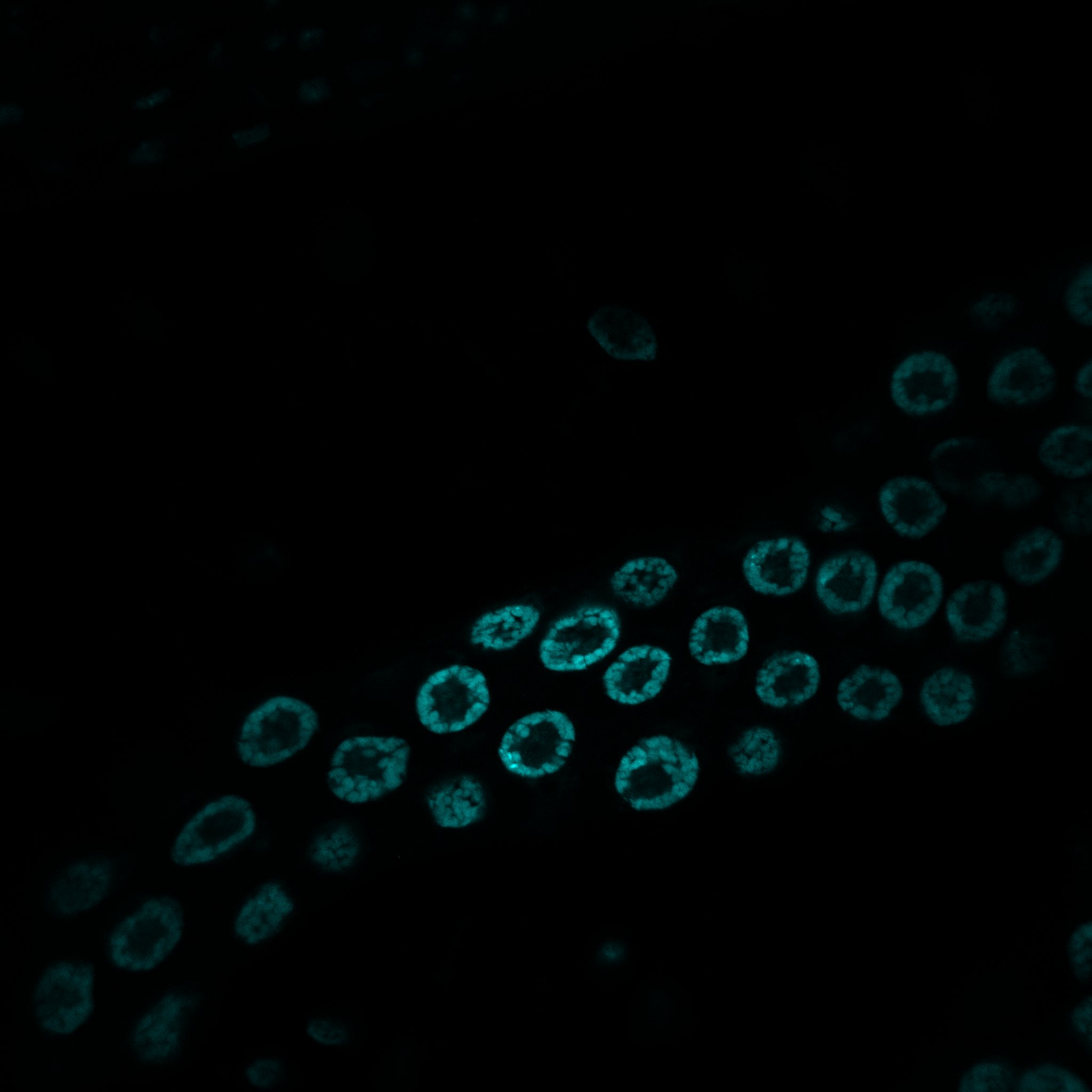

Supplement: Figure 5—source data 2. [file elife-105165-fig5-data2.zip › Figure 5 source data 2/5B_C1-60x_0003.jpg]

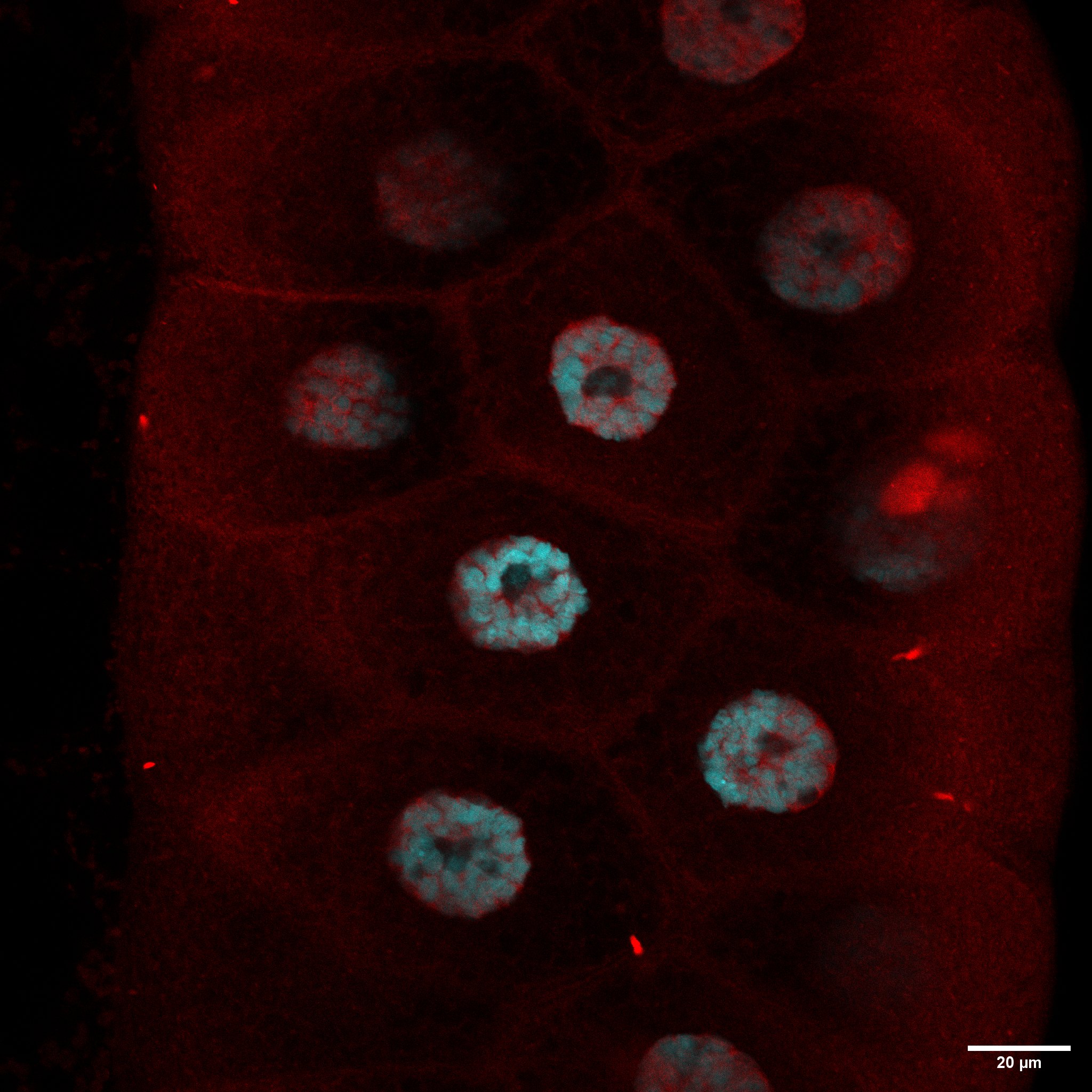

Supplement: Figure 5—source data 2. [file elife-105165-fig5-data2.zip › Figure 5 source data 2/5A_60x_0006.jpg]

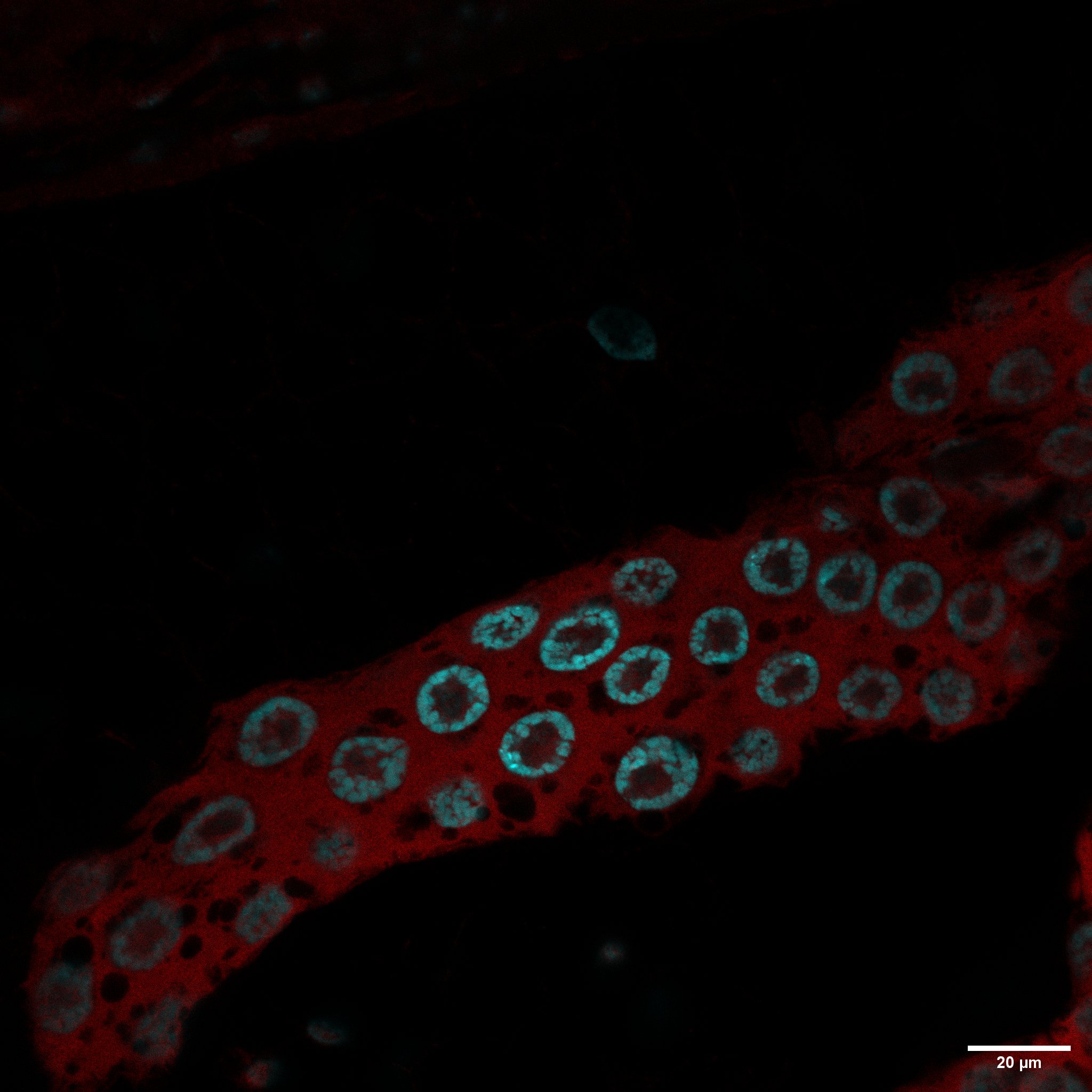

Supplement: Figure 5—source data 2. [file elife-105165-fig5-data2.zip › Figure 5 source data 2/5B_60x_0003.jpg]

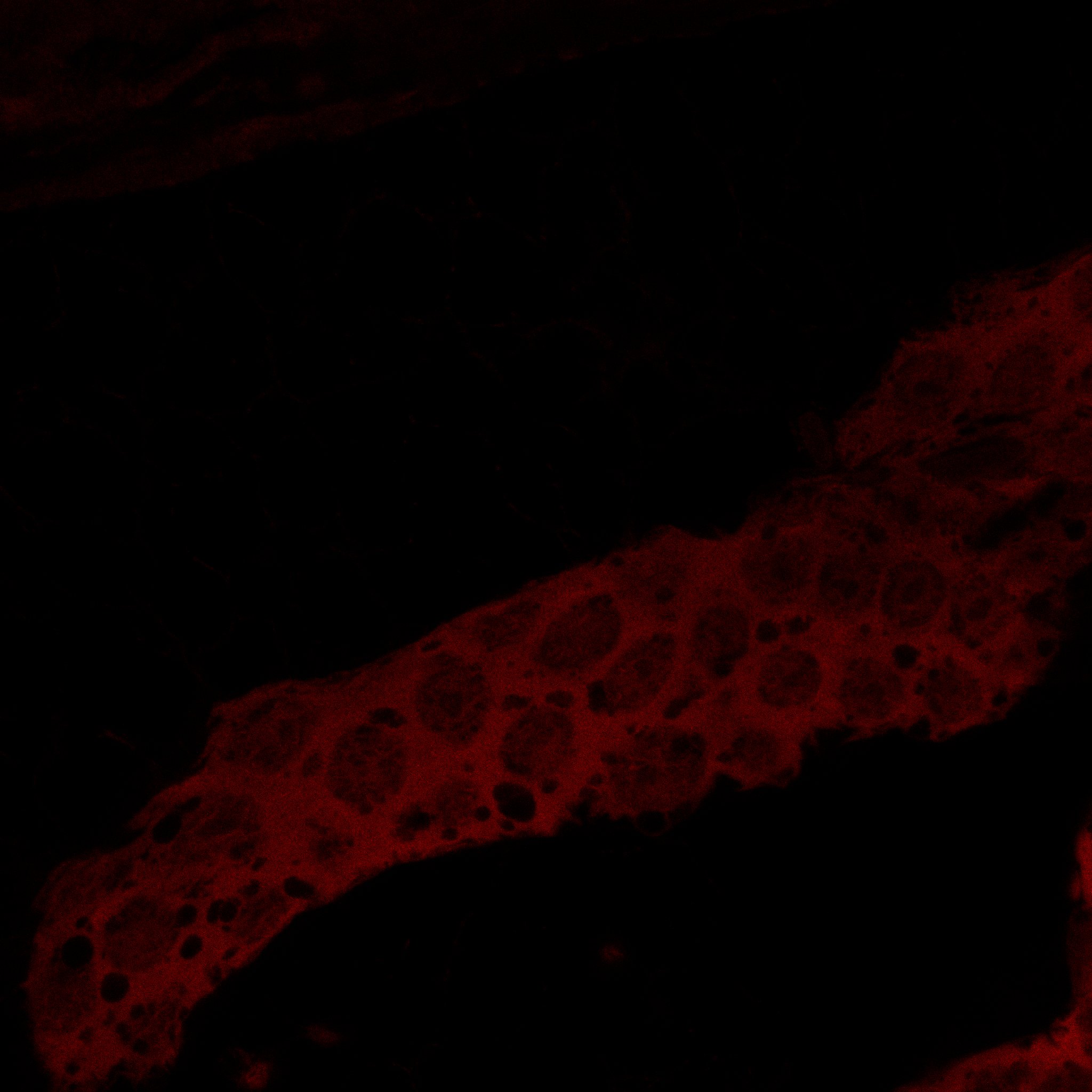

Supplement: Figure 5—source data 2. [file elife-105165-fig5-data2.zip › Figure 5 source data 2/5B_C3-60x_0003.jpg]

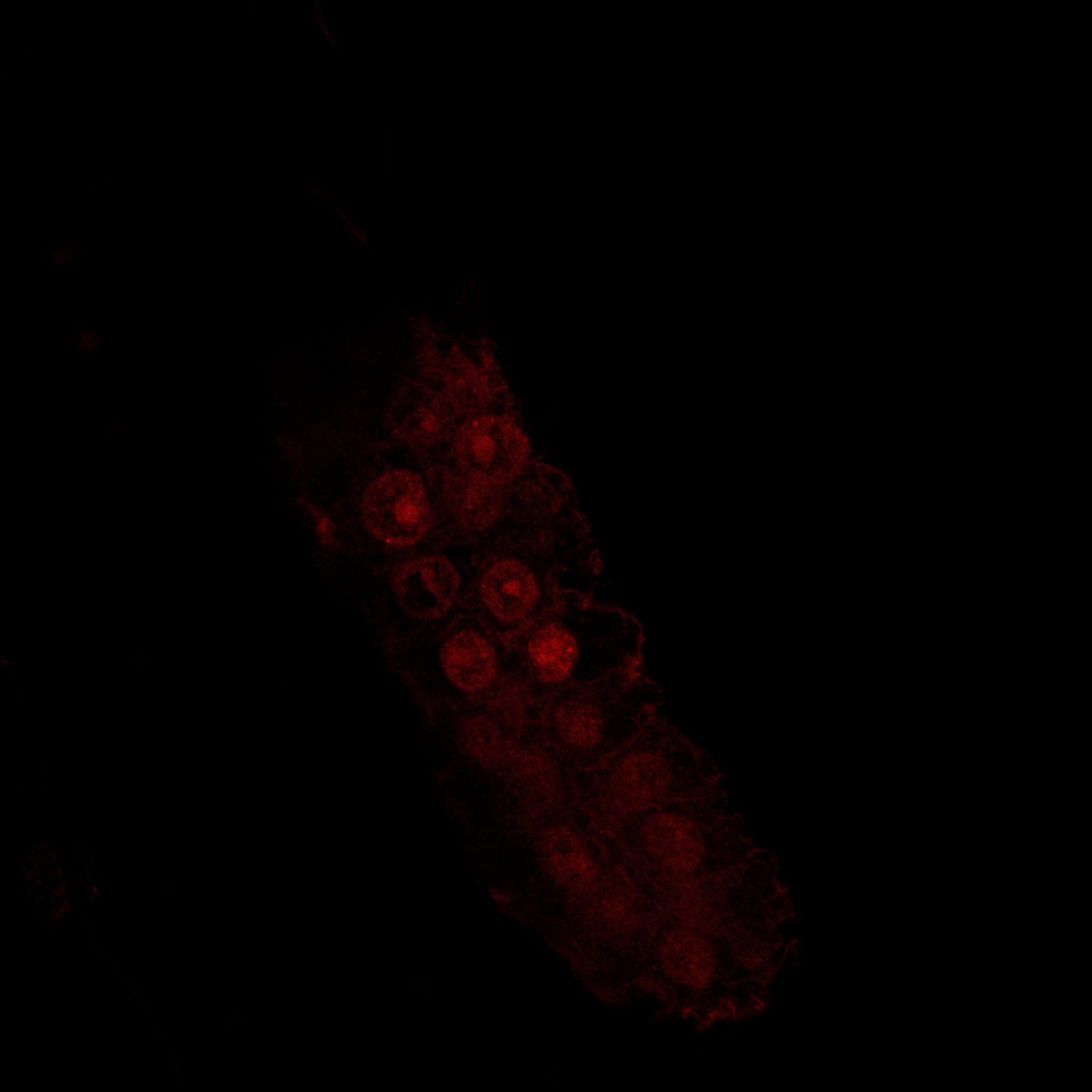

Supplement: Figure 5—source data 2. [file elife-105165-fig5-data2.zip › Figure 5 source data 2/5E_C3-60x_0005.jpg]

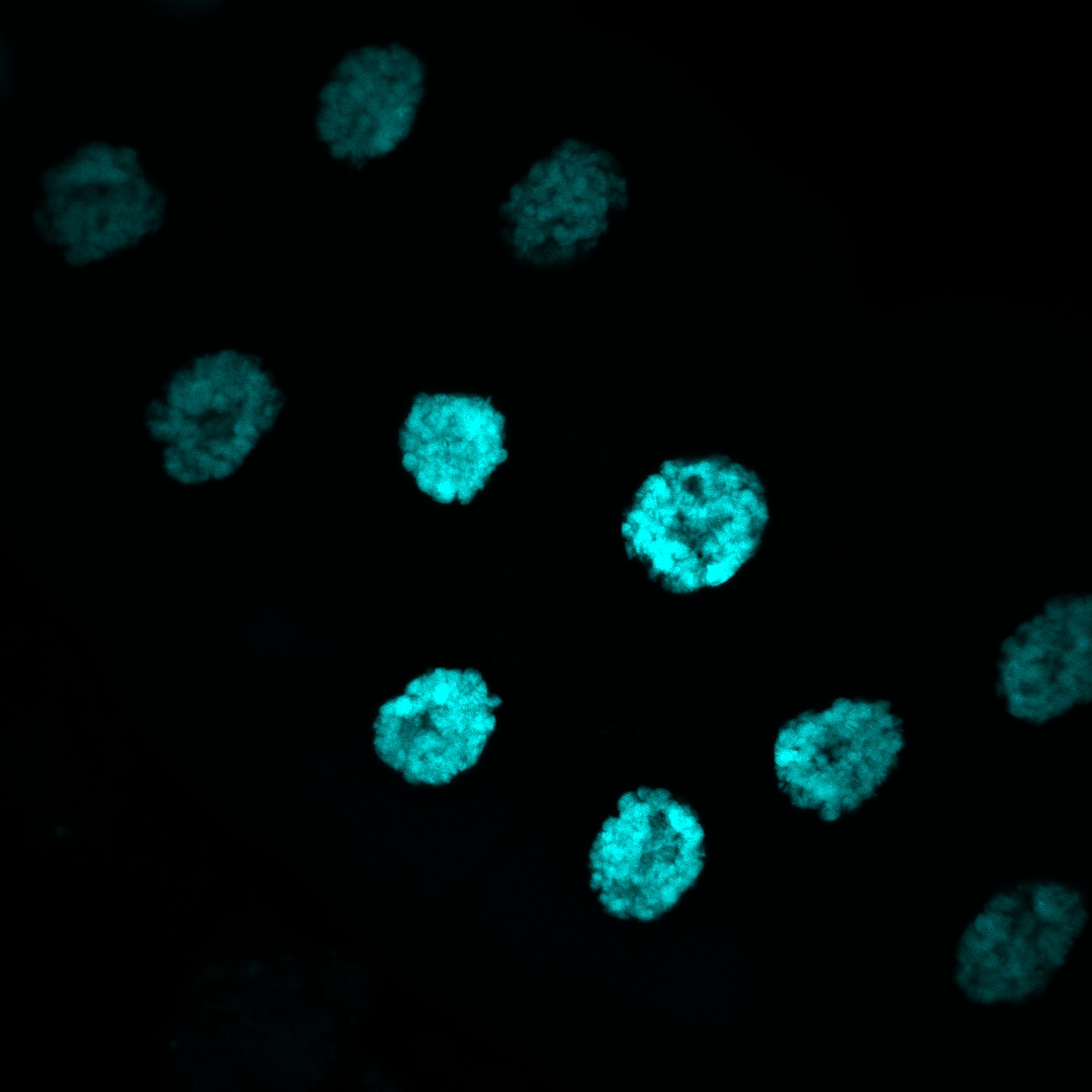

Supplement: Figure 5—source data 2. [file elife-105165-fig5-data2.zip › Figure 5 source data 2/5D_C1-MAX_60x_0002.jpg]

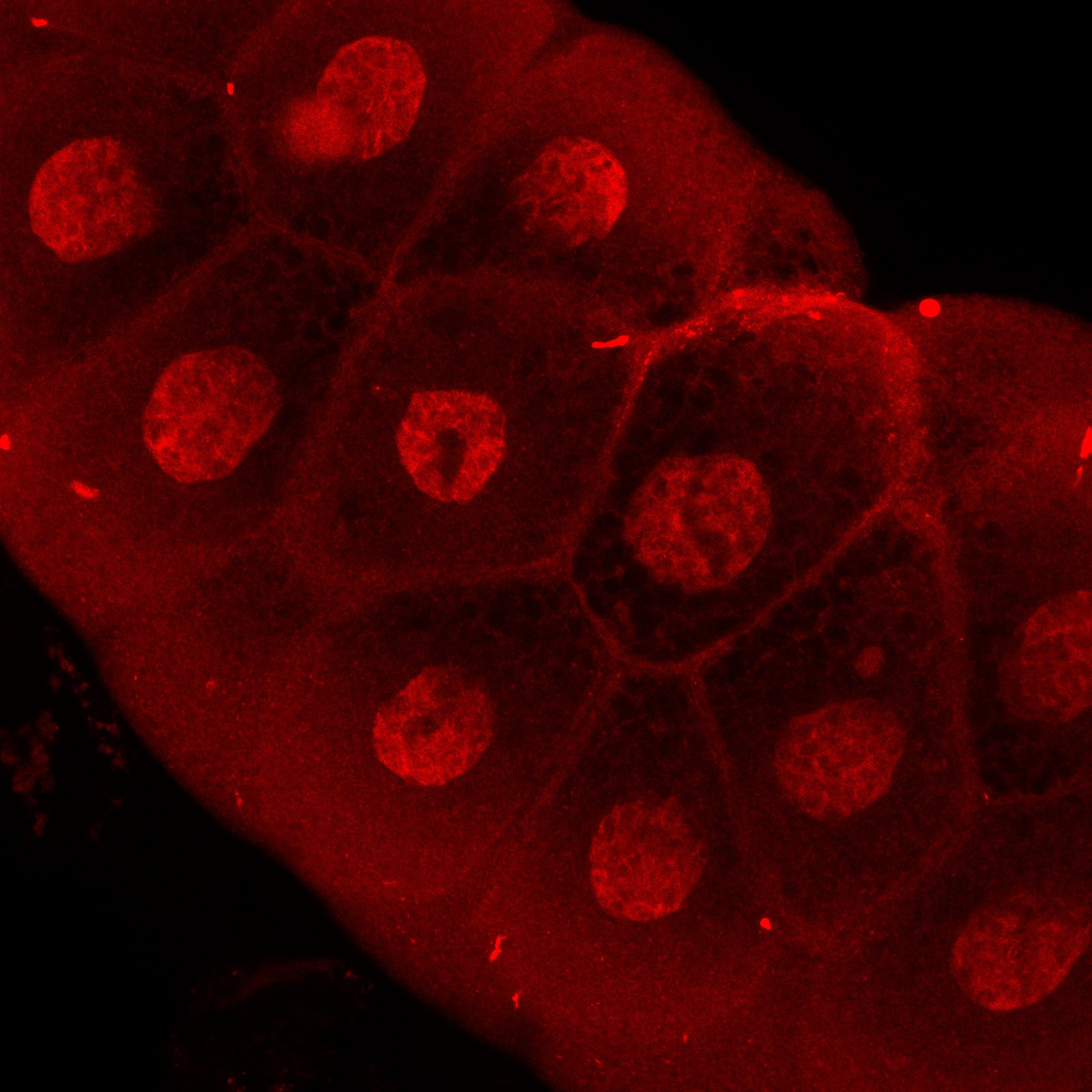

Supplement: Figure 5—source data 2. [file elife-105165-fig5-data2.zip › Figure 5 source data 2/5D_C3-MAX_60x_0002.jpg]

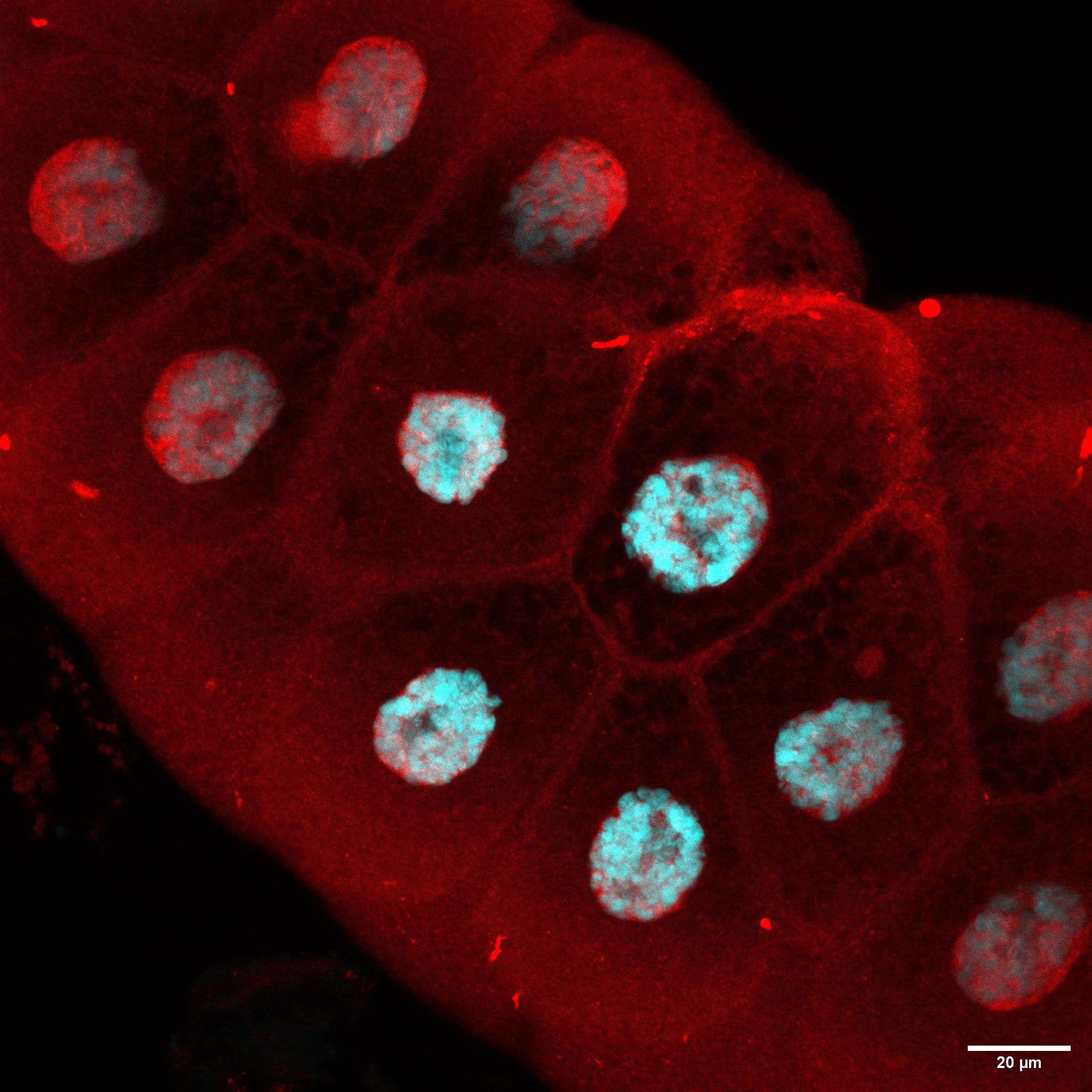

Supplement: Figure 5—source data 2. [file elife-105165-fig5-data2.zip › Figure 5 source data 2/5D_MAX_60x_0002.jpg]

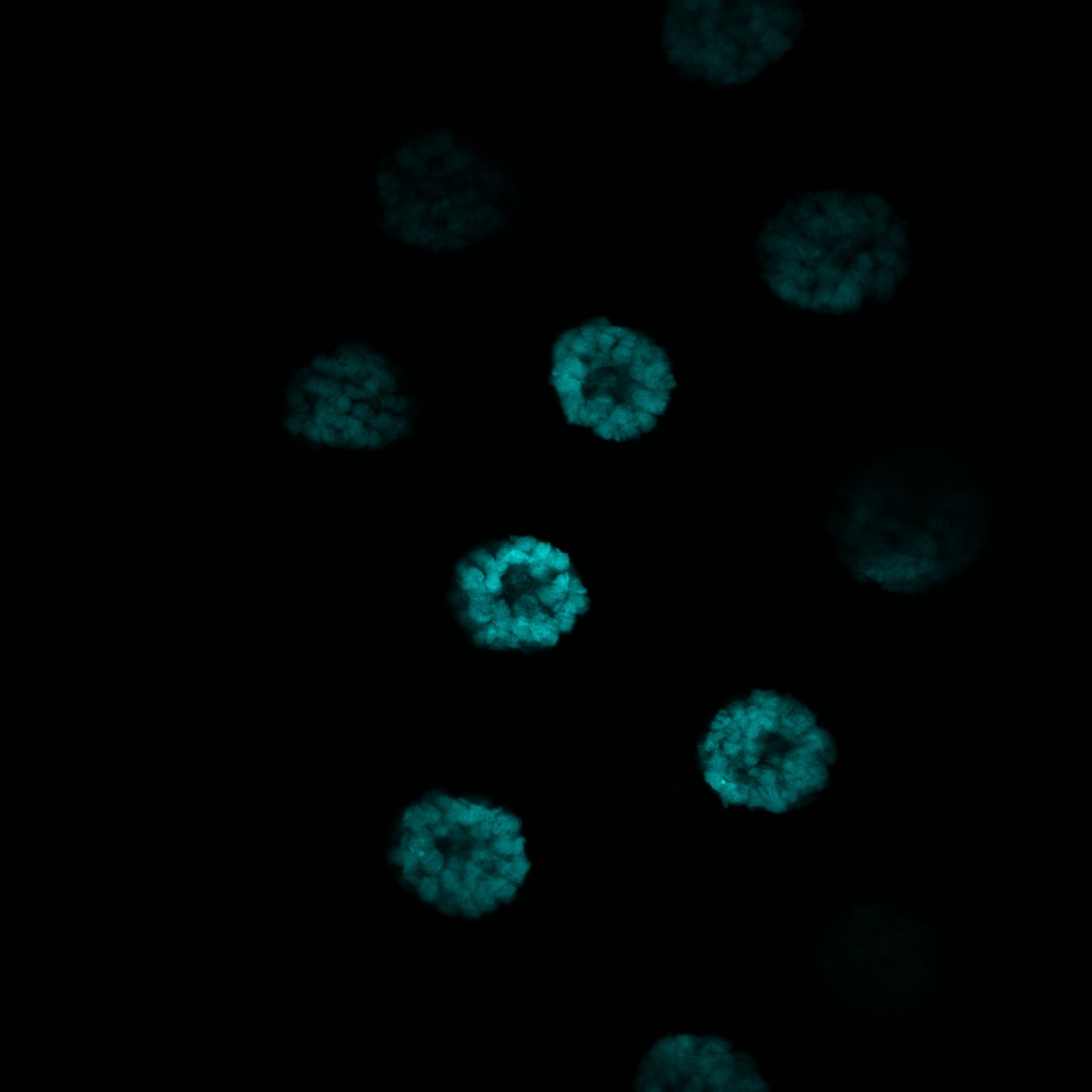

Supplement: Figure 5—source data 2. [file elife-105165-fig5-data2.zip › Figure 5 source data 2/5A_C1-60x_0006.jpg]

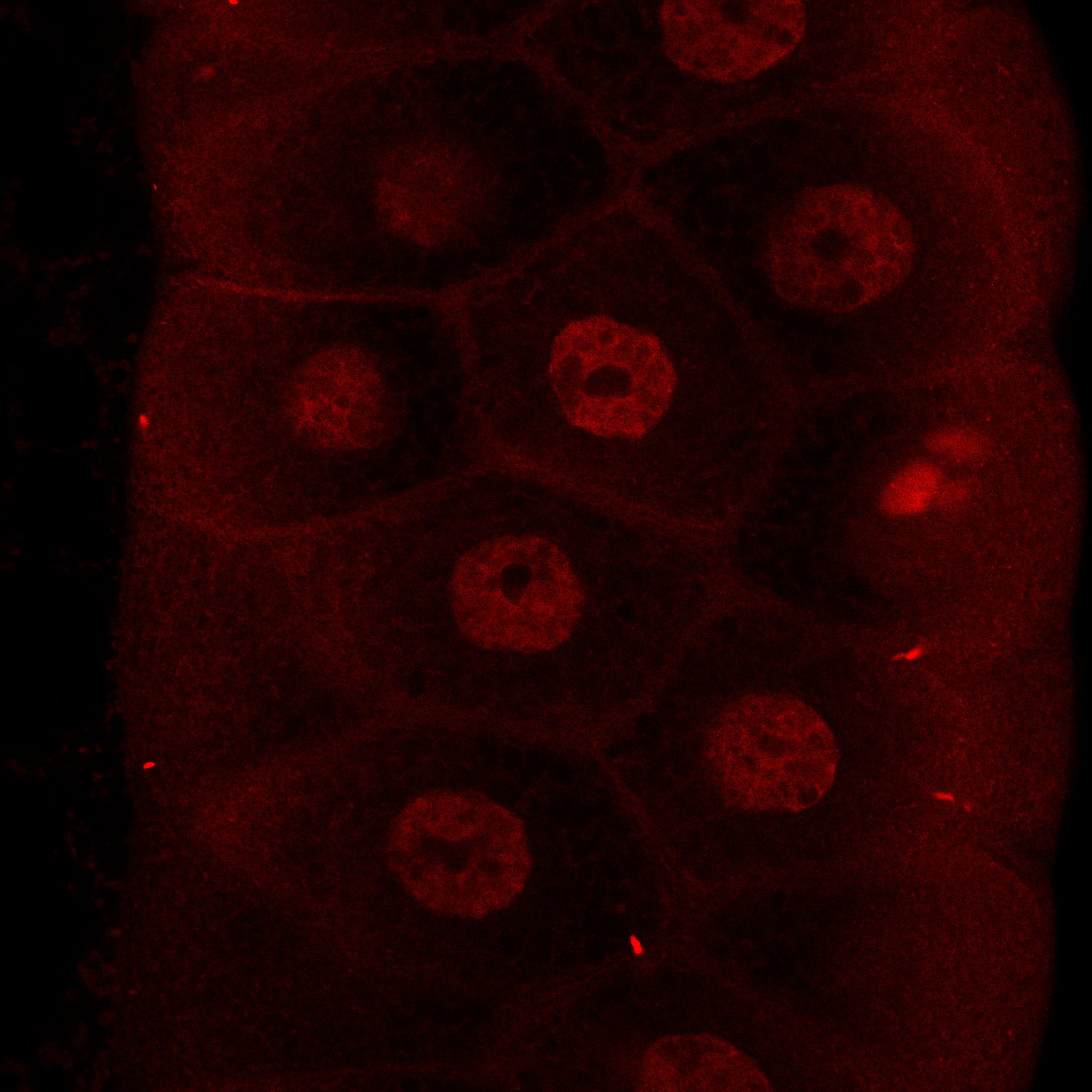

Supplement: Figure 5—source data 2. [file elife-105165-fig5-data2.zip › Figure 5 source data 2/5A_C3-60x_0006.jpg]

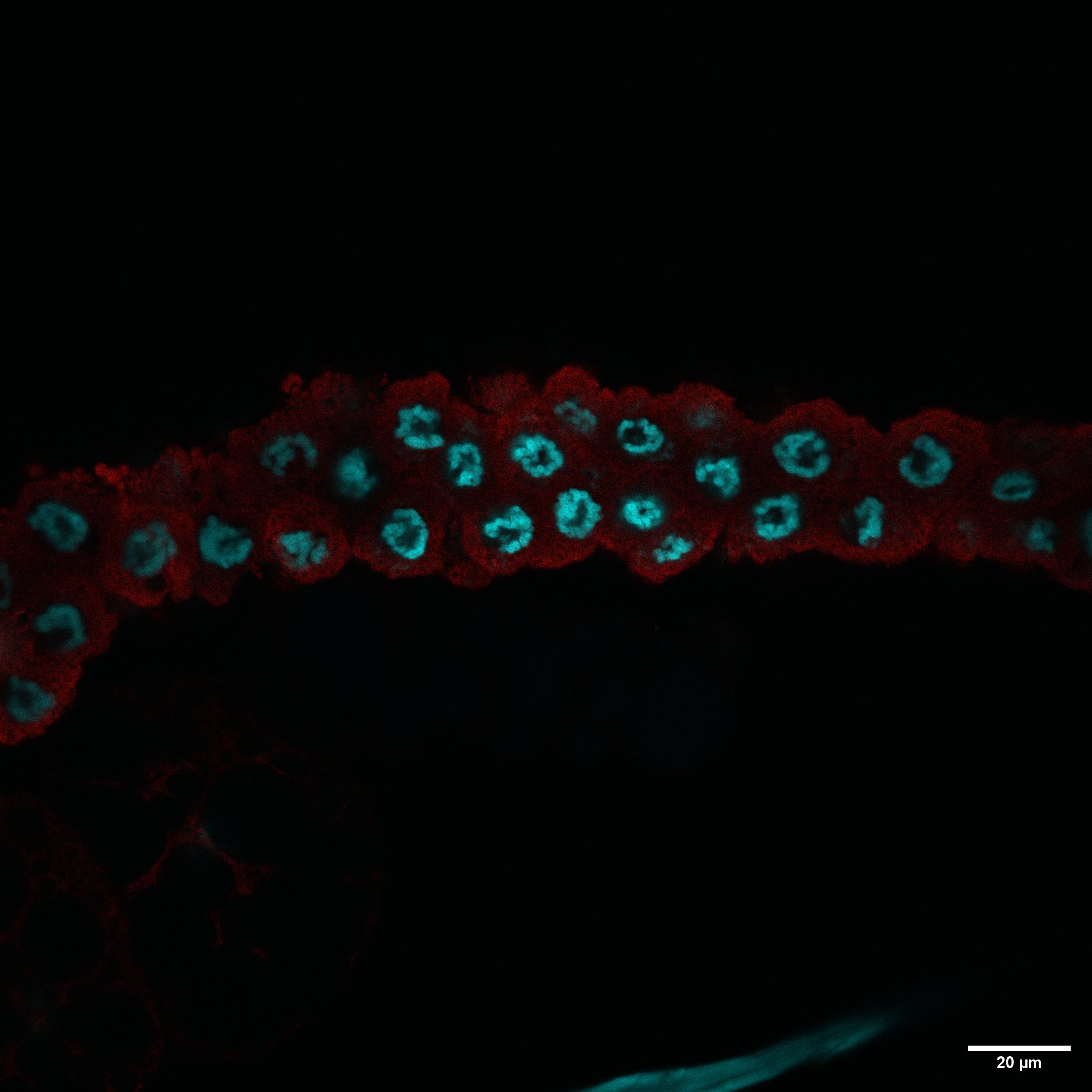

Supplement: Figure 5—figure supplement 1—source data 2. [file elife-105165-fig5-figsupp1-data2.zip › Figure 5- figure supplement 1 Source data 2/S9_A_Act107GD.jpg]

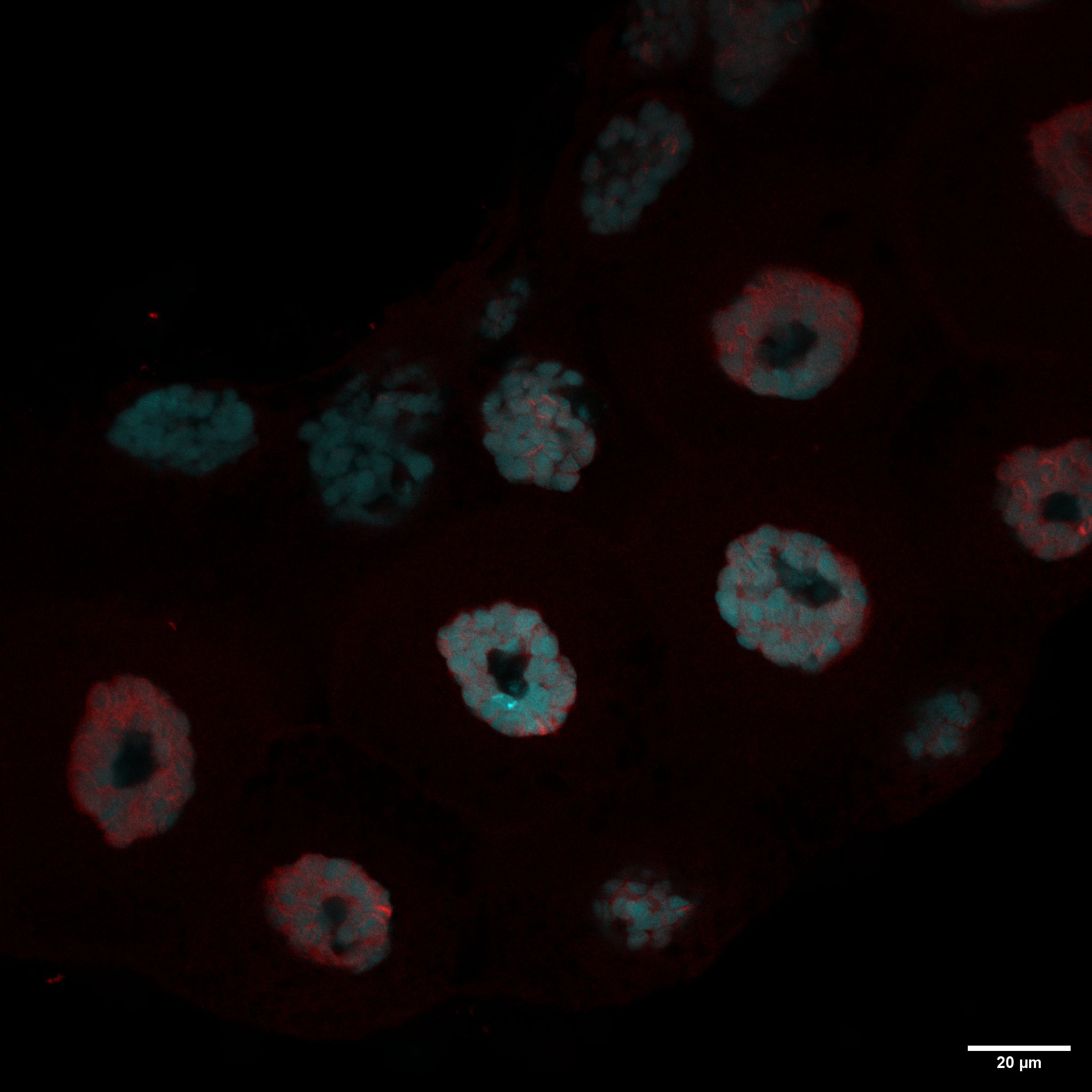

Supplement: Figure 5—figure supplement 1—source data 2. [file elife-105165-fig5-figsupp1-data2.zip › Figure 5- figure supplement 1 Source data 2/S9_B_Phm107GD.jpg]

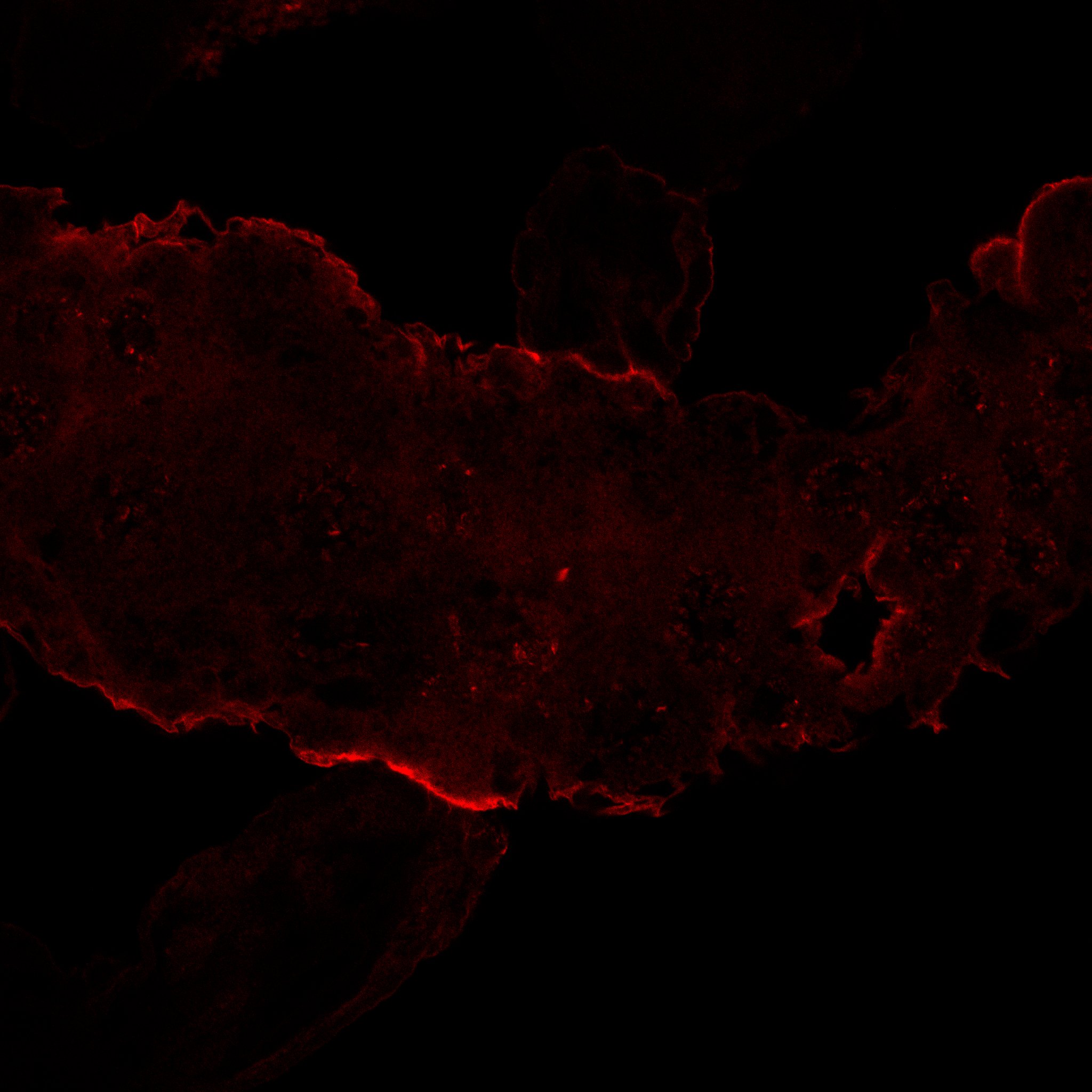

Supplement: Figure 5—figure supplement 1—source data 2. [file elife-105165-fig5-figsupp1-data2.zip › Figure 5- figure supplement 1 Source data 2/S9_A_Phm107GD_C3.jpg]

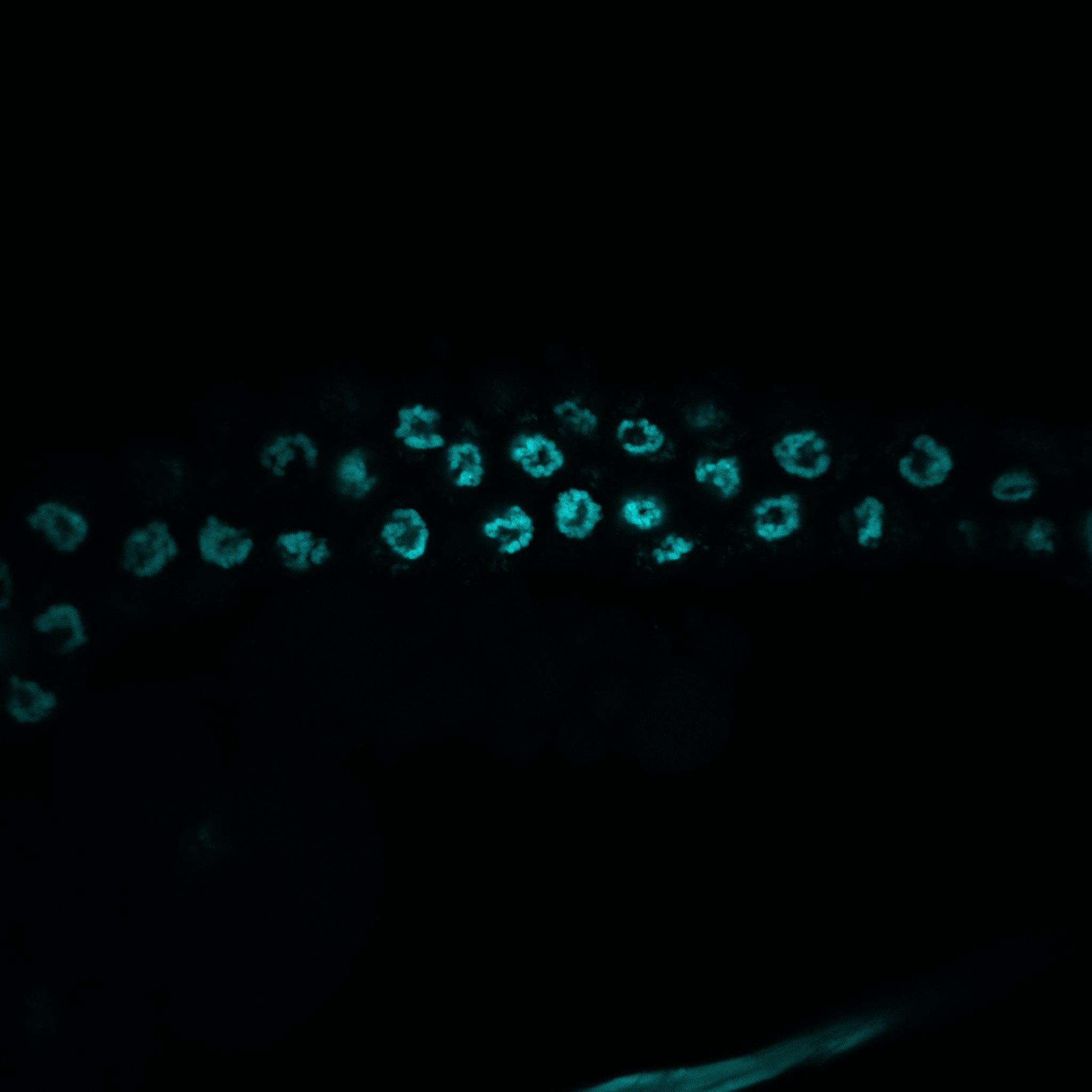

Supplement: Figure 5—figure supplement 1—source data 2. [file elife-105165-fig5-figsupp1-data2.zip › Figure 5- figure supplement 1 Source data 2/S9_A_Act107GD_C1.jpg]

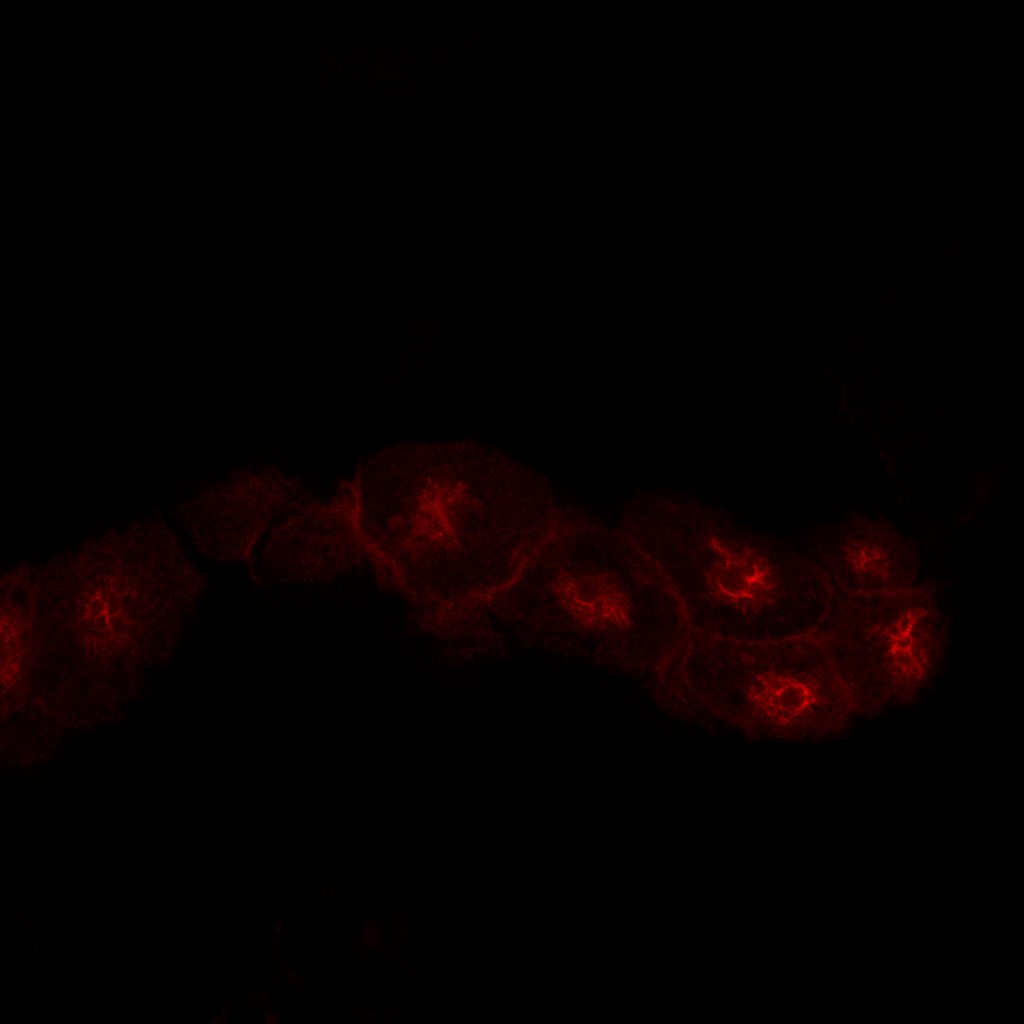

Supplement: Figure 5—figure supplement 1—source data 2. [file elife-105165-fig5-figsupp1-data2.zip › Figure 5- figure supplement 1 Source data 2/S9_B_Act107GD_C3.jpg]

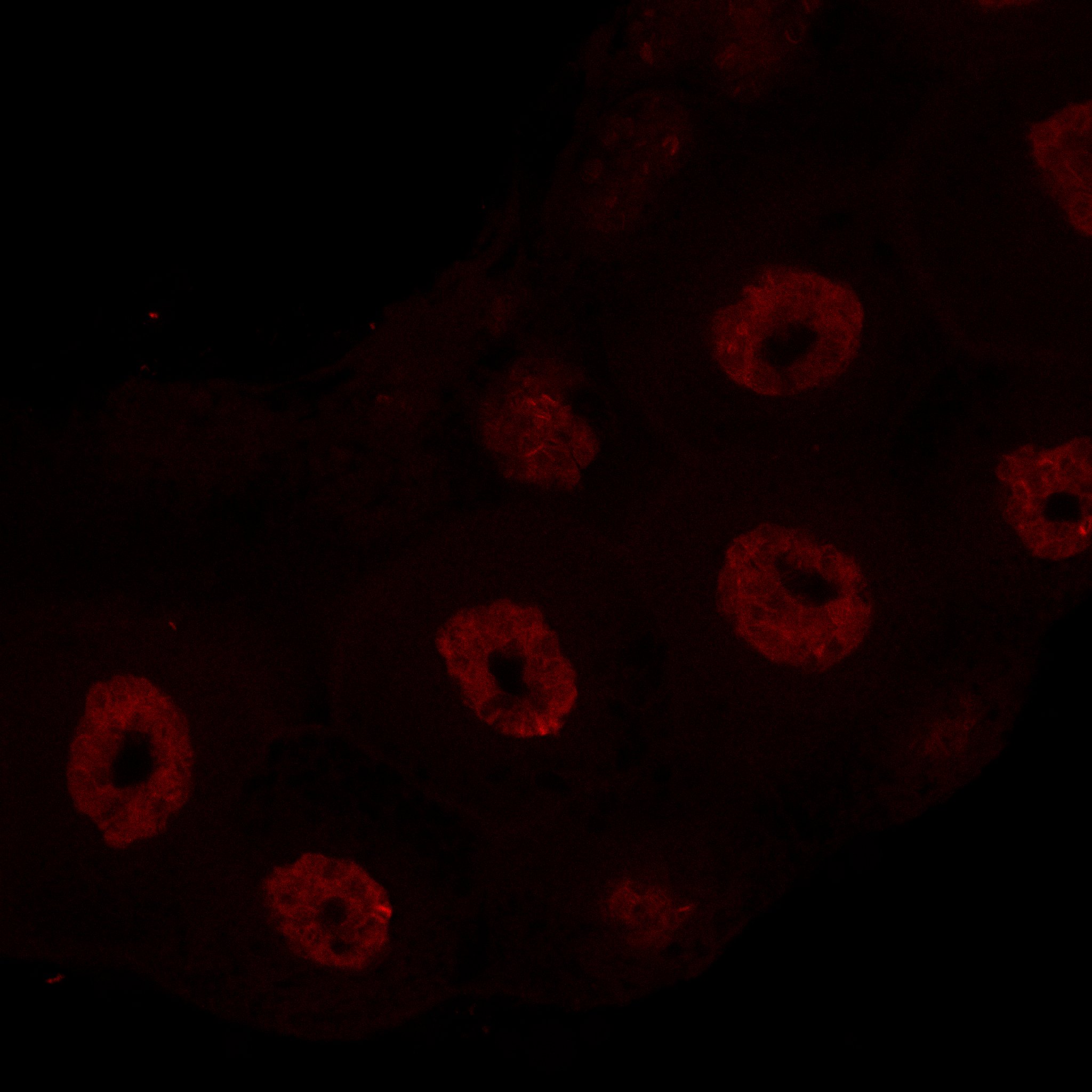

Supplement: Figure 5—figure supplement 1—source data 2. [file elife-105165-fig5-figsupp1-data2.zip › Figure 5- figure supplement 1 Source data 2/S9_B_Phm107GD_C3.jpg]

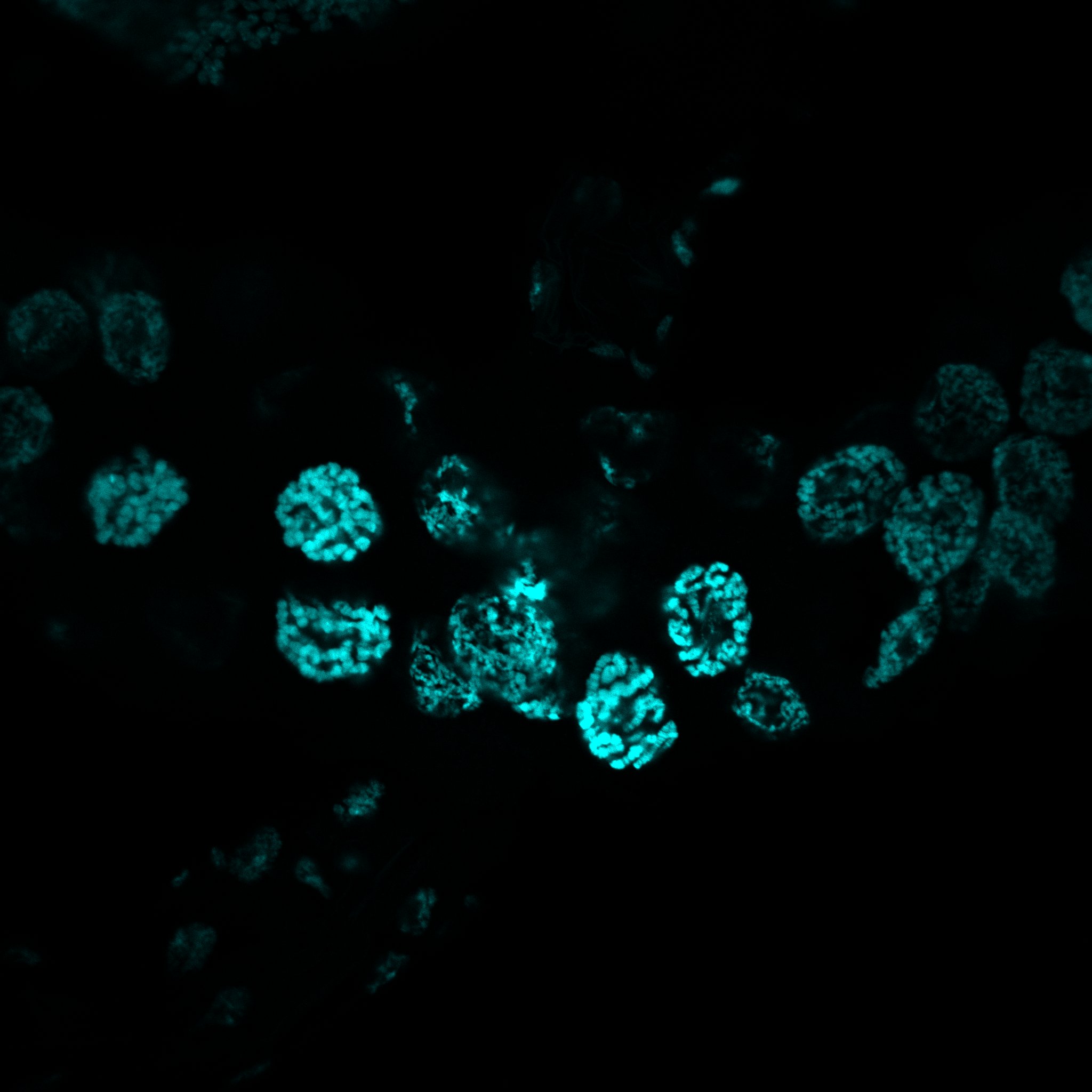

Supplement: Figure 5—figure supplement 1—source data 2. [file elife-105165-fig5-figsupp1-data2.zip › Figure 5- figure supplement 1 Source data 2/S9_A_Phm107GD_C1.jpg]

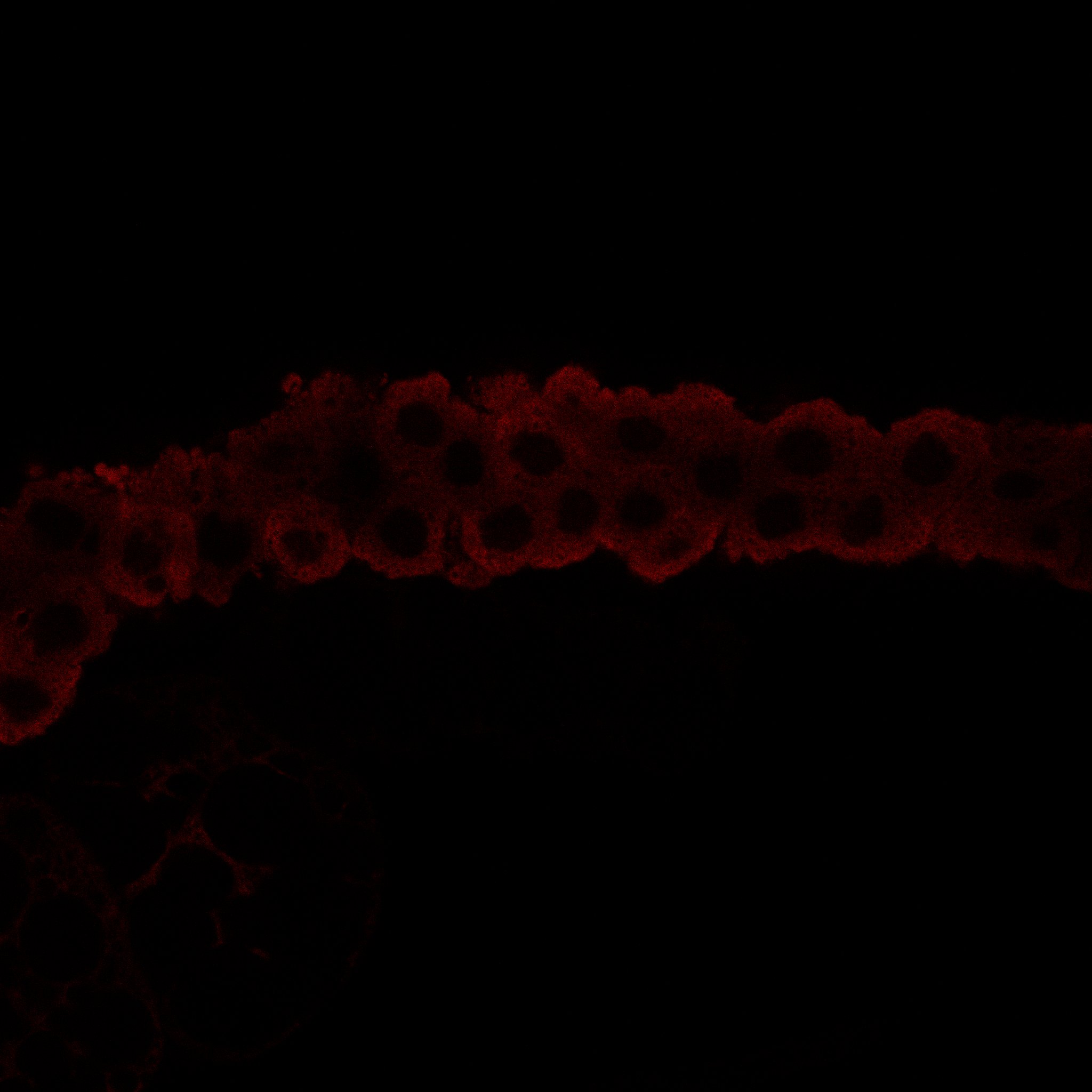

Supplement: Figure 5—figure supplement 1—source data 2. [file elife-105165-fig5-figsupp1-data2.zip › Figure 5- figure supplement 1 Source data 2/S9_A_Act107GD_C3.jpg]

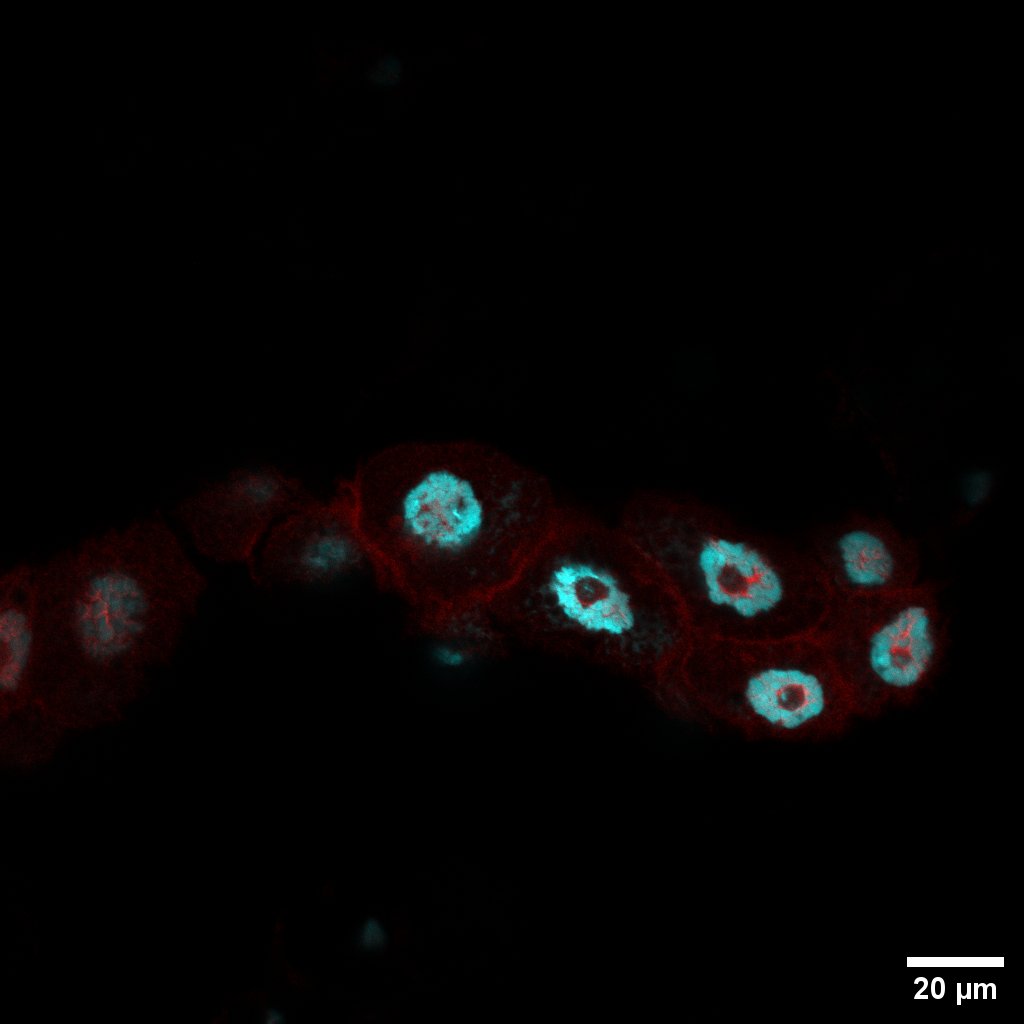

Supplement: Figure 5—figure supplement 1—source data 2. [file elife-105165-fig5-figsupp1-data2.zip › Figure 5- figure supplement 1 Source data 2/S9_B_Act107GD.jpg]

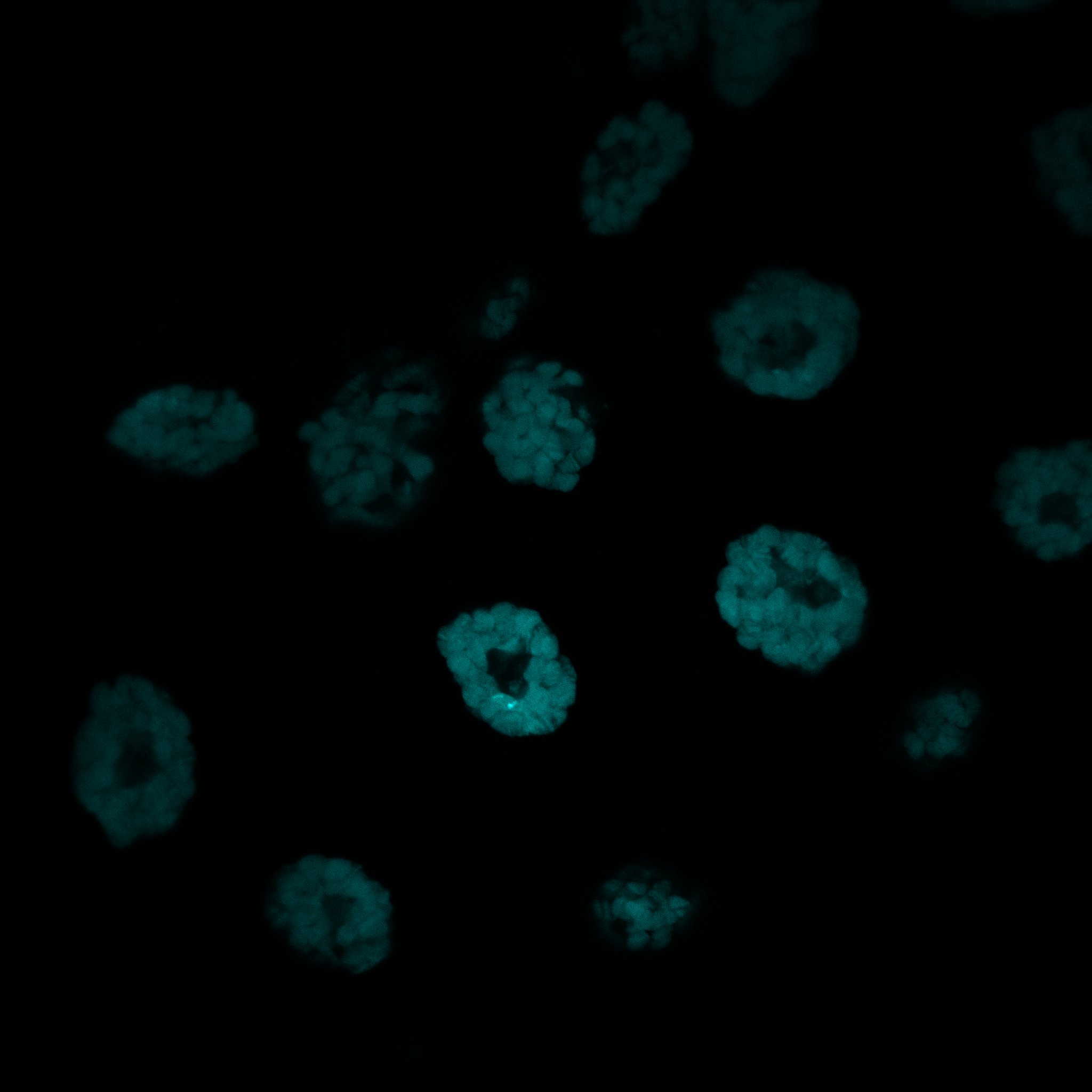

Supplement: Figure 5—figure supplement 1—source data 2. [file elife-105165-fig5-figsupp1-data2.zip › Figure 5- figure supplement 1 Source data 2/S9_B_Phm107GD_C1.jpg]

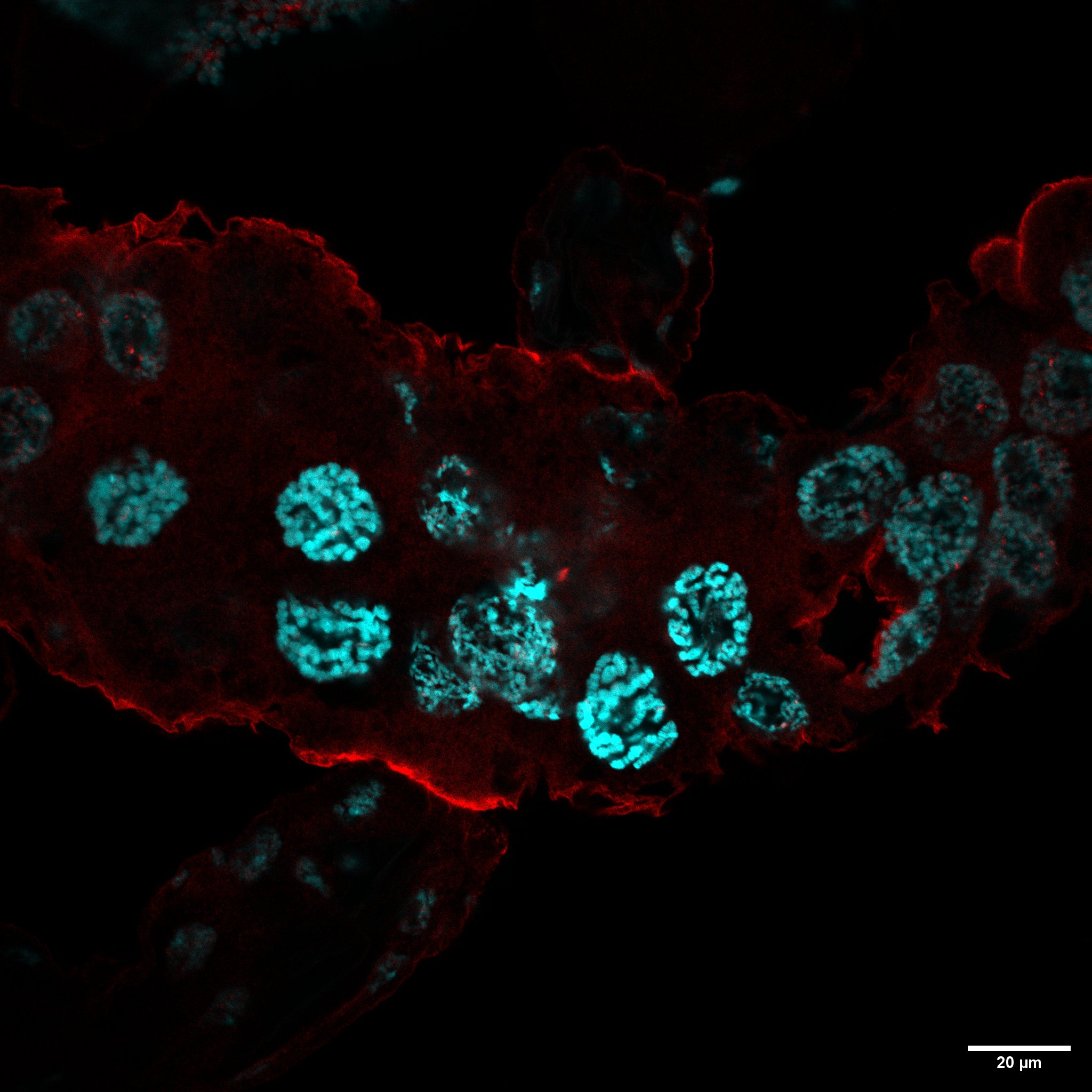

Supplement: Figure 5—figure supplement 1—source data 2. [file elife-105165-fig5-figsupp1-data2.zip › Figure 5- figure supplement 1 Source data 2/S9_A_Phm107GD.jpg]

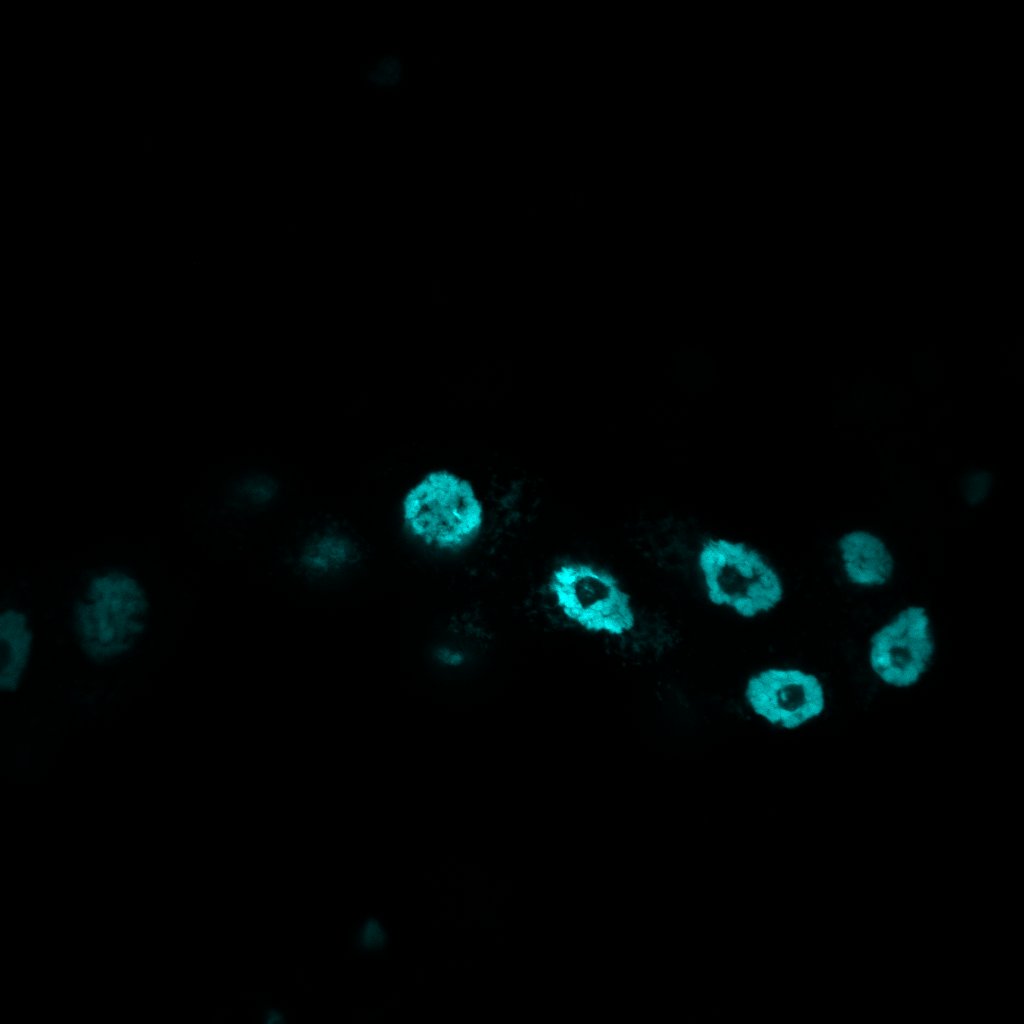

Supplement: Figure 5—figure supplement 1—source data 2. [file elife-105165-fig5-figsupp1-data2.zip › Figure 5- figure supplement 1 Source data 2/S9_B_Act107GD_C1.jpg]

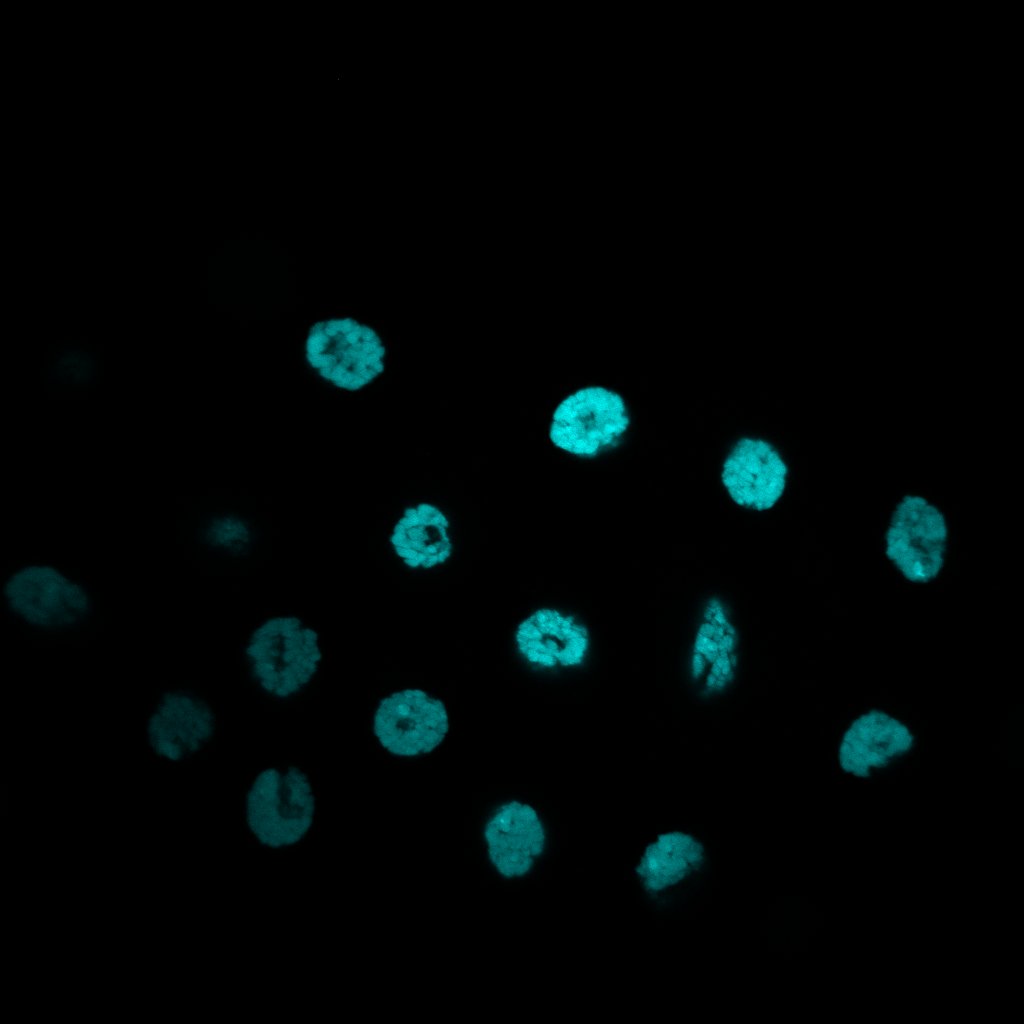

Supplement: Figure 6—source data 2. [file elife-105165-fig6-data2.zip › Figure 6 source data 2/6G_C1.jpg]

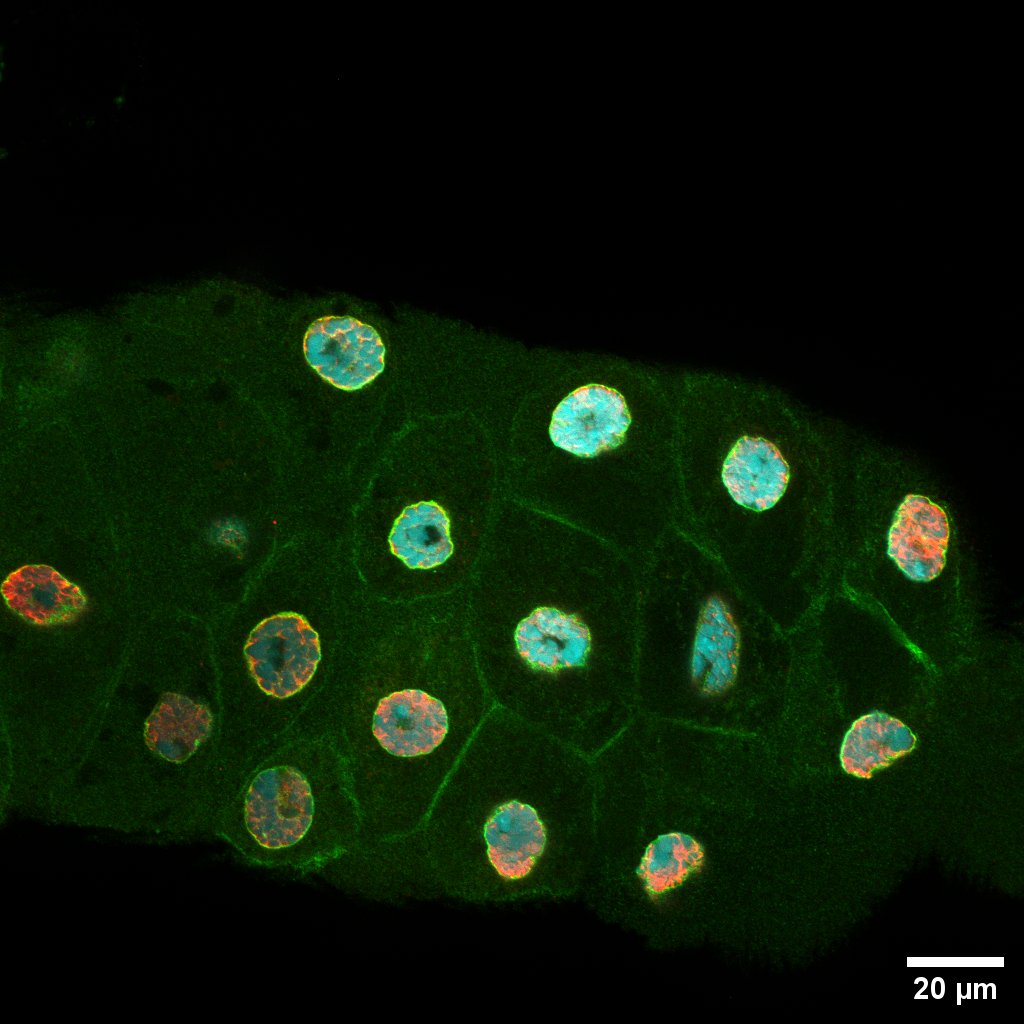

Supplement: Figure 6—source data 2. [file elife-105165-fig6-data2.zip › Figure 6 source data 2/6G_.jpg]

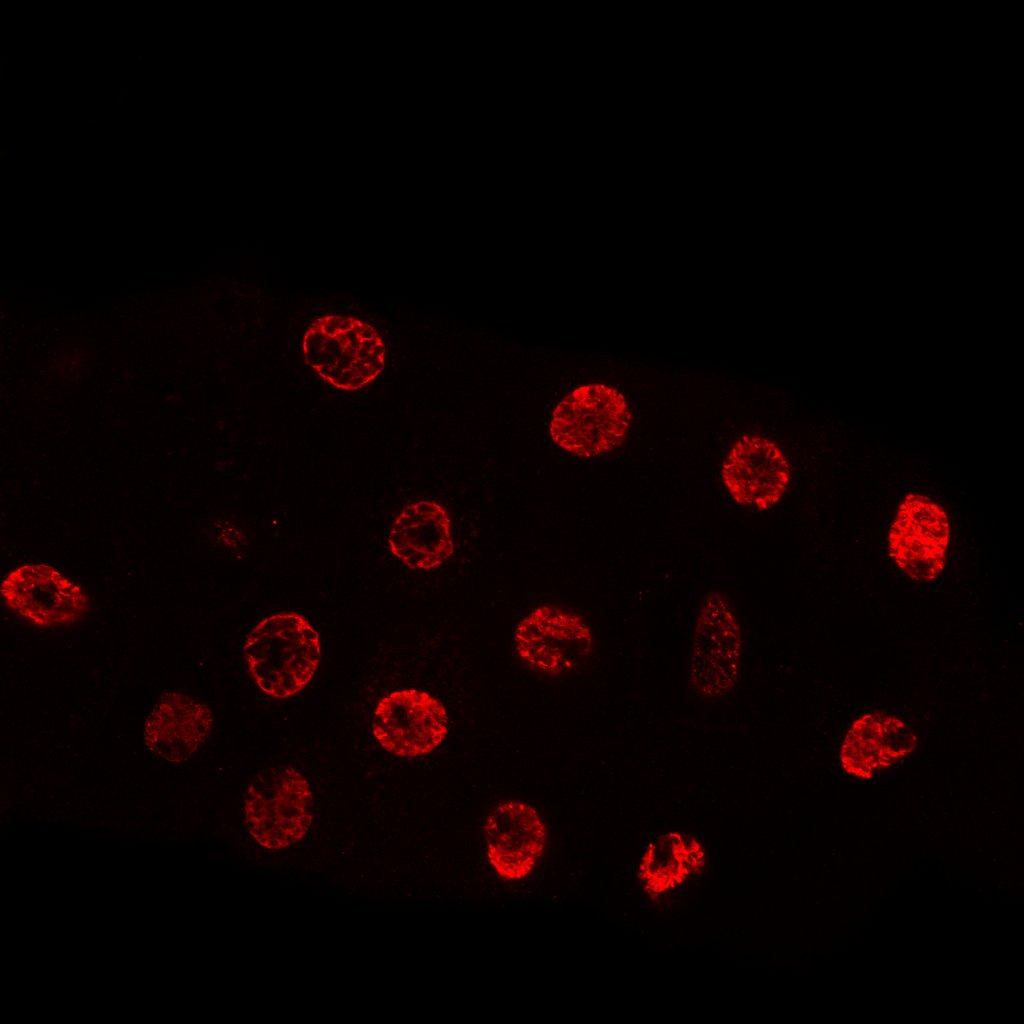

Supplement: Figure 6—source data 2. [file elife-105165-fig6-data2.zip › Figure 6 source data 2/6G_C3.jpg]

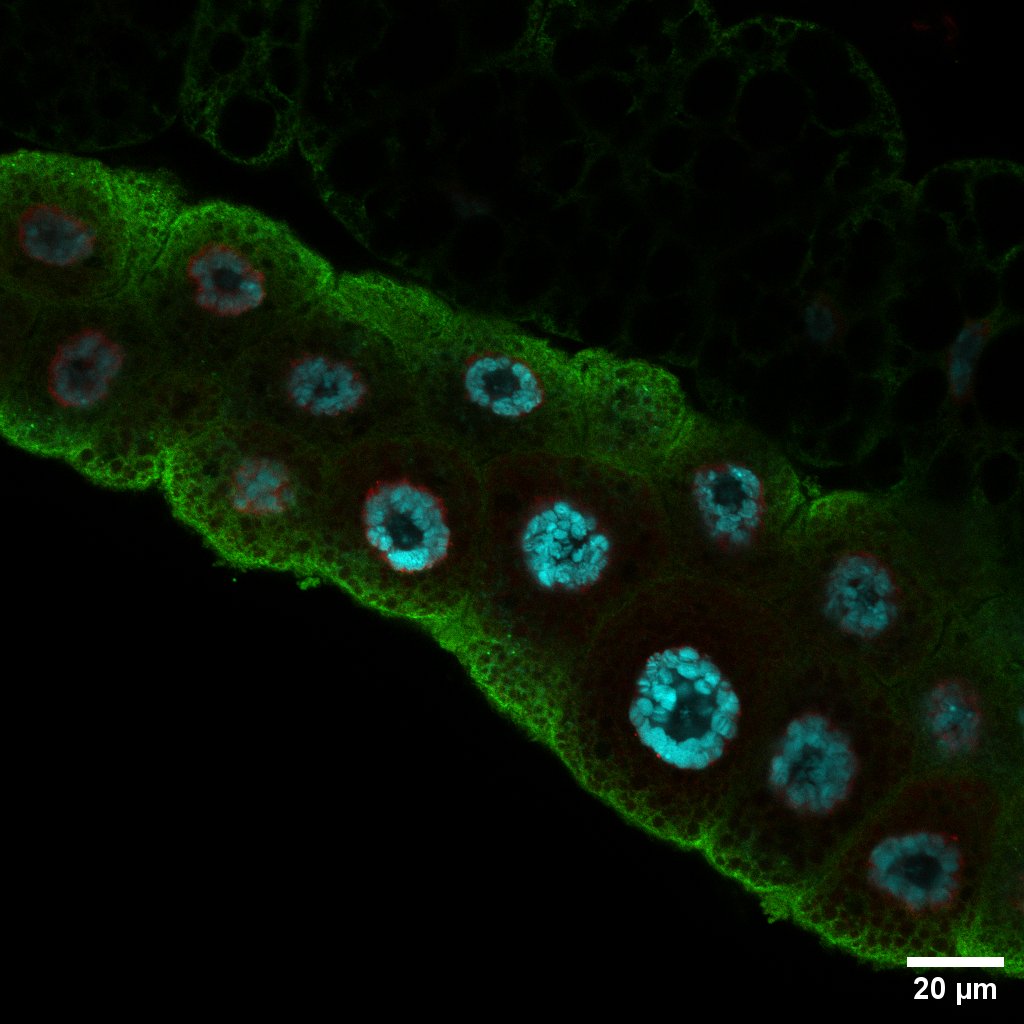

Supplement: Figure 6—source data 2. [file elife-105165-fig6-data2.zip › Figure 6 source data 2/6F_.jpg]

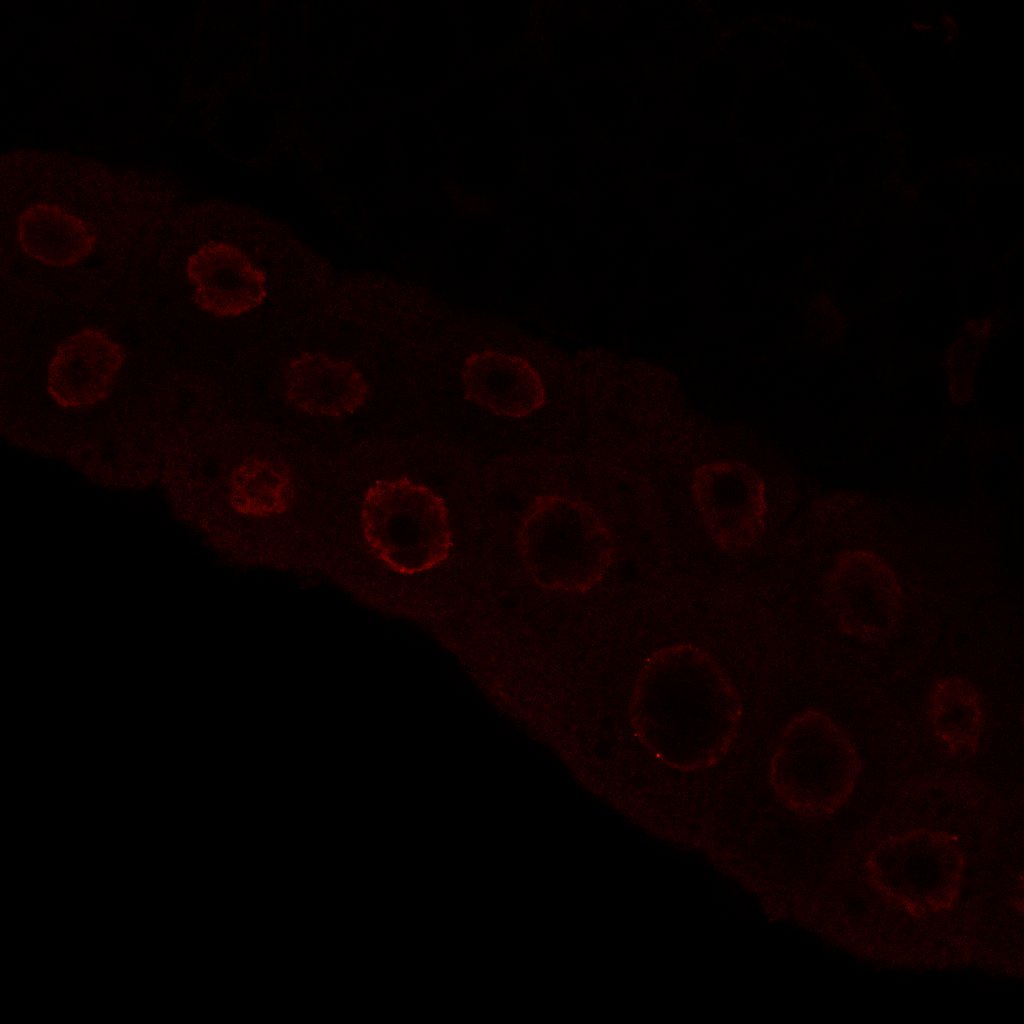

Supplement: Figure 6—source data 2. [file elife-105165-fig6-data2.zip › Figure 6 source data 2/6F_C3.jpg]

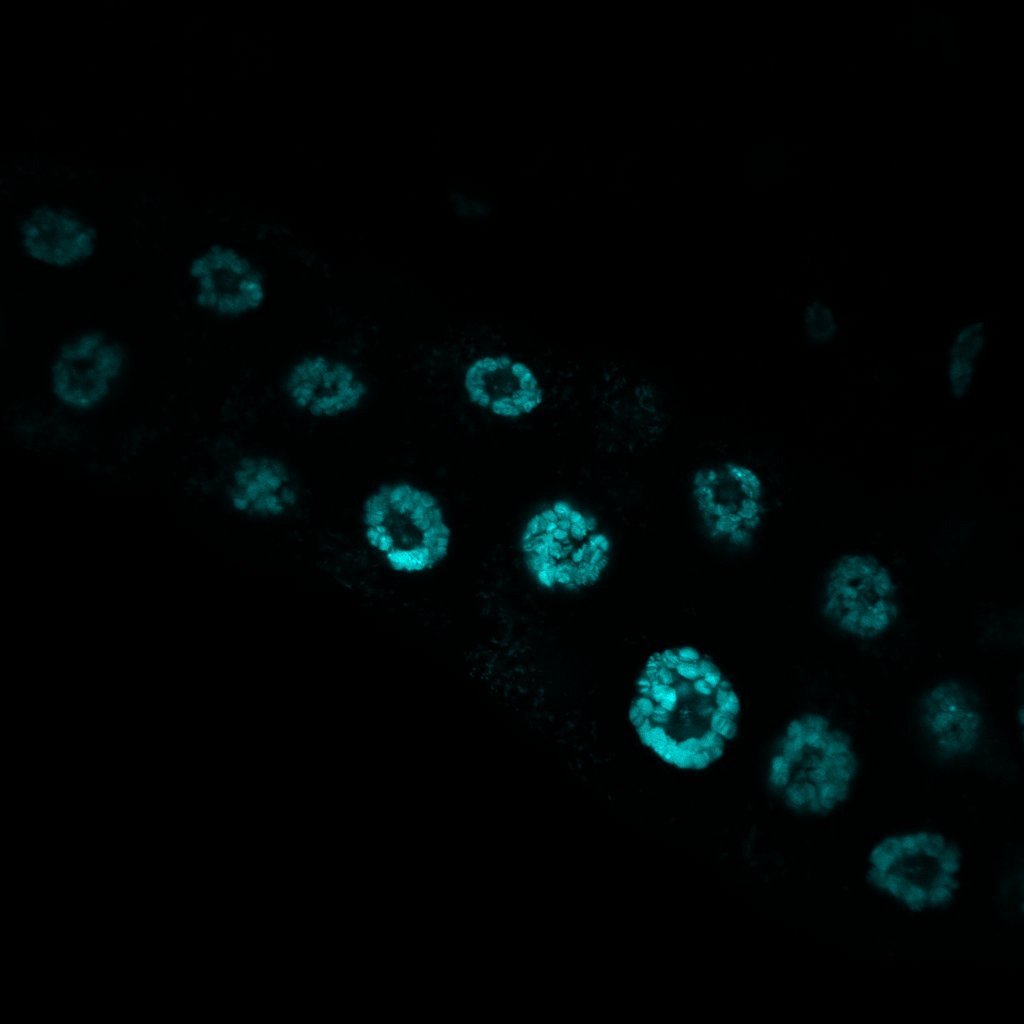

Supplement: Figure 6—source data 2. [file elife-105165-fig6-data2.zip › Figure 6 source data 2/6F_C1.jpg]

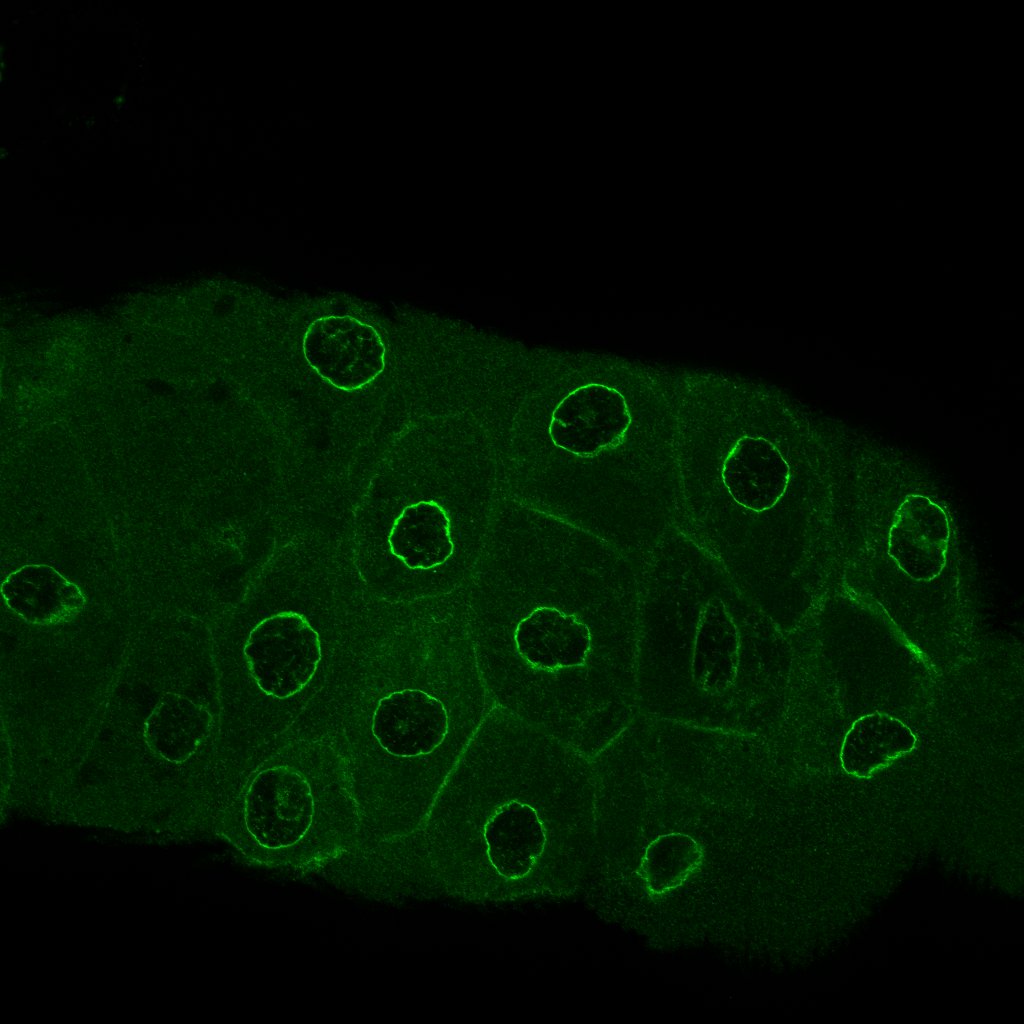

Supplement: Figure 6—source data 2. [file elife-105165-fig6-data2.zip › Figure 6 source data 2/6G_C2.jpg]

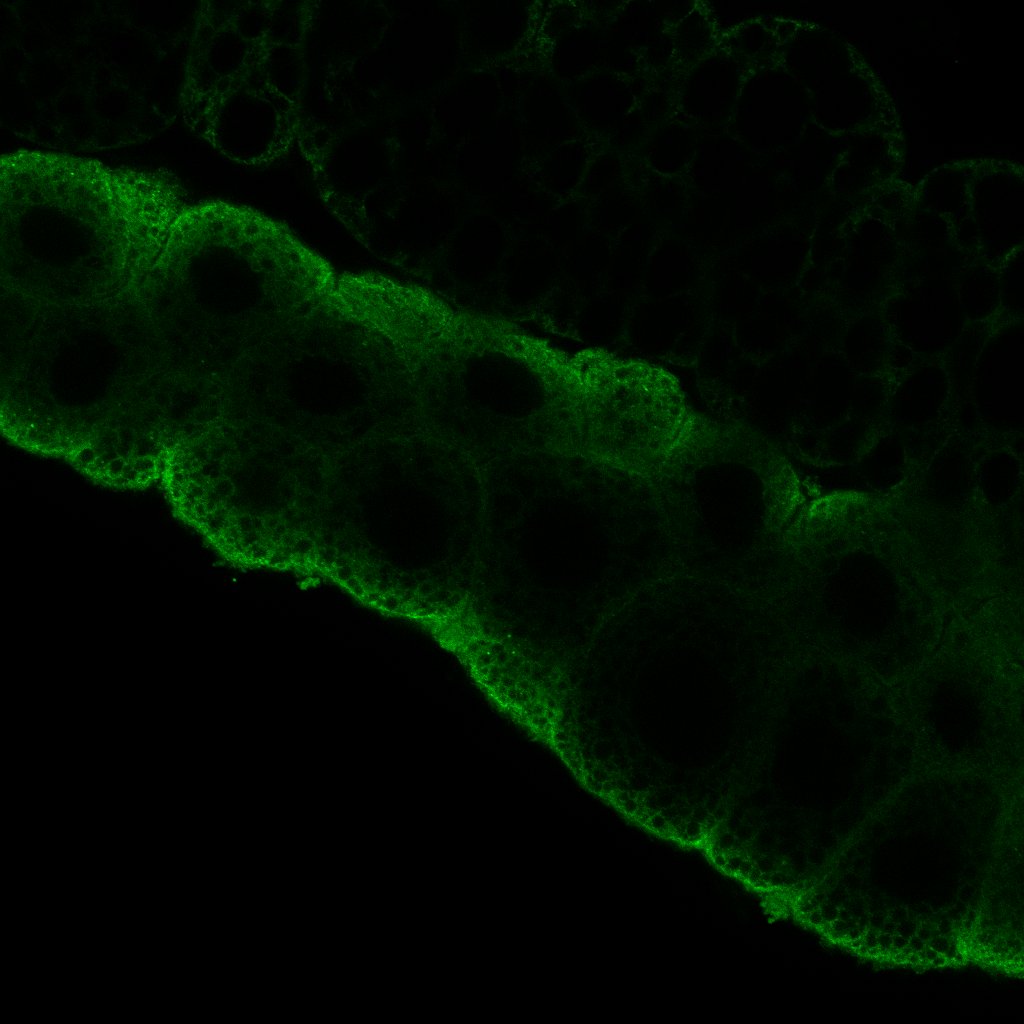

Supplement: Figure 6—source data 2. [file elife-105165-fig6-data2.zip › Figure 6 source data 2/6F_C2.jpg]

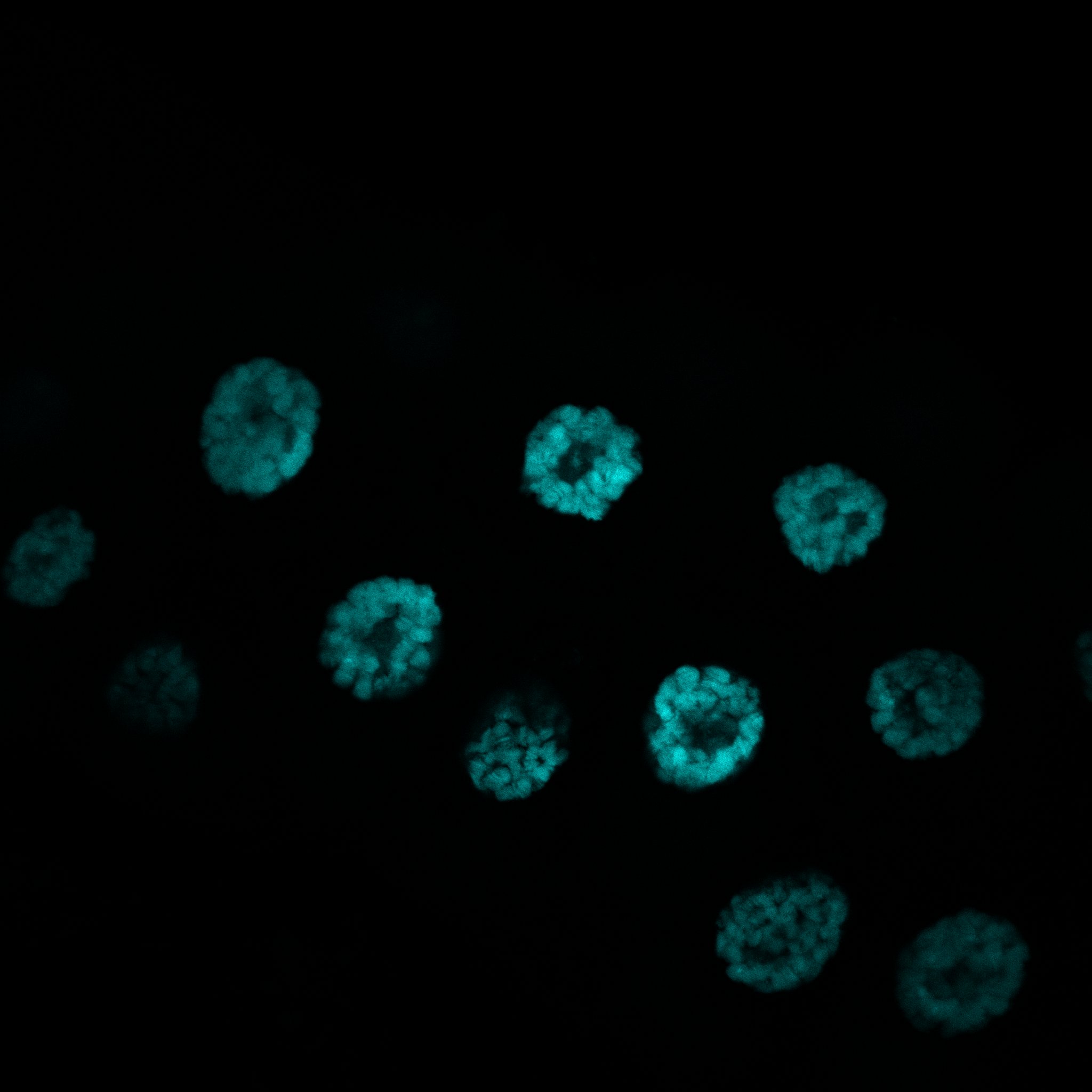

Supplement: Figure 6—source data 2. [file elife-105165-fig6-data2.zip › Figure 6 source data 2/6E_C1.jpg]

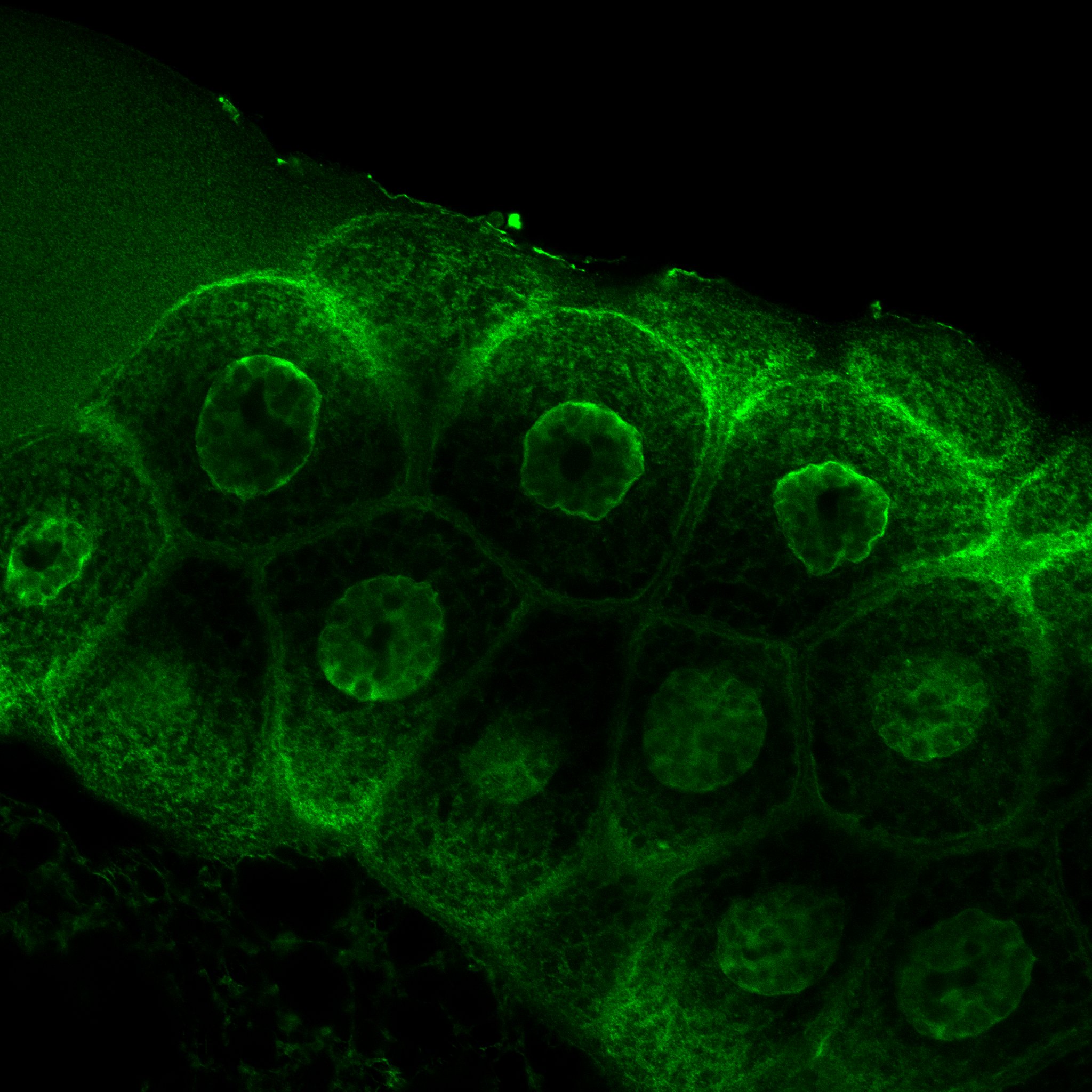

Supplement: Figure 6—source data 2. [file elife-105165-fig6-data2.zip › Figure 6 source data 2/6E_C2.jpg]

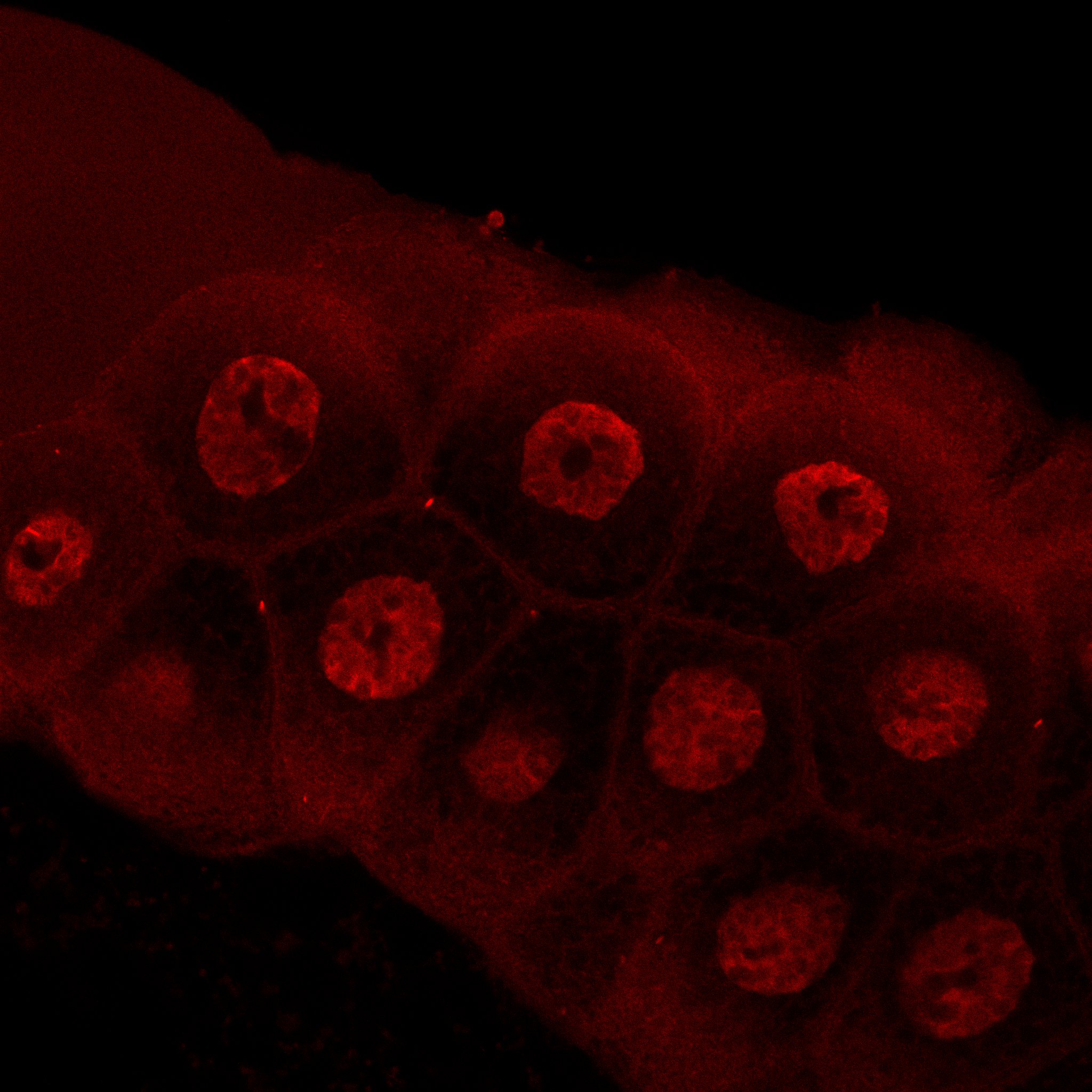

Supplement: Figure 6—source data 2. [file elife-105165-fig6-data2.zip › Figure 6 source data 2/6E_C3.jpg]

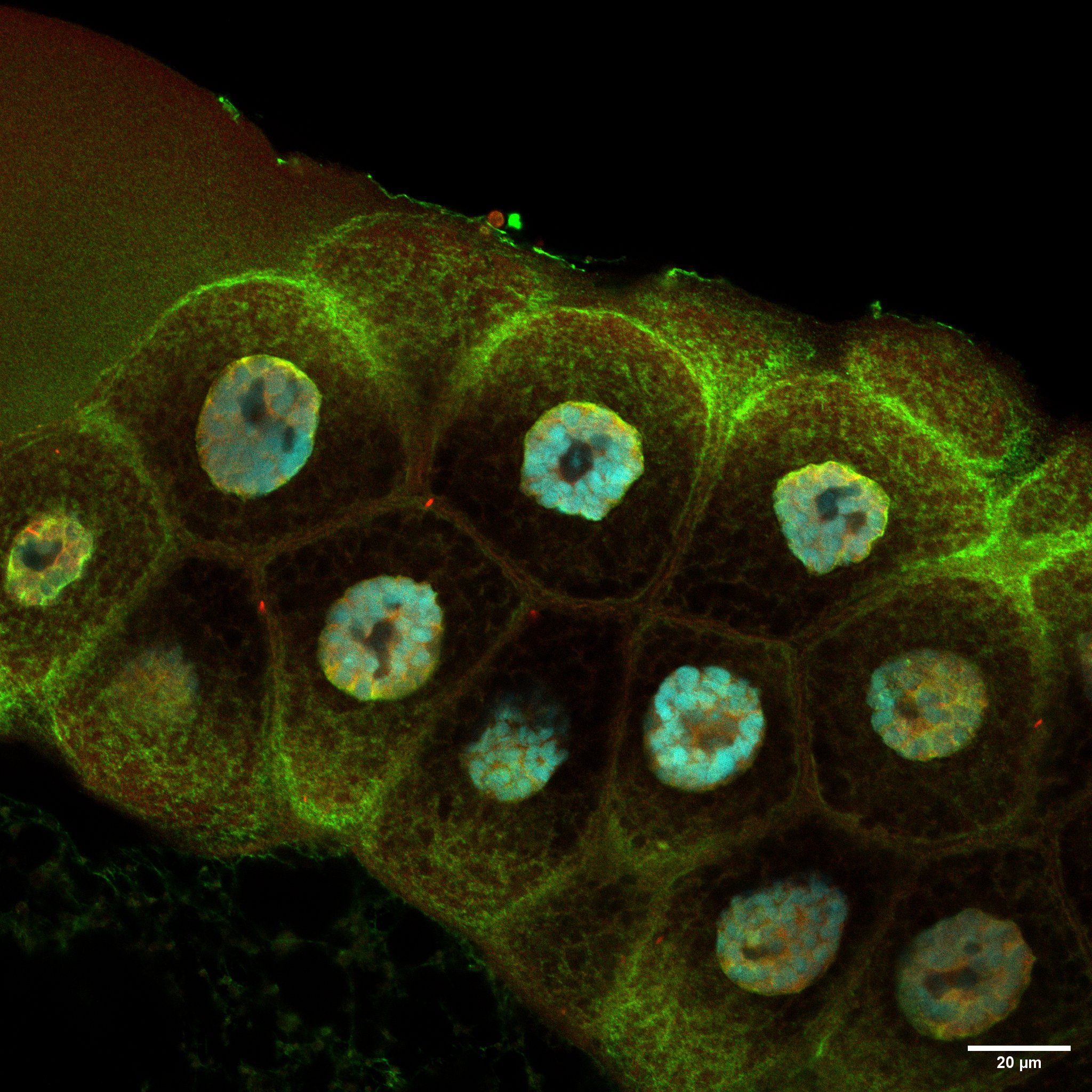

Supplement: Figure 6—source data 2. [file elife-105165-fig6-data2.zip › Figure 6 source data 2/6E_.jpg]

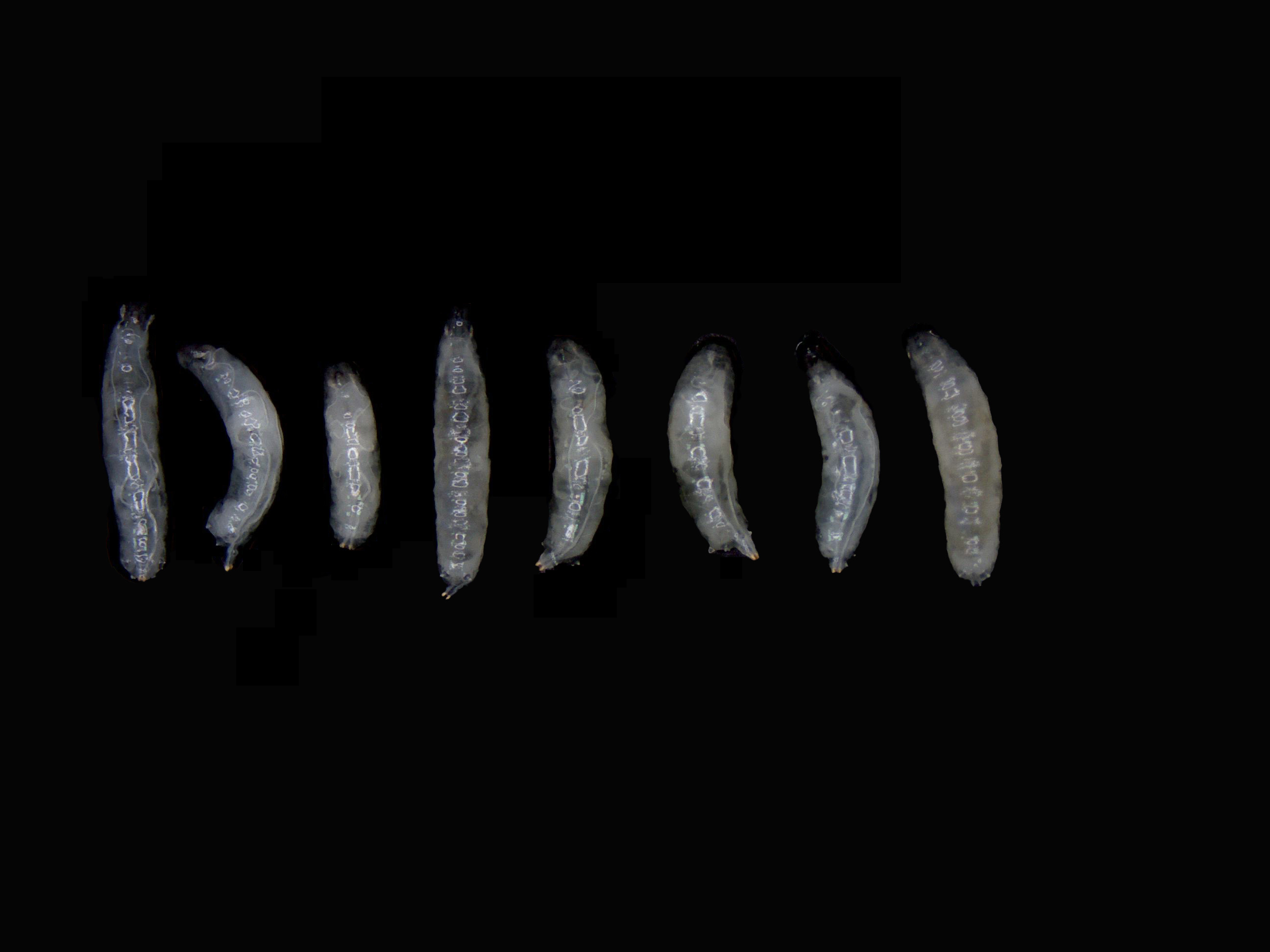

Supplement: Figure 6—figure supplement 1—source data 2. [file elife-105165-fig6-figsupp1-data2.zip › Figure 6- figure supplement 1 Source data 2/S10_A.jpg]
