# Supplementary material for: Nup107 is a crucial regulator of torso-mediated metamorphic transition in Drosophila melanogaster
Source: eLife. 2026 Mar 10;14:RP105165. doi: 10.7554/eLife.105165 (PMC12975125; doi:10.7554/eLife.105165)
Supplement: Figure 1—figure supplement 1—source data 1. — The cells highlighted in the yellow box were included in the supplementary figure. The upper panel corresponds to mAb414 staining, while the lower panel corresponds to mRFP-Nup107. [file elife-105165-fig1-figsupp1-data1.pdf]

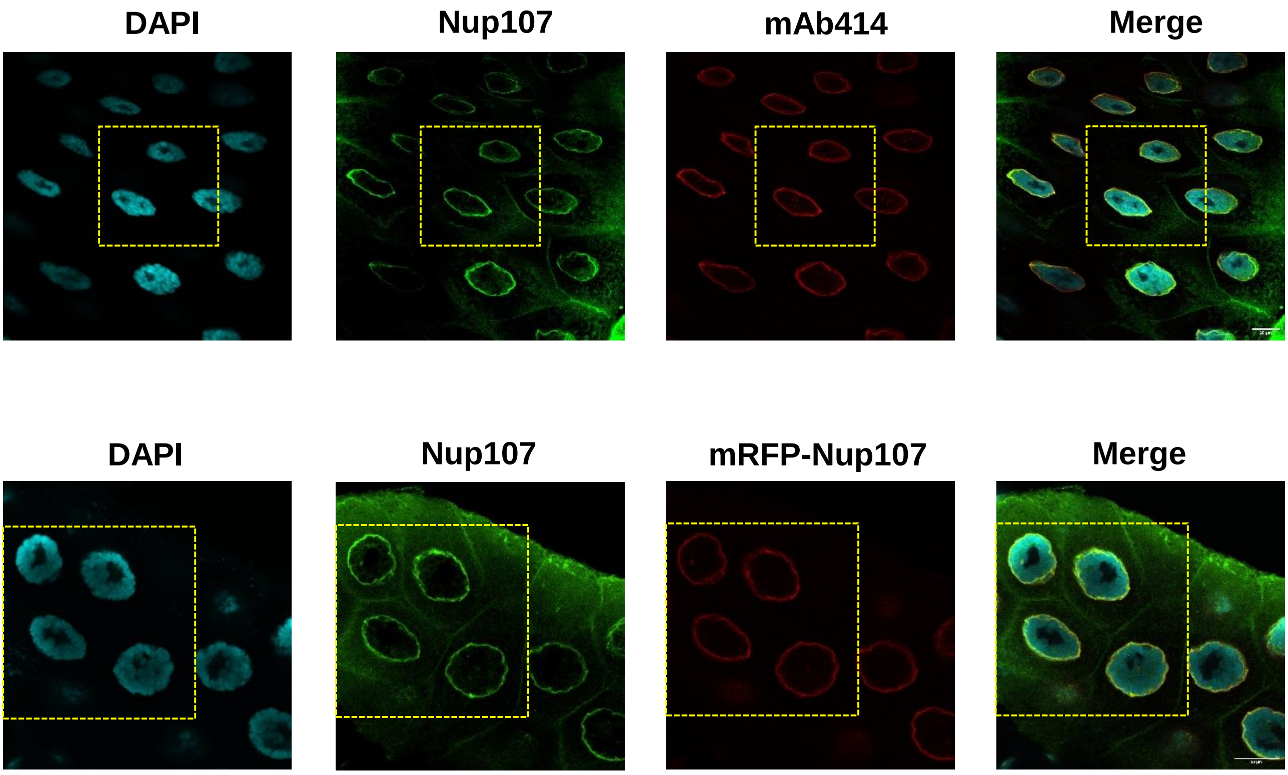


**Figure 1- figure supplement 1, Source Data 1.** Original confocal images are presented for Figure 1- figure Supplement 1. The cells highlighted in the yellow box were included in the supplementary figure. The upper panel corresponds to mAb414 staining, while the lower panel corresponds to mRFP-Nup107.
