# Supplementary material for: Nup107 is a crucial regulator of torso-mediated metamorphic transition in Drosophila melanogaster
Source: eLife. 2026 Mar 10;14:RP105165. doi: 10.7554/eLife.105165 (PMC12975125; doi:10.7554/eLife.105165)
Supplement: Figure 1—figure supplement 2—source data 1. — The first lane displays wild-type samples (+/+), while the second lane shows a heterozygous sample (+/-), which has one copy of Nup107 deleted. The third lane contains the DNA ladder. [file elife-105165-fig1-figsupp2-data1.zip › Figure 1-figure supplement 2_Source data 1/Figure 1-figure supplement 2.pdf]

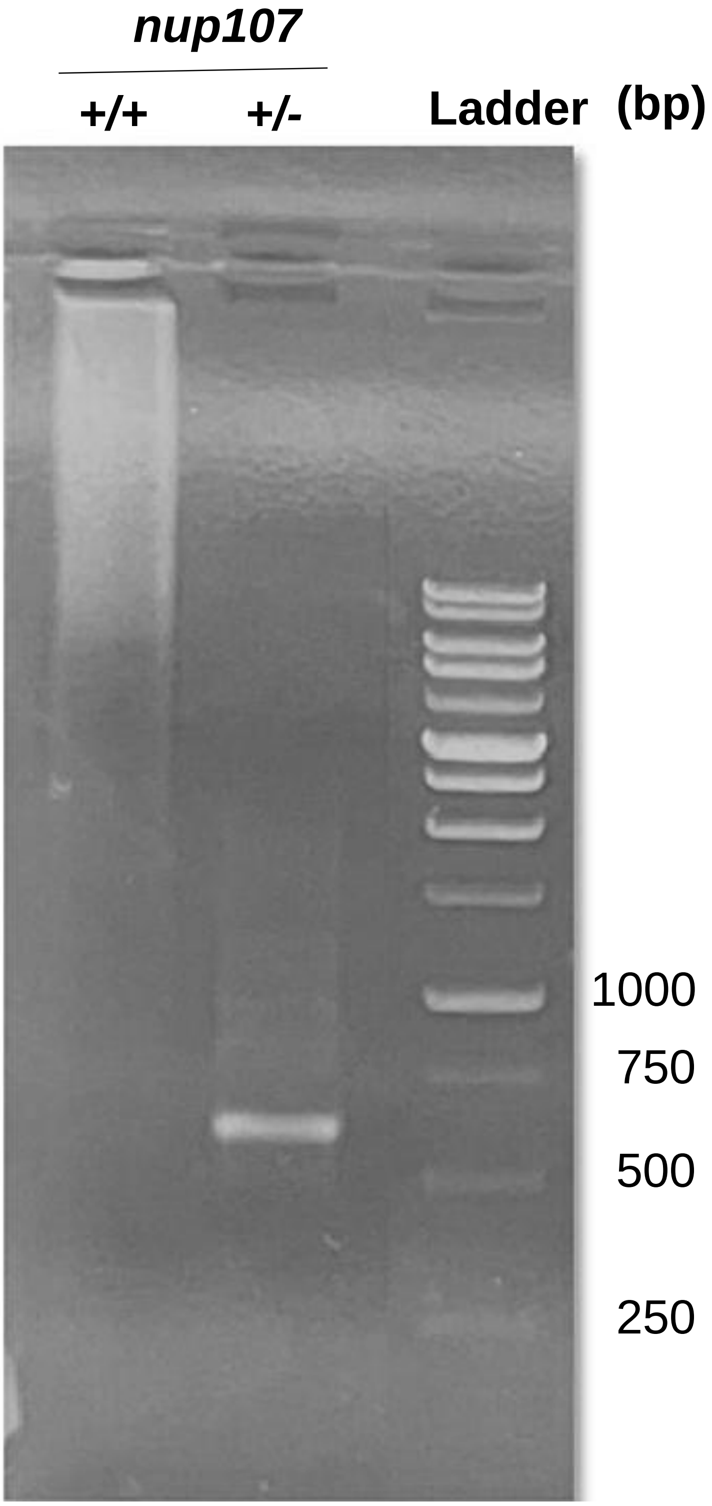

Figure 1- figure supplement 2, Source Data 1. The original DNA gel image corresponds to Figure 1 - Supplement 2B. The first lane displays wild-type samples (+/+), while the second lane shows a heterozygous sample (+/-), which has one copy of Nup107 deleted. The third lane contains the DNA ladder.
