# Supplementary material for: Nup107 is a crucial regulator of torso-mediated metamorphic transition in Drosophila melanogaster
Source: eLife. 2026 Mar 10;14:RP105165. doi: 10.7554/eLife.105165 (PMC12975125; doi:10.7554/eLife.105165)
Supplement: Figure 2—source data 1. [file elife-105165-fig2-data1.zip › Figure 2 source data 1/Figure 2 with 2 supplement.pdf]

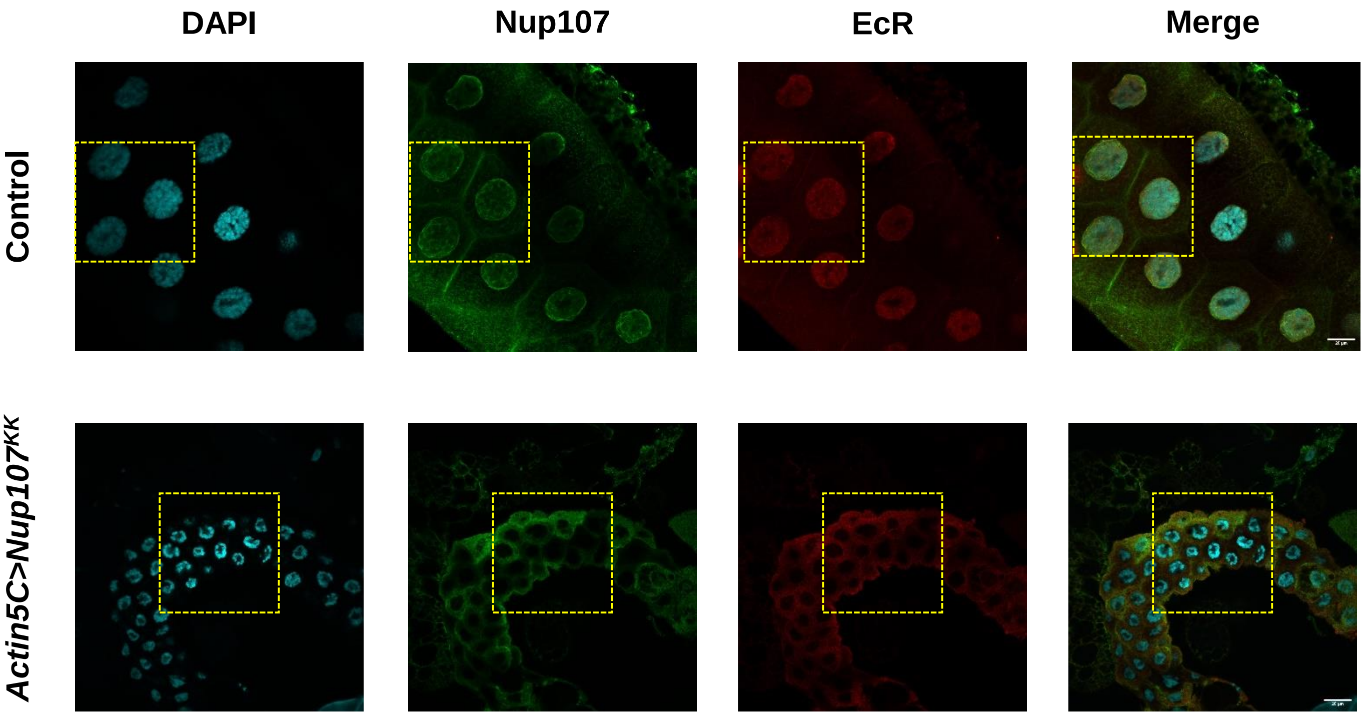

Figure 2, Source Data 1. Original images for Figures 2A and 2B are shown. The cells highlighted in the yellow box were included in the main figure.
