# Supplementary material for: Nup107 is a crucial regulator of torso-mediated metamorphic transition in Drosophila melanogaster
Source: eLife. 2026 Mar 10;14:RP105165. doi: 10.7554/eLife.105165 (PMC12975125; doi:10.7554/eLife.105165)
Supplement: Figure 2—figure supplement 1—source data 1. — The upper panel corresponds to salivary gland images, while the lower panel corresponds to brain complex images. [file elife-105165-fig2-figsupp1-data1.pdf]

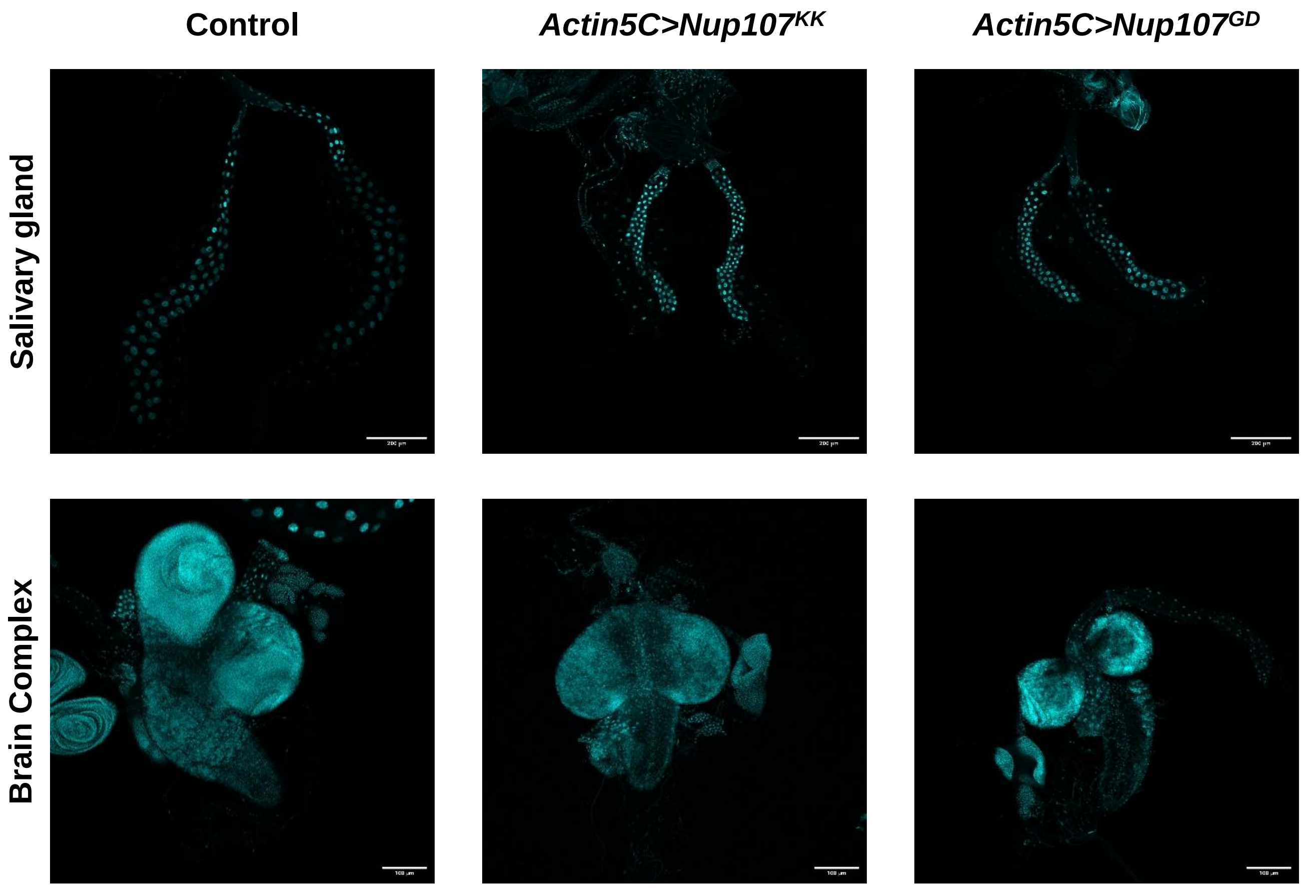

Figure 2- figure supplement 1, Source Data 1. Original confocal images are presented for Figure 1- figure Supplement 1. The upper panel corresponds to Salivary gland images, while the lower panel corresponds to Brain complex images.
