# Supplementary material for: Nup107 is a crucial regulator of torso-mediated metamorphic transition in Drosophila melanogaster
Source: eLife. 2026 Mar 10;14:RP105165. doi: 10.7554/eLife.105165 (PMC12975125; doi:10.7554/eLife.105165)
Supplement: Figure 2—figure supplement 2—source data 1. — The cells highlighted in the yellow box were included in the supplementary figure. [file elife-105165-fig2-figsupp2-data1.pdf]

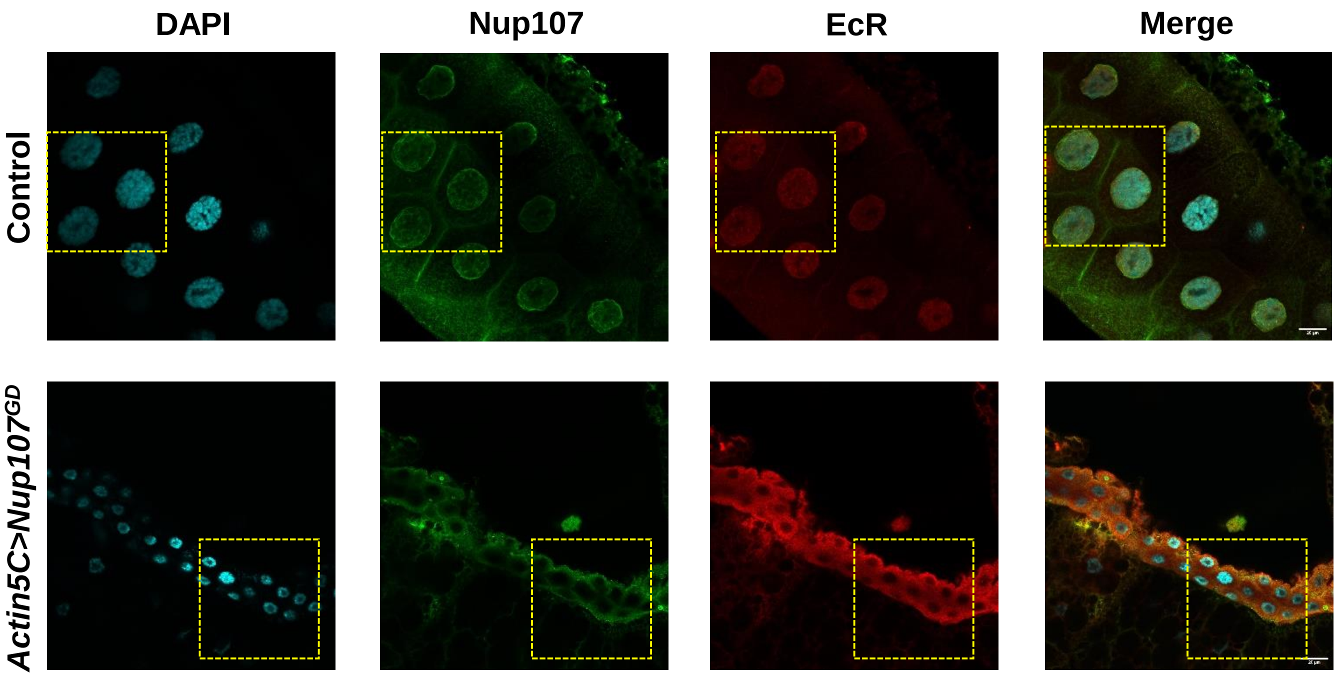


**Figure 2- figure supplement 2, Source Data 1.** Original images for Figure 2- figure supplement 2 are shown. The cells highlighted in the yellow box were included in the supplementary figure.
