# Supplementary material for: Nup107 is a crucial regulator of torso-mediated metamorphic transition in Drosophila melanogaster
Source: eLife. 2026 Mar 10;14:RP105165. doi: 10.7554/eLife.105165 (PMC12975125; doi:10.7554/eLife.105165)
Supplement: Figure 3—source data 1. [file elife-105165-fig3-data1.zip › Figure 3 source data 1/Figure 3 with 3 supplement.pdf]

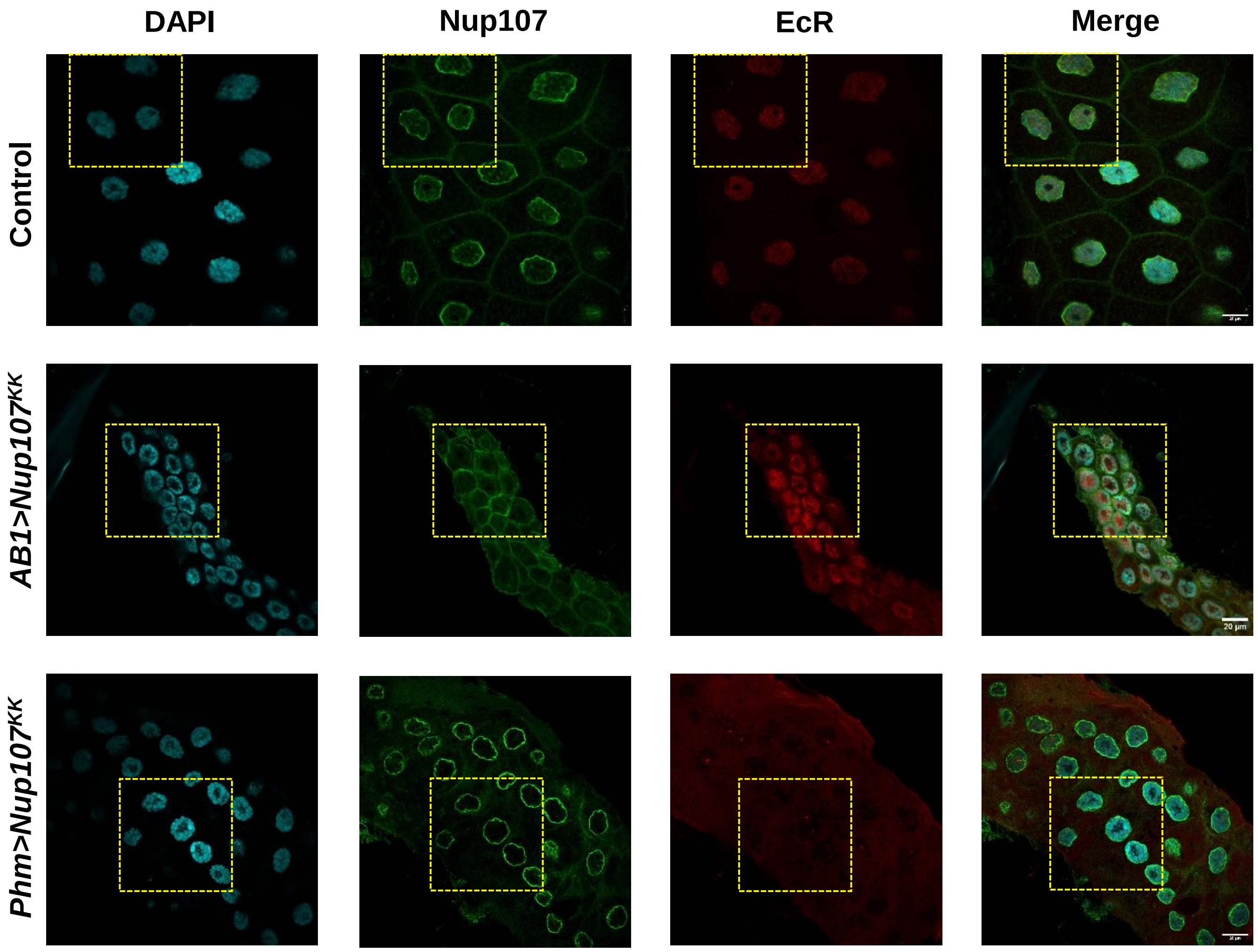

Figure 3, Source Data 1. Original images for Figures 3A, 3B, and 3C are shown. The cells highlighted in the yellow box were included in the main figure.
