# Supplementary material for: Nup107 is a crucial regulator of torso-mediated metamorphic transition in Drosophila melanogaster
Source: eLife. 2026 Mar 10;14:RP105165. doi: 10.7554/eLife.105165 (PMC12975125; doi:10.7554/eLife.105165)
Supplement: Figure 3—figure supplement 2—source data 1. — The upper panelc orresponds to AB1-Gal4, and the lower panel corresponds to Phm-Gal4. [file elife-105165-fig3-figsupp2-data1.pdf]

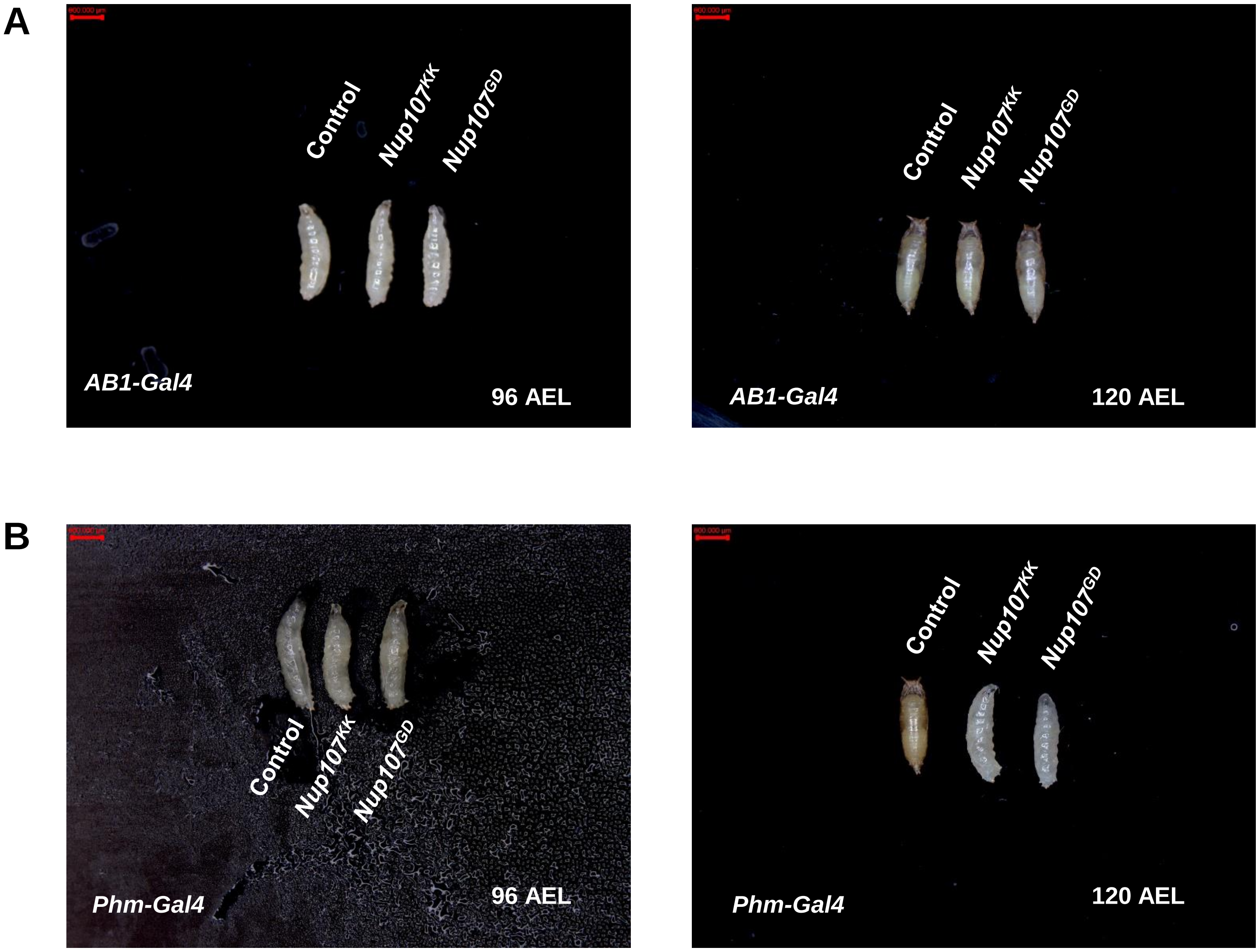

Figure 3- figure supplement 2, Source Data 1. Original images corresponding to Figure 3 and figure supplement 2. The upper panel corresponds to AB1-Gal4, and the lower panel corresponds to Phm-Gal4.
