# Supplementary material for: Nup107 is a crucial regulator of torso-mediated metamorphic transition in Drosophila melanogaster
Source: eLife. 2026 Mar 10;14:RP105165. doi: 10.7554/eLife.105165 (PMC12975125; doi:10.7554/eLife.105165)
Supplement: Figure 3—figure supplement 3—source data 1. [file elife-105165-fig3-figsupp3-data1.zip › Figure 3-figure supplement 3 Source data 1/Figure 3-figure supplement 3.pdf]

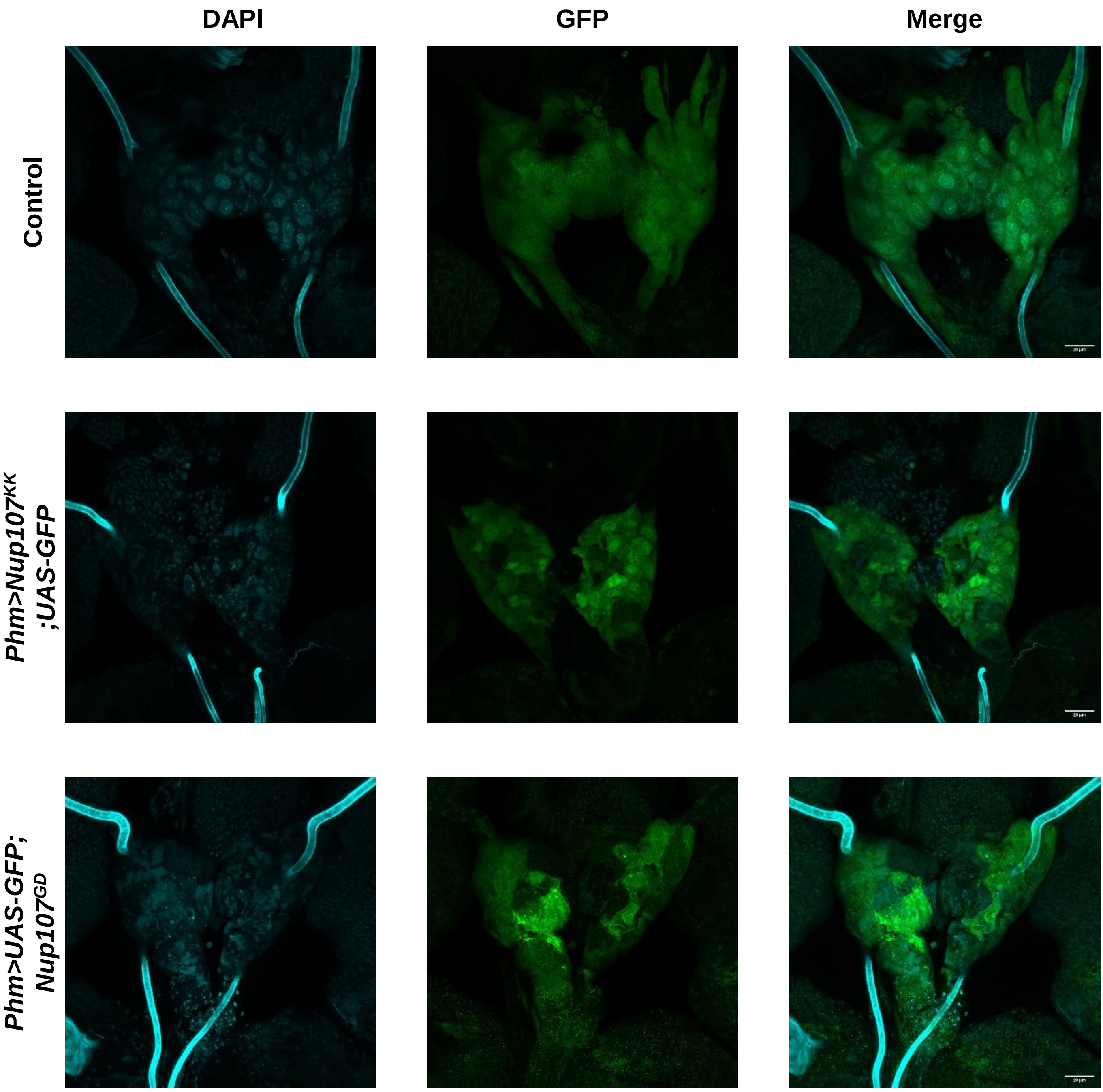

Figure 3- figure supplement 3, Source Data 1. The original confocal images of the prothoracic glands correspond to Figure 3- figure Supplement 3.
