# Supplementary material for: Nup107 is a crucial regulator of torso-mediated metamorphic transition in Drosophila melanogaster
Source: eLife. 2026 Mar 10;14:RP105165. doi: 10.7554/eLife.105165 (PMC12975125; doi:10.7554/eLife.105165)
Supplement: Figure 5—source data 1. [file elife-105165-fig5-data1.zip › Figure 5 source data 1/Figure 5 with 1 supplement.pdf]

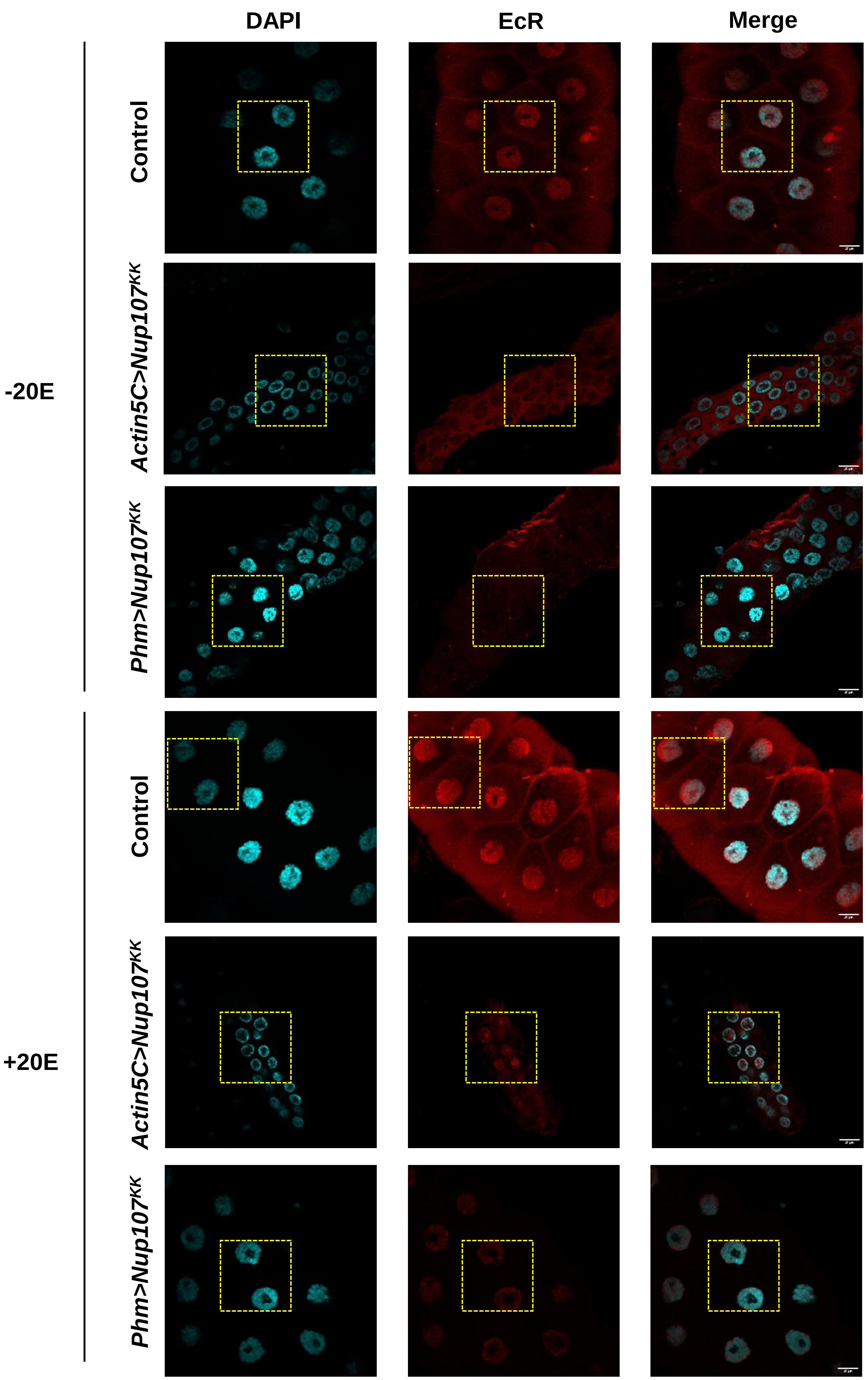

Figure 5, Source Data 1. Original images for without 20E (Figures 5A, 5B, and 5C) and with 20E (Figures 5D, 5E, and 5F) are shown. The cells highlighted in the yellow box were included in the main figure.
