# Supplementary material for: Nup107 is a crucial regulator of torso-mediated metamorphic transition in Drosophila melanogaster
Source: eLife. 2026 Mar 10;14:RP105165. doi: 10.7554/eLife.105165 (PMC12975125; doi:10.7554/eLife.105165)
Supplement: Figure 5—figure supplement 1—source data 1. — The cells highlighted in the yellow box were included in the supplementary figure. [file elife-105165-fig5-figsupp1-data1.zip › Figure 5- figure supplement 1 Source data 1/Figure 5-figure supplement 1.pdf]

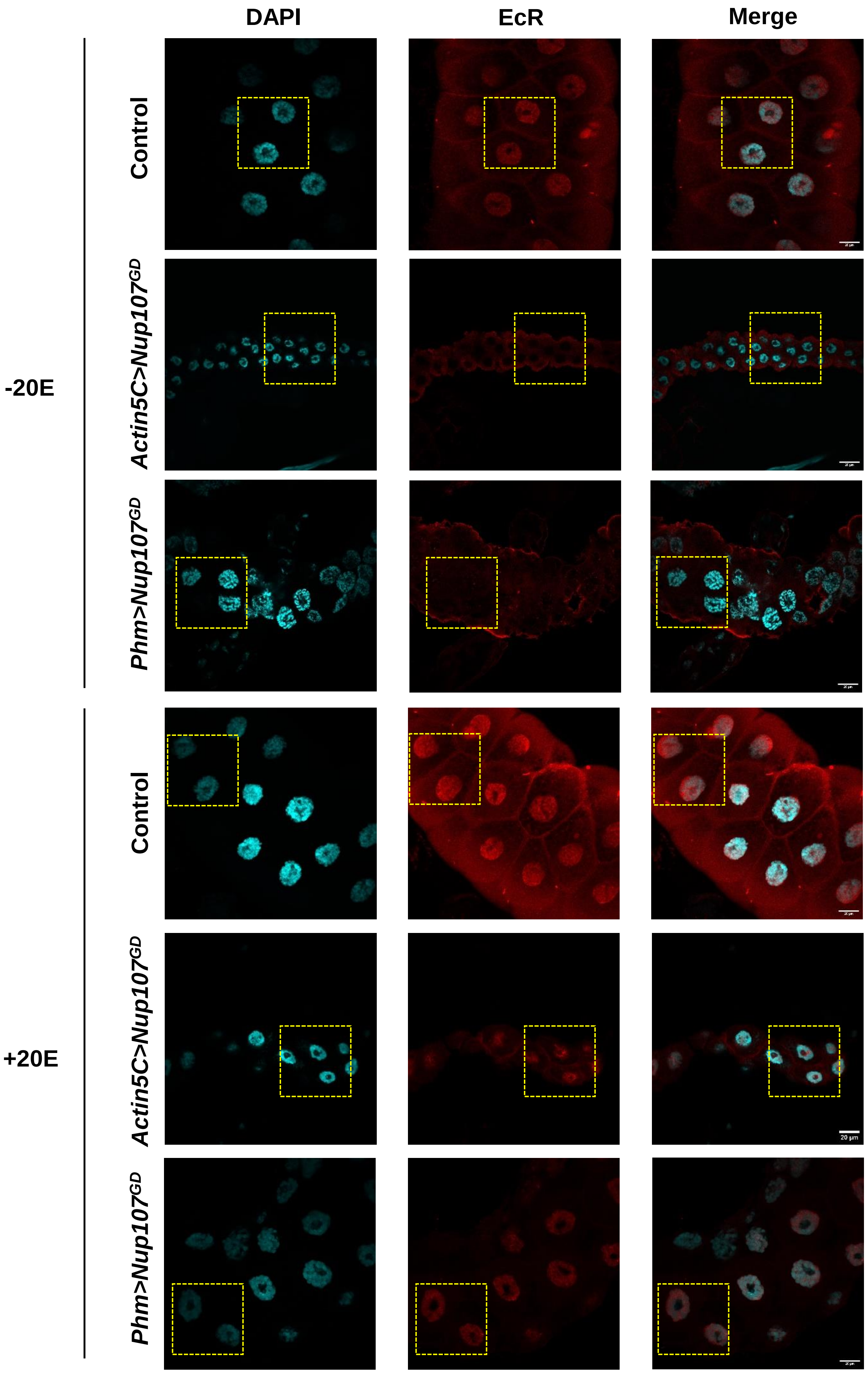

Figure 5- figure supplement 1, Source Data 1. Original images for without 20E (Figure 5- figure supplement 1A) and with 20E (Figure 5- figure supplement 1B) are shown. The cells highlighted in the yellow box were included in the supplementary figure.
