# Supplementary material for: Nup107 is a crucial regulator of torso-mediated metamorphic transition in Drosophila melanogaster
Source: eLife. 2026 Mar 10;14:RP105165. doi: 10.7554/eLife.105165 (PMC12975125; doi:10.7554/eLife.105165)
Supplement: Figure 6—source data 1. [file elife-105165-fig6-data1.zip › Figure 6 source data 1/Figure 6 with 1 supplement.pdf]

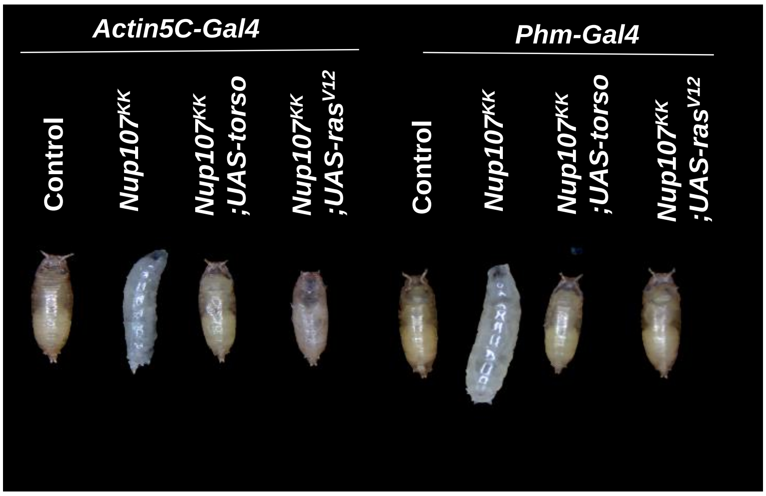

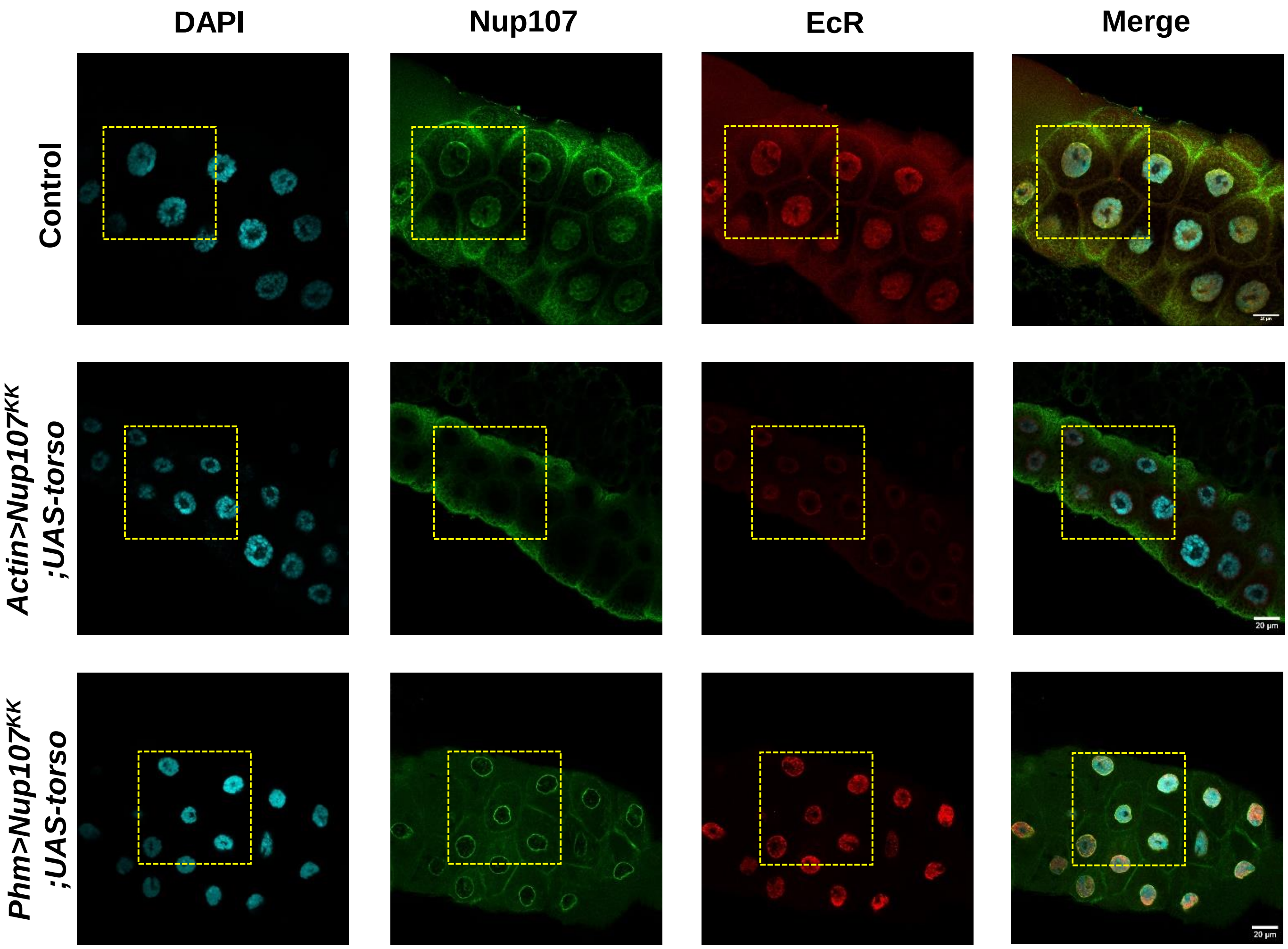

Figure 6, Source Data 1. Uncropped image of larvae and pupae corresponding to Figure 6C. Original confocal images for Figures 6E, 6F, and 6G are shown. The cells highlighted in the yellow box were included in the main figure.
