# Supplementary material for: Nup107 is a crucial regulator of torso-mediated metamorphic transition in Drosophila melanogaster
Source: eLife. 2026 Mar 10;14:RP105165. doi: 10.7554/eLife.105165 (PMC12975125; doi:10.7554/eLife.105165)
Supplement: Figure 6—figure supplement 1—source data 1. [file elife-105165-fig6-figsupp1-data1.zip › Figure 6- figure supplement 1 Source data 1/Figure 6-figure supplement 1.pdf]

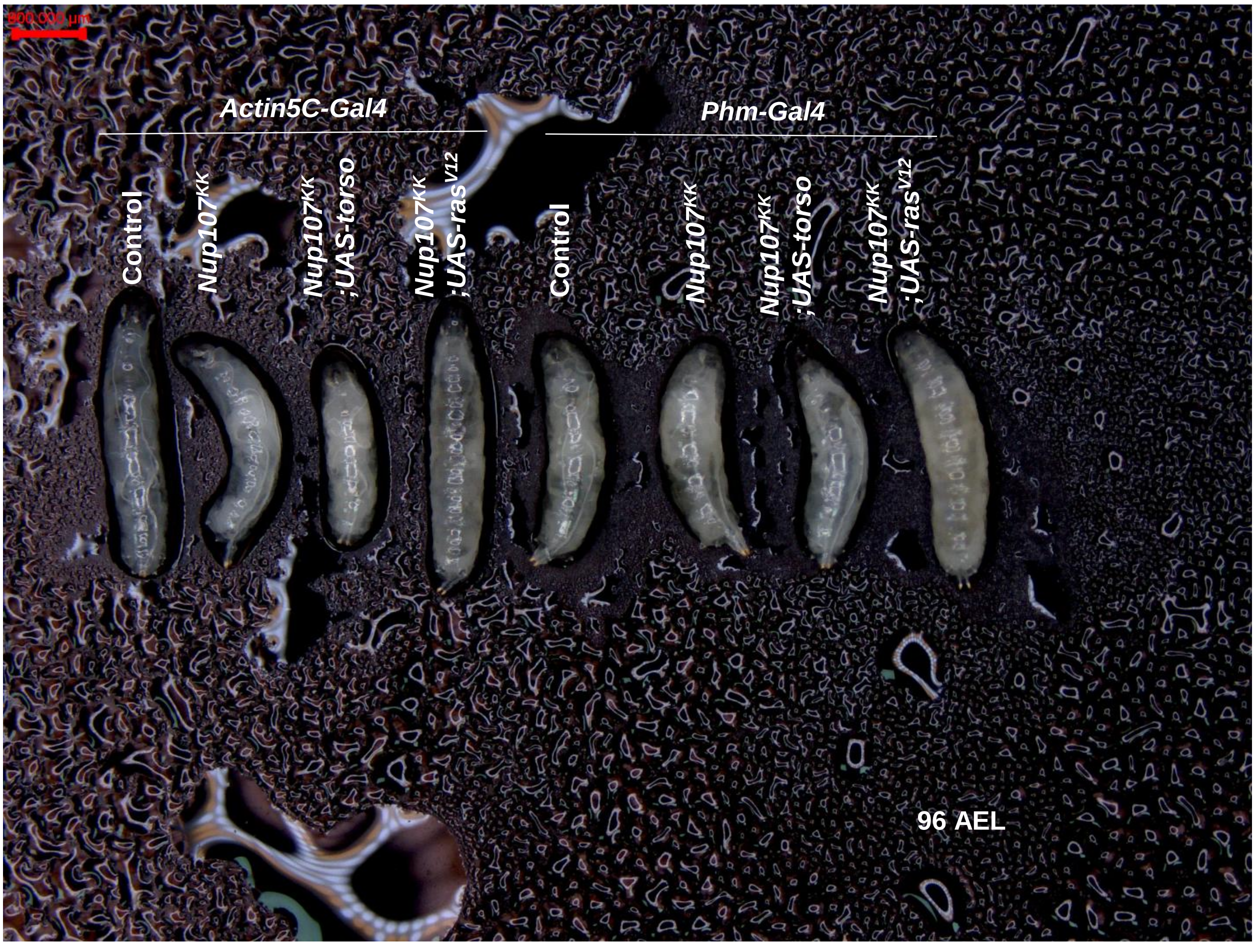

Figure 6- figure supplement 1, Source Data 1. Uncropped image of larvae corresponding to Figure 6- figure supplement 1.
